# Effects of high-protein supplementation during cancer therapy: a systematic review and meta-analysis

**DOI:** 10.1016/j.ajcnut.2024.08.016

**Published:** 2024-12-02

**Authors:** Camila E Orsso, Anne Caretero, Taiara Scopel Poltronieri, Jann Arends, Marian AE de van der Schueren, Nicole Kiss, Alessandro Laviano, Carla M Prado

**Affiliations:** 1 Human Nutrition Research Unit, Department of Agricultural, Food and Nutritional Science, University of Alberta, Edmonton, AB, Canada; 2 Faculty of Medicine, Federal University of Rio Grande do Sul, Porto Alegre, Brazil; 3 Department of Medicine I, Medical Center - University of Freiburg, Faculty of Medicine, University of Freiburg, Freiburg im Breisgau, Germany; 4 Department of Nutrition, Dietetics and Lifestyle, School of Allied Health, HAN University of Applied Sciences, Nijmegen, The Netherlands; 5 Department of Human Nutrition and Health, Wageningen University and Research, Wageningen, The Netherlands; 6 Institute for Physical Activity and Nutrition, Deakin University, Geelong, VIC, Australia; 7 Department of Translational and Precision Medicine, Sapienza University of Rome, Rome, Italy

**Keywords:** nutrition intervention, protein supplement, body weight, quality of life, body composition, cancer

## Abstract

**Background:**

Establishing the effectiveness of high-protein supplementation in reducing cancer-related side effects is crucial.

**Objective:**

The study aimed to assess the effectiveness and safety of high-protein supplementation on clinical outcomes of patients undergoing cancer therapy.

**Methods:**

Systematic searches were conducted on Medline, Cumulative Index to Nursing and Allied Health Literature (CINAHL), Embase, Cochrane Central Register of Controlled Trials, and Scopus from inception until July 2023. Randomized controlled trials administering supplements with ≥10 g protein/serving, given to 20+ adult patients undergoing cancer therapy were included. Random-effects meta-analyses were used to estimate the effects of high-protein supplementation on the primary outcomes of body weight and health-related quality of life (HRQoL). We employed a vote-counting approach based on effect direction for secondary outcomes (that is, body composition, muscle function, hospitalization, response to cancer therapy/toxicity, survival, and systemic inflammation). Risk-of-bias (ROB) was assessed.

**Results:**

Thirty-five studies involving 3701 patients with diverse cancer types were included. Patients who received high-protein supplementation lost less body weight than controls (mean difference = 1.45 kg; 95% CI: 0.42, 2.48 kg; *P* = 0.006; *I*^*2*^ = 80%). No differences in HRQoL were observed; all studies assessing HRQoL were rated as high ROB. A beneficial effect on muscle mass was found in 11 of 13 studies, although most had a high ROB due to assessment techniques. When considering higher quality studies, evidence of a beneficial effect was found in 5 of 5 studies for muscle strength, and 3 of 4 for hospitalization rate. Effects on other secondary outcomes were inconsistent or limited. No serious adverse effects were reported.

**Conclusions:**

High-protein supplementation mitigates weight loss, improves muscle strength, and lowers hospitalization rates in patients undergoing cancer therapy. These positive clinical outcomes, along with a favorable safety profile, suggest that high-protein supplementation may be a valuable addition to medical practice. However, given the need for more robust trials and the high ROB observed in the existing studies, these conclusions should be interpreted with caution.

This review was prospectively registered with PROSPERO under the registration number CRD42021237372.

## Introduction

Global cancer burden is rising [1], accounting for 1 in 6 deaths [2]. Cancer impacts patient’s lives and strains families and healthcare systems [[3], [4], [5], [6], [7]]. Patients with cancer often experience side effects, regardless of disease type, stage, and prediagnosis health status [8], which include nutrition impact symptoms, fatigue, and depression, among others. These side effects can lead to weight loss, deterioration in health-related quality of life (HRQoL), and unfavorable body composition and muscle function changes, resulting in poor clinical outcomes and overall prognosis [[9], [10], [11], [12], [13]].

Cancer proinflammatory state accelerates muscle catabolism and reduces muscle protein synthesis, leading to muscle loss [[14], [15], [16]]. This increased catabolic state, coupled with decreased food intake and nutrient absorption, underscores the critical need for nutrition interventions, particularly increasing protein consumption. Protein requirements for patients with cancer are set at higher levels compared with the general population [17] as a minimum of 1.0 g protein/kg body weight/d, with a target consumption of 1.2−2.0 g/kg/d [18,19]. However, attaining such protein intake through dietary means can be difficult [17,20]. Hence, protein supplementation, especially when combined with personalized dietary advice, is a viable strategy [21]. This approach ensures adequate protein intake and addresses individual nutritional needs effectively.

A 2018 systematic review explored the effects of protein- and omega-3-enriched oral nutritional supplements (omega-3 ONS) on patients undergoing treatment with or without nutrition counseling [21]. Omega-3 ONS reduced muscle loss (assessed as fat-free mass or lean mass) in half the trials and improved HRQoL domains in 3 out of 4 studies. Although ONS are nutritionally complete and recommended in nutritional oncology guidelines [18,19], research on alternative high-protein supplements [for example, branched-chain amino acids (BCAA), glutamine, arginine] is limited [18].

Considering the emerging literature, an updated and comprehensive review is needed to explore the effects of high-protein supplementation (≥10 g protein/serving) on patients with cancer. We conducted a systematic review and meta-analysis of randomized controlled trials (RCTs), evaluating the effectiveness and safety of high-protein supplementation in patients being actively treated for cancer. High-protein supplementation consisted of single or mixed amino acids, which may contain or was enriched with omega-3 fatty acids, protein precursors, and modulators of protein metabolism. This analysis covered several outcomes, including body weight and HRQoL as primary outcomes and body composition, muscle function, survival, hospitalization, response to cancer therapy/toxicity, and systemic inflammation as secondary outcomes.

## Methods

This review followed the PRISMA [22] and the Synthesis Without Meta-analysis (SWiM) reporting guidelines [23]. The research protocol was registered with PROSPERO (CRD42021237372).

### Eligibility criteria

We included RCTs comparing high-protein supplementation (≥10 g of protein/serving) to placebo, standard of care, or lower dose protein supplements among patients receiving treatment for any cancer type (Supplemental Material 1). High-protein supplementation in the form of food supplements, ONS, or specialized ONS was included, with or without nutrition counseling or dietary advice. Studies on tube feeding alone or parenteral nutrition were excluded. Studies including exercise intervention were ineligible. For patients undergoing surgery, high-protein supplementation had to be administered perioperatively. We only included studies that reported ≥1 of our primary or secondary outcomes or explored intervention safety.

### Outcomes

Primary outcomes were body weight (or BMI, when weight was unavailable) and HRQoL. Body weight assessed physical health, whereas HRQoL evaluated the broader impact of protein supplementation on the patient's overall well-being. Secondary outcomes included muscle and fat masses, muscle function, survival, hospitalization, response to cancer therapy/toxicity, and systemic inflammation. Although therapy administration outcomes, including chemotherapy modifications and treatment delays, are often regarded as toxicity-related outcomes [24], we presented our findings on these outcomes separately from cancer therapy-induced toxicity. Systemic inflammation was evaluated as an outcome in this review; however, studies that assessed inflammatory markers to report patient profiling (characteristics) and/or as an outcome of the intervention were included. Specifically, the markers and proxies evaluated included albumin, lymphocyte count, c-reactive protein, prealbumin, IL-6, transferrin, LPS-binding protein, leukocytes, eosinophils, cortisol, hemoglobin, tumor necrosis factor-alpha, neutrophil-to-lymphocyte ratio, platelet-to-lymphocyte ratio, white blood cells, and red blood cells.

### Search strategy and data extraction

We searched Medline (via Ovid), Cumulative Index to Nursing and Allied Health Literature (CINAHL), Embase (via Elsevier), Cochrane Central Register of Controlled Trials, and Scopus from inception to 5 July, 2023 (last search date). Terms related to “food and oral nutritional supplements,” “oncology,” and “RCTs” were included, limited to English language, humans, and adult studies (Supplemental Material 2). ClinicalTrials.gov, Google, and reference lists of retrieved reports were also searched. Records were imported to Covidence (Veritas Health Innovation Ltd) for automated deduplication and study selection by 2 independent reviewers.

One reviewer extracted data on study characteristics and primary outcomes using Covidence Extraction 2 or an online spreadsheet in Google Sheets for secondary and safety outcomes. Effect sizes and corresponding *P* values were collected for primary and secondary outcomes, along with baseline and postintervention data. We used data from the closest to the conclusion of the intervention for multiple follow-up evaluations and extracted graphical data using Plot Digitizer (V.2.6.9; http://plotdigitizer.sourceforge.net) [25]. For primary outcomes, we collected the mean and SD of absolute or relative changes, if available, or the mean and SD of postintervention values. When required, median and interquartile data were transformed to mean and SD, or SD was estimated from *P* values of differences between groups [26]. Similar interventions were combined for multiple intervention arms studies to form a single pair-wise comparison [27]. We attempted to contact study authors for missing data and sought information on the nutritional composition of the supplement from pertinent websites or manufacturing companies. Data were cross-checked for accuracy by 2 reviewers, and discrepancies were resolved through consensus. Note that the number of studies reported may not reflect the number of citations because some studies may have been documented in multiple publications.

### Risk-of-bias assessment

Two independent reviewers evaluated risk-of-bias (ROB) for each outcome domain using the Revised Cochrane ROB tool for randomized trials (RoB2) [28], which examines bias due to randomization, deviations from intended interventions, missing outcome data, outcome methods, and selective reporting of results. Results were represented graphically using ROB VISualization (robvis) [29].

### Statistical analysis

#### Meta-analysis

We conducted random-effects meta-analysis to evaluate the weighted average intervention effect on our primary outcomes, along with 95% confidence intervals (CIs) in Review Manager 5 version 5.4.1 (The Cochrane Collaboration). Effect estimates of body weight in kilograms were determined using mean difference (MD). Sensitivity analysis combined studies assessing body weight, percentage change, and BMI using the standardized mean difference (SMD). We estimated the effect size of HRQoL using SMD, owing to using different assessment tools. We assessed heterogeneity using *I*^2^ statistics and set statistical significance at *P* = 0.033 for multiple outcomes. When data for ≥2 studies were available, subgroup analyses were conducted to explore the influence of tumor type, cancer therapy, supplement type, protein dose, duration of high-protein supplementation, preintervention weight loss and (risk of) malnutrition, systemic inflammation changes, adherence, and ROB on the primary outcomes. Studies not included in statistical pooling were summarized narratively.

#### Synthesis without meta-analysis

Intervention effects on secondary outcomes were synthesized using vote-counting, focusing (solely) on the direction of effect [23,30]. We categorized the direction of effect as “beneficial effect” when the intervention positively influenced health or resulted in an unchanged outcome. Conversely, “no beneficial effect” was attributed to the negatively influenced health. For studies with multiple related outcomes, “beneficial effect” or “no beneficial effect” was defined if ≥70% of outcomes showed a consistent direction; those with <70% had a “mixed effect” [31]. Results were represented graphically using Harvest plots [[31], [32], [33]]; effect estimates and *P* values for each individual study were provided. Sensitivity analysis, using combined data from studies with low and moderate ROB, was also conducted [34].

## Results

**FIGURE 1:**
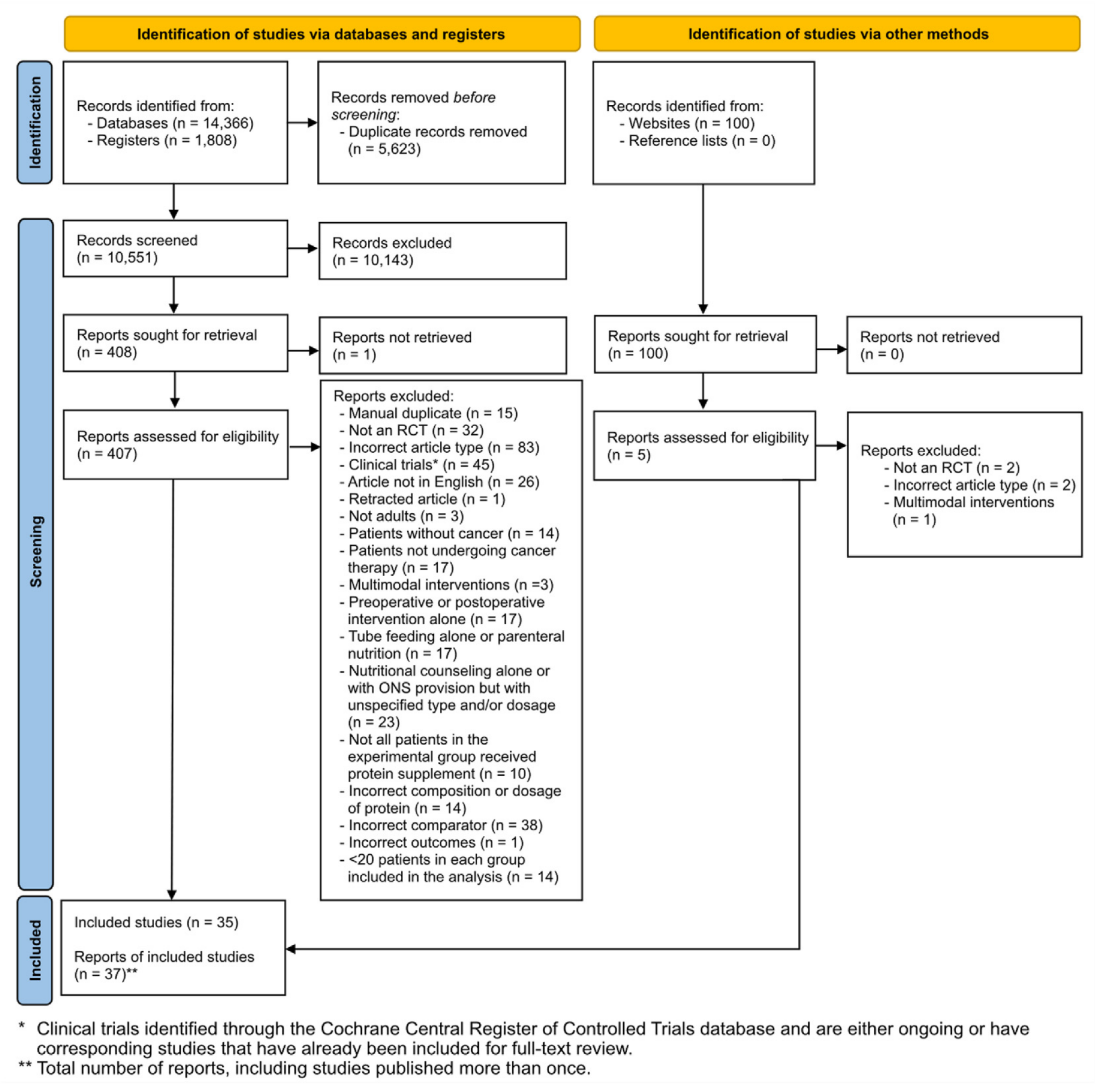
PRISMA 2020 flow diagram for study selection.

A total of 16,274 records were originally identified and 412 full-text reports were reviewed, with 37 meeting eligibility criteria; these yielded findings from 35 unique studies (Figure 1). This discrepancy occurred because 4 studies reported different outcomes for the same study [[35], [36], [37], [38]]. Studies were published between 1998 and 2023.

### Study characteristics

Study characteristics are summarized in Supplemental Table 1. Included studies were conducted across 22 different countries, with most from Japan (7 studies; 20%) [[37], [38], [39], [40], [41], [42], [43], [44]], China (4 studies; 11%) [[45], [46], [47], [48]], and the United Kingdom (3 studies; 9%) [[49], [50], [51]]. Among these, 24 (69%) studies had an open-label design [[35], [36], [37], [38], [39], [40], [41], [42], [43],[45], [46], [47],^49^,[52], [53], [54], [55], [56], [57], [58], [59], [60], [61], [62], [63], [64]], whereas 7 (20%) were double-blinded [44,50,51,[65], [66], [67], [68]], and 3 (9%) were single-blinded [48,69,70]. Additionally, 25 studies (71%) were conducted at a single center [35,36,[40], [41], [42], [43], [44], [45], [46],^48^,[51], [52], [53], [54], [55],^57^,[59], [60], [61], [62],^64^,^65^,[67], [68], [69], [70]], whereas 8 (23%) were multicenter trials involving 2–16 centers [[37], [38], [39],^47^,^49^,^50^,^56^,^58^,^66^].

Studies included 3701 patients, with experimental group sizes ranging from 22 to 171 individuals and the control group from 23 to 166. The mean or median age of patients in the experimental group ranged from 44.1 to 69.1 y, and in the control group from 44.4 to 70.6 y. At the time of nutrition intervention, patients were diagnosed with gastrointestinal tract (25 studies; 71%) [[35], [36], [37], [38], [39], [40], [41], [42], [43], [44], [45], [46], [47], [48], [49], [50],^54^,^56^,[58], [59], [60], [61],^63^,^65^,^67^,^71^], lung (6 studies; 17%) [49,53,56,66,68,69], breast (4 studies; 11%) [51,52,56,70], gynecological (4 studies; 11%) [55,56,58,59], head and neck (4 studies; 11%) [57,58,63,64], mesothelioma (1 study; 3%) [49], and bladder cancer (1 study; 3%) [62] (Supplemental Figure 1). Cancer stages ranged from I to III (2 studies; 6%) [37,38,52], II (1 study; 3%) [43], I–IV (10 studies; 29%) [35,36,40,45,46,53,55,57,62,64,66], and III–IV (3 studies; 9%) [39,68,69]. Studies included patients undergoing chemotherapy (14 studies; 40%) [39,40,45,46,49,51,52,54,56,59,63,66,69,70], surgery (12 studies; 34%) [37,38,42,44,47,50,53,55,[60], [61], [62],^65^,^71^], concurrent chemoradiotherapy (7 studies; 20%) [35,36,43,56,58,59,67,68], radiotherapy (3 studies; 9%) [57,59,64], or transarterial chemoembolization (2 studies; 6%) [41,48].

Patients in both the experimental and control groups exhibited nutritional vulnerabilities, including risk of malnutrition or malnutrition (8 studies; 23%) [35,36,45,46,54,55,64,65,67] or pre-cachexia (1 study; 3%) [54]. Preintervention weight loss was documented for all patients or in a proportion of them in 10 (29%) studies and categorized as <5% weight loss [59,61], ≥5% weight loss [50,59], <10% weight loss [54,55,66,69], or > 10% weight loss [48,50,69,71] over 1–12 mo before intervention in most studies; no significant preintervention weight loss was reported in 1 study [57]. Preintervention protein intake was reported in 9 studies [35,36,50,52,56,57,59,62,64,69], with values ranging from 0.83 to 1.16 g/kg/d in the experimental group and 0.83 to 1.11 g/kg/d in the control group. Total energy intake at baseline was reported in 10 studies [35,36,43,48,52,56,57,59,62,64,69], with values ranging from 23.3 to 28.8 kcal/kg/d in the experimental group and 22.3 to 30.9 kcal/kg/d in the control group.

Studies used various types of high-protein supplementation, including high-protein ONS (11 studies) [35,36,40,[45], [46], [47],^49^,^54^,^55^,^57^,^64^,^65^]; omega-3 ONS (9 studies) [[37], [38], [39],^43^,^50^,^52^,^56^,^59^,^66^,^69^]; glutamine (4 studies) [63,67,68,70]; ONS containing arginine and omega-3 or omega-6 (Arg/omega-3, -6 ONS; 4 studies) [60,61,65,71]; BCAA (3 studies) [41,42,48]; β-hydroxy β-methylbutyrate (HMB) combined with arginine and glutamine (HMB/Arg/Gln; 2 studies) [44,53]; arginine (1 study) [51]; ONS containing arginine, glutamine, and omega-3 (Arg/Gln/omega-3 ONS; 1 study) [58]; and ONS containing HMB and omega-3 (HMB/omega-3 ONS; 1 study) [62] (Supplemental Figure 2). The highest protein content per supplement serving was 25 g [47], with most studies (20 in total) administering 2 daily servings [[35], [36], [37], [38], [39], [40],^42^,^43^,^45^,^46^,^48^,[53], [54], [55],^58^,^60^,^62^,[64], [65], [66],^69^,^70^]. Sixteen studies combined high-protein supplementation with other nutrition interventions, such as nutrition counseling or dietary advice [43,45,46,49,52,[56], [57], [58], [59], [60]], standard/routine nutrition care [44,47,48,53,62], and standardized menus [69]. The standard/routine nutrition care varied across studies. It included the following: isocaloric juice plus a regular hospital diet [44], regular hospital diet with a target energy requirement of 20–30 kcal/kg/d [47], a usual hospital diet [48], a usual hospital diet with target energy calculated by Harris–Benedict formula and protein according to the dietitian's prescription [53], and twice daily multivitamins [62].The goals of nutrition counseling or dietary advice varied and aimed at achieving a high-protein, plant-based diet [52], increasing the intake of fat and protein-rich foods [45,46], attaining an isocaloric diet [56], meeting protein and energy requirements [59], or prevent under-nutrition [58]. Length of intervention for patients undergoing surgery ranged from 3 to 4 wk [62] preoperatively to 6 mo [42] postoperatively; for those receiving other cancer therapies, the intervention lasted between 5 d [70] and 1 y [48]. In most studies, controls received standard/routine nutrition care (8 studies) [37,38,47,48,53,55,62,64,68] and neither supplementation nor intervention (8 studies) [35,36,[39], [40], [41],^49^,^54^,^64^,^71^].

### Meta-analyses of primary outcomes

#### Body weight

**FIGURE 2:**
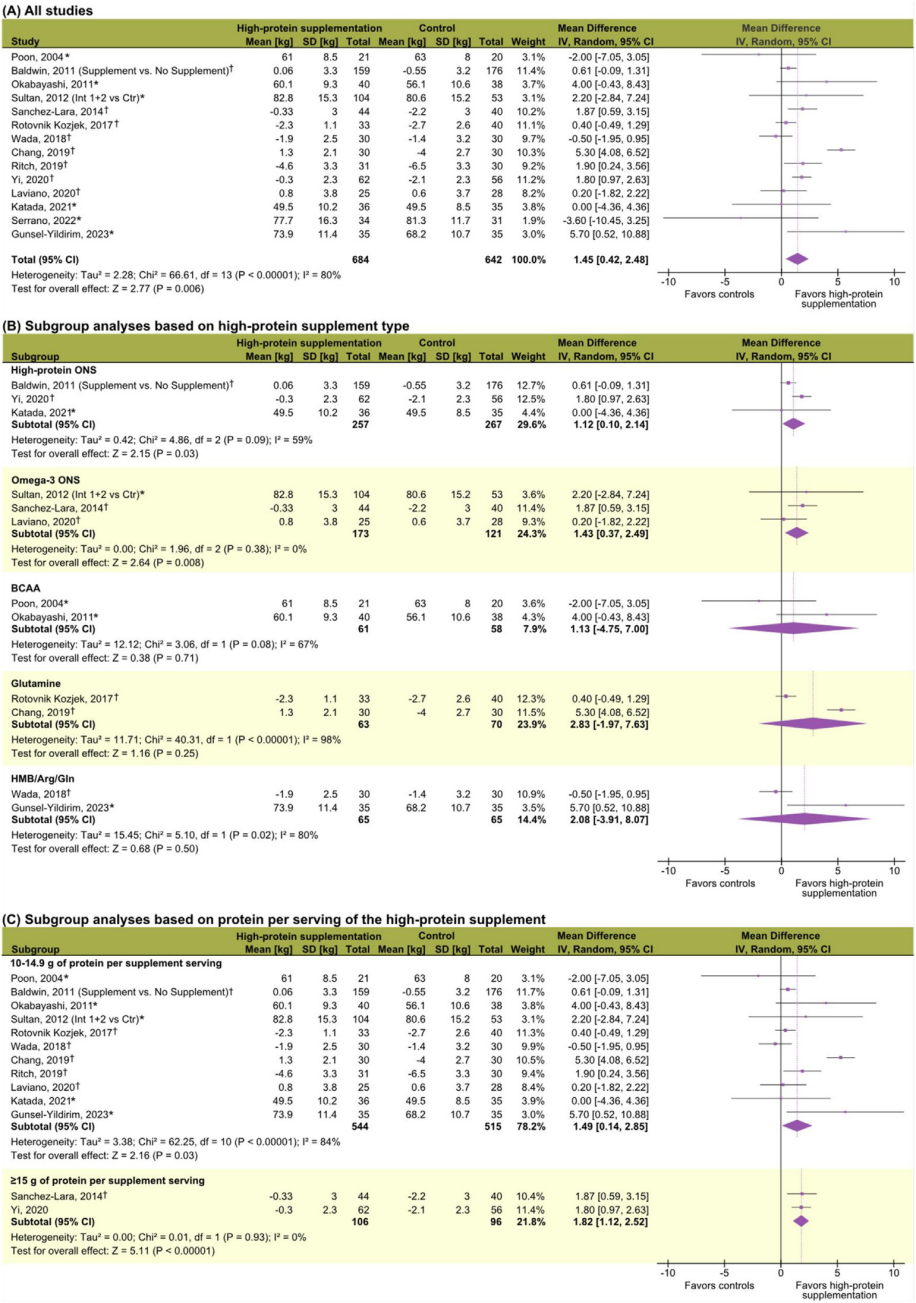
**Meta-analyses of the effects ofhigh-proteinsupplementation on body weight (all units of measurement are in kilograms).** (A) All included studies. (B) Subgroup analyses based on supplement type. (C) Subgroup analyses based on protein content per serving per day. The summary statistic table displays postintervention values (∗) or absolute changes (^†^) based on available data, resulting in varying magnitudes. The study by Baldwin et al. [49] combined study groups when reporting body weight changes; thus, the “Nutrition supplement” group included those patients who received supplements alone or concurrently with nutritional counseling, and the “No nutrition supplement” group included those who did not receive any intervention nor nutritional counseling. CI, confidence interval.

Twenty of 23 (87%) studies examining body weight (or BMI) were included [37,[40], [41], [42], [43], [44],[48], [49], [50],[52], [53], [54], [55],^57^,^62^,[65], [66], [67], [68], [69]]. Patients receiving high-protein supplementation lost less body weight than controls, with a pooled MD of 1.45 kg (95% CI: 0.42, 2.48 kg; *P* = 0.006; *I*^*2*^ = 80%) (Figure 2). Mean absolute changes in body weight ranged from −4.6 to 1.3 kg in the experimental group, and −6.5 to 0.6 kg in the control group. Combining body weight data of different units of measurement resulted in a significant SMD of 0.22 (95% CI: 0.03, 0.41; *P* = 0.02; *I*^*2*^ = 74%) (Supplemental Figure 3). A significant effect was observed with high-protein ONS and omega-3 ONS, whereas no significant effects were detected for BCAA, glutamine, and HMB/Arg/Gln (Figure 2). Studies using supplements containing either 10–14.9 g of protein/serving or ≥15 g of protein/serving (Figure 2), or providing a total daily protein intake of ≥40 g (MD = 1.81 kg; 95% CI: 0.99, 2.63 kg; *P <* 0.001; *I*^*2*^ = 0%) (Supplemental Figure 4) also had a significant effect on body weight. Furthermore, a significant effect was observed in studies that administered high-protein supplementation for 3–12 wk (MD = 0.82 kg; 95% CI: 0.24, 1.40; *P* = 0.006; *I*^2^ = 20%) and for those that continued beyond 13 wk (MD = 5.21 kg; 95% CI: 4.03, 6.39) (Supplemental Figure 4). Subgroup analyses revealed a positive intervention effect for patients with lung cancer, chemotherapy and surgery recipients, preintervention weight losers, and patients with lower systemic inflammation following intervention (Supplemental Figures 5 and 6).

Studies excluded from meta-analyses failed to report variance data [56] or only reported weight loss frequency [39,59]. In these studies, concurrent omega-3 ONS supplementation and nutrition counseling resulted in increased body weight [56] or in a lower frequency of weight loss compared with controls [59]; however, when omega-3 ONS was administered alone, more patients lost weight in the experimental group [39].

#### Health-related quality of life

**FIGURE 3:**
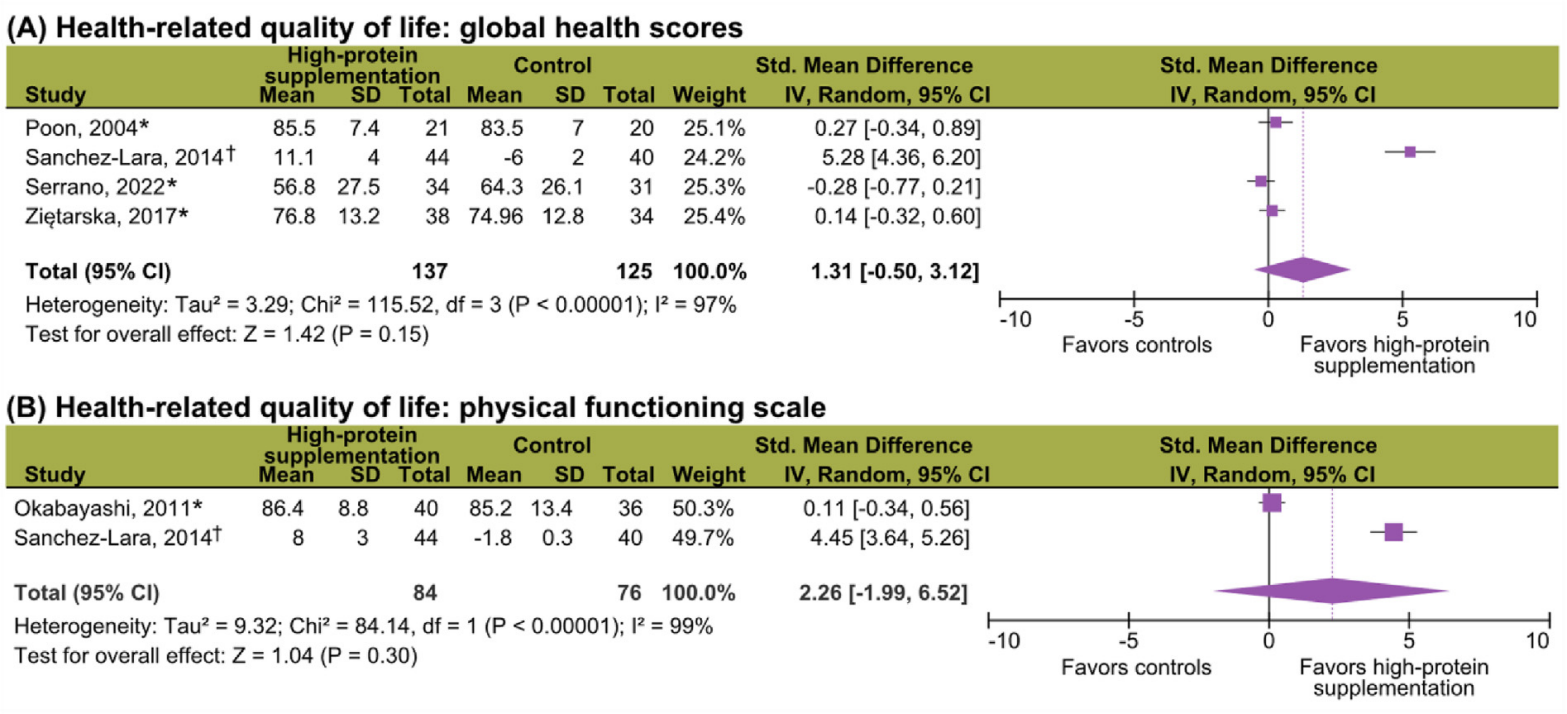
**Meta-analyses of the effects ofhigh-proteinsupplementation onhealth-relatedquality of life.** (A) Global health scores (all studies included in the analysis). (B) Physical functioning scale (all studies included in the analysis). The summary statistic table displays postintervention values (∗) or absolute changes (^†^) based on available data, resulting in varying magnitudes. HRQoL was assessed using the European Organization for Research and Treatment of Cancer Quality of Life Questionnaire (EORTC QLQ-C30) [65,69], the Functional Assessment of Cancer Therapy - General (FACT-G) [48,65], the 36-Item Short Form Survey (SF-36) [42], and the Functional Assessment of Anorexia/Cachexia Treatment (FAACT) [54]. CI, confidence interval.

Five of 10 (50%) studies exploring HRQoL were included [42,48,54,65,69]. High-protein supplementation had no significant effect on global health scores (SMD = 1.31; 95% CI: −0.50, 3.12; *P* = 0.15; *I*^*2*^ = 97%) and the physical functioning domain (SMD = 2.26; 95% CI: −1.99, 6.52; *P* = 0.30, *I*^*2*^ = 99%) (Figure 3); heterogeneity was high in both analyses. Excluded studies failed to report variance data [35,36,64] or only reported on significant differences [49,56,59]. Of these, Ravasco et al. [35,36,64] reported improved global and function scores with high-protein ONS, with no significant intervention effect for the remaining studies [49,56,59].

### Synthesis without meta-analyses of secondary outcomes

#### Muscle mass

**FIGURE 4:**
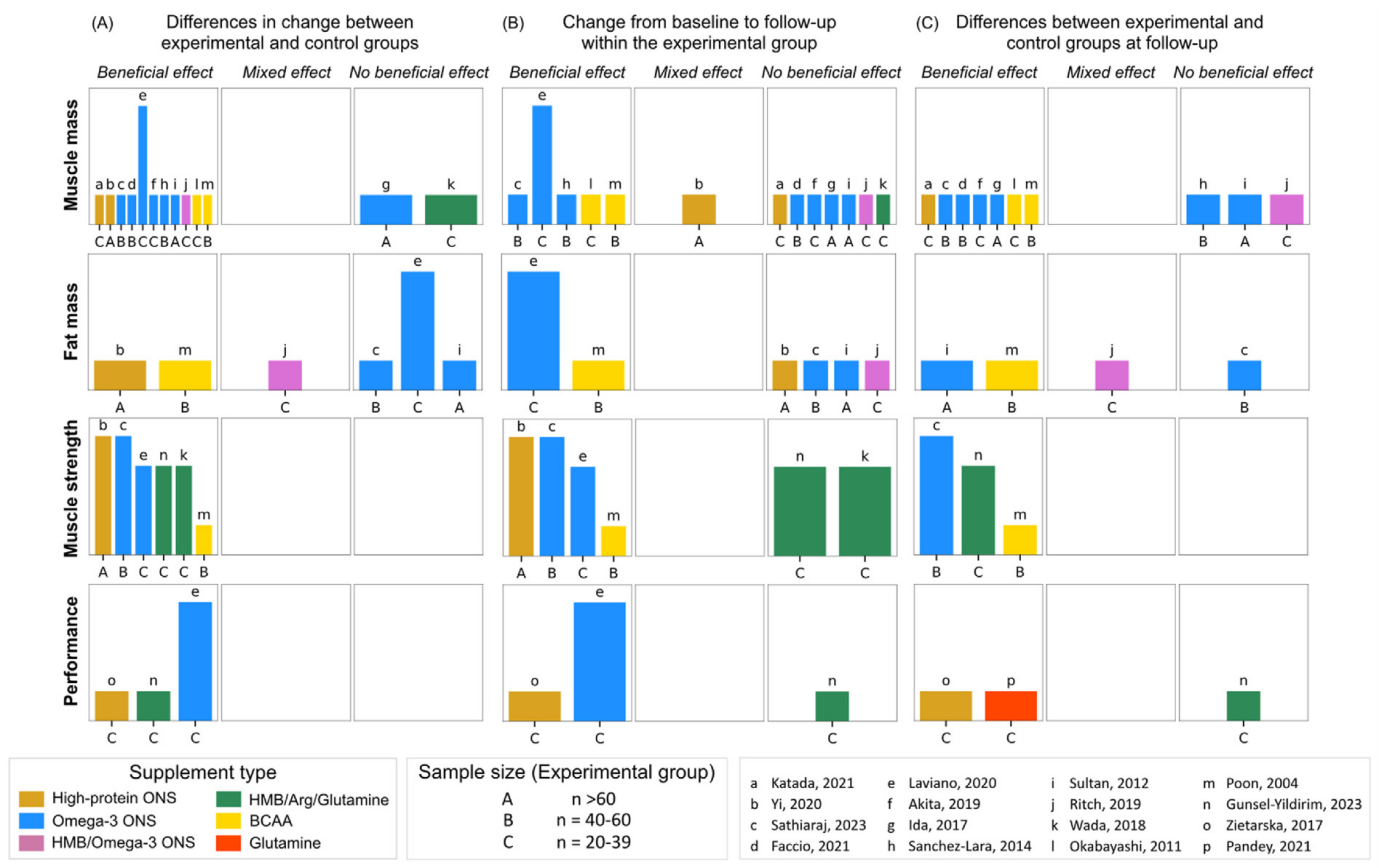
**Harvest plots summarizing the effects ofhigh-proteinsupplementation on muscle mass, fat mass, and muscle function (muscle strength and performance).** (A) Describes the effects by comparing changes in outcomes between experimental and control groups. (B) Indicates the effects within experimental group considering changes from baseline to follow-up. (C) Represents the effects based on differences between experimental and control groups at follow-up. The height of each bar served as an indicator of study quality, where taller bars signify a lower risk-of-bias, medium-height bars denote a moderate risk-of-bias, and shorter bars signify a high risk-of-bias. Each lowercase letter corresponds to a unique study, while uppercase letters indicate the sample size of experimental groups. Different supplement types are represented by varying colors. Arg, arginine; BCAA, branched-chain amino acids; Gln, glutamine; HMB, β-hydroxy β-methylbutyrate; ONS, oral nutritional supplement.

Muscle mass (or its related compartments) was evaluated in 13 studies [38,40,42,43,44,48,50,52,55,56,62,66,69]. Various body composition techniques were used, such as bioelectrical impedance analysis [38,40,43,44,52,55,56,69], computed tomography [43,62], and dual-energy X-ray absorptiometry [66] (Supplemental Figure 7). Mid-arm muscle circumference was used as a surrogate anthropometric measure of muscle mass in 4 studies [42,48,50,55]. Skeletal muscle radiodensity was also evaluated in 1 study [62]. Overall, 11 of 13 (85%) studies showed that high-protein supplementation had a beneficial effect on muscle mass and skeletal muscle radiodensity compared with controls [40,42,43,48,50,52,55,56,62,66,69] (Figure 4, Supplemental Figure 8). Although a decline in muscle mass was experienced in both groups, the experimental group lost less muscle mass than controls.

A diverse array of protein supplements exhibited this beneficial effect, encompassing high-protein ONS [40,55], omega-3 ONS [43,50,52,56,66,69], BCAA [42,48], and ONS enriched with HMB and omega-3 [62]. HMB, administered with amino acids arginine and glutamine, had no beneficial effects on muscle mass [44]. To assess how inflammation influences the effectiveness of high-protein supplementation on muscle mass, we conducted a subgroup analysis on 10 studies [37,38,40,42,48,50,55,56,62,66,69] assessing changes in systemic inflammation and muscle mass; 60% of the studies found that patients with worsened inflammation also experienced muscle loss [37,38,40,50,55,56,62]. Conversely, 20% of the studies observed muscle gains associated with reduced systemic inflammation [42,69].

#### Fat mass

Three studies evaluated fat mass using either bioelectrical impedance analysis or dual-energy X-ray absorptiometry [52,55,66], whereas 2 studies assessed triceps skinfolds as a surrogate of fat mass [48,50] (Supplemental Figure 7). Additionally, 1 study used computed tomography scans to quantify visceral and subcutaneous adipose tissues [62]. Overall, 3 of 6 studies (50%) found no beneficial effect of high-protein supplementation on fat mass, compared with control groups [50,52,66] (Figure 4, Supplemental Figure 9).

Results varied across supplement types and analyses. Three of 3 studies (100%) reported that omega-3 ONS resulted in reduced fat mass (that is, no beneficial effect) in patients undergoing chemotherapy [52,66] or triceps skinfolds in surgical patients [50], as compared with control groups. BCAA supplementation consistently effected fat mass across analyses during transarterial chemoembolization in 1 study [48]. However, the effects of perioperative supplementation with high-protein ONS and HMB/omega-3 ONS on fat mass [55] and visceral and subcutaneous adipose tissues [62], respectively, varied across analyses.

#### Muscle function

Compared with controls, high-protein supplementation was associated with a beneficial effect on handgrip strength in 6 of 6 studies (100%) [44,48,52,53,55,66] (Figure 4, Supplemental Figure 10). Supplements included high-protein ONS [55], omega-3 ONS [52,66], HMB/Arg/glutamine [44,53], and BCAA [48]. A beneficial effect of high-protein supplementation compared with controls was also observed on physical performance outcomes in 3 of 3 studies (100%) [53,54,66]. Among these studies, high-protein ONS demonstrated a beneficial effect on Karnofsky Performance Scale [54], and omega-3 ONS showed a beneficial effect on walking distance [66] in patients receiving neoadjuvant chemotherapy. Patients undergoing surgery who received HMB/Arg/Gln supplementation showed less increase in the Eastern Cooperative Oncology Group Performance Status scale scores than controls, indicating a beneficial effect [53]. Additionally, glutamine supplementation during neoadjuvant or adjuvant chemotherapy was also positively associated with Karnofsky Performance Scale scores at follow-up [70].

#### Survival

**FIGURE 5:**
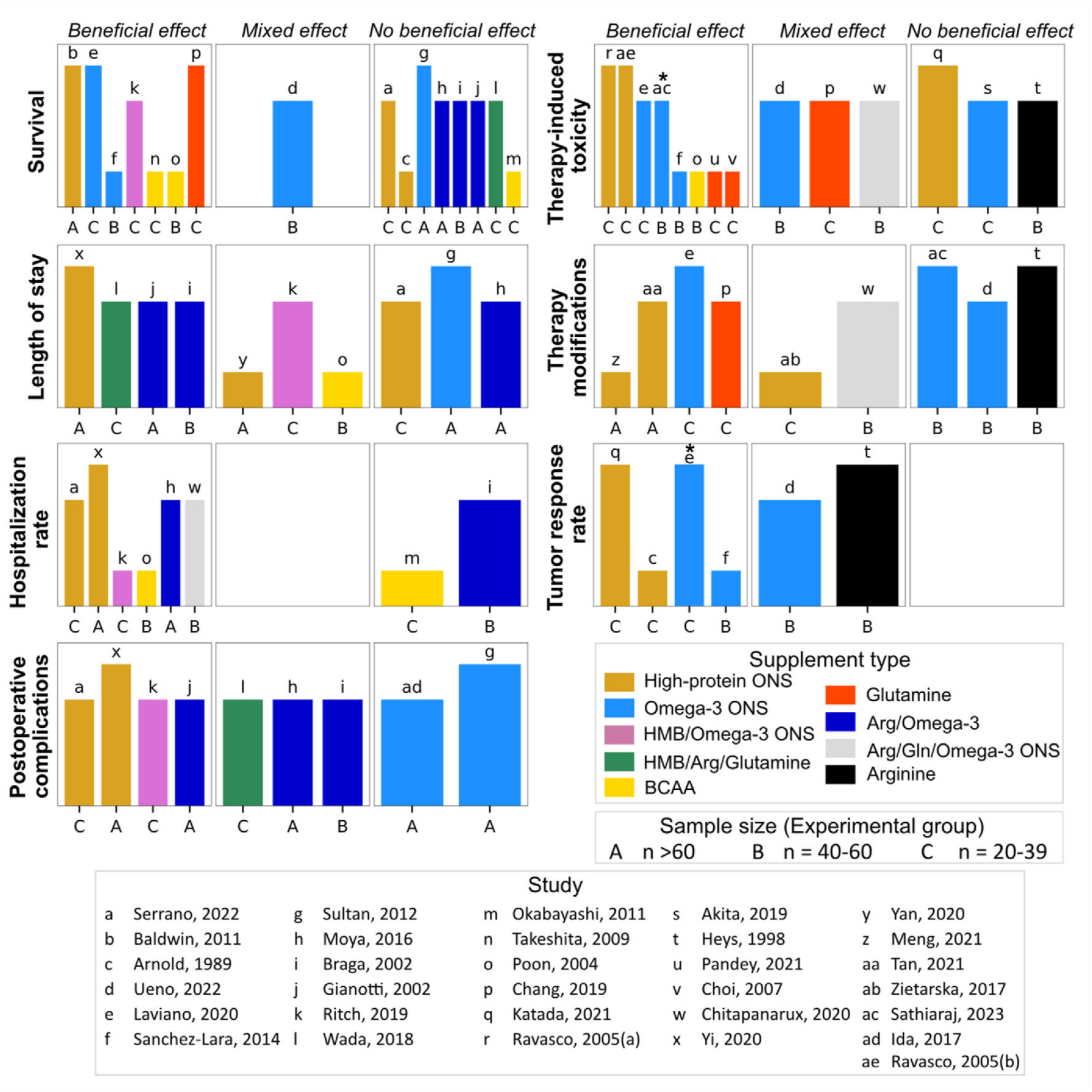
**Harvest plots summarizing the effects ofhigh-proteinsupplementation on survival, length of stay, hospitalization rate, postoperative complications, cancertherapy-inducedtoxicity, therapy modifications, and tumor response rate.** Plots represent the direction of effects based on the differences between experimental and control groups at follow-up, otherwise specified by the symbol (∗) indicating differences in change between experimental and control groups. The height of each bar served as an indicator of study quality, where taller bars signify a lower risk-of-bias, medium-height bars denote a moderate risk-of-bias, and shorter bars signify a high risk-of-bias. Each lowercase letter corresponds to a unique study, while uppercase letters indicate the sample size of experimental groups. Different supplement types are represented by varying colors. Arg, arginine; BCAA, branched-chain amino acids; Gln, glutamine; HMB, β-hydroxy β-methylbutyrate; ONS, oral nutritional supplement.

In 7 of 16 (44%) studies, high-protein supplementation demonstrated a beneficial effect on survival outcomes, including overall survival, progression-free survival, and survival rates compared with controls at follow-up [41,48,49,62,66,68,69] (Figure 5, Supplemental Figure 11). Conversely, 8 of 16 studies reported no beneficial effect, and 1 of 16 found mixed effects regarding the impact of high-protein supplementation on survival. In an analysis stratified by supplement type, findings pertaining to high-protein ONS [49,57,65], omega-3 ONS [39,50,66,69], and BCCA [41,42,48] exhibited inconsistent directions of effects. No beneficial effects were found for Arg/omega-3 supplementation on postoperative survival rates among patients with gastrointestinal cancers in 3 studies [60,61,71]. Furthermore, 1 study reported a longer 30-d survival rate with perioperative HMB/omega-3 ONS supplementation [62]. Another study observed a longer progression-free survival with glutamine supplementation during concurrent chemoradiotherapy [68].

#### Hospitalization

High-protein supplementation conferred no beneficial or mixed effects on postoperative complications in 5 of 9 (56%) studies [37,38,44,50,60,71] and on length of stay in 6 of 10 (60%) [47,48,50,60,62,65], compared with controls at follow-up (Figure 5, Supplemental Figures 12 and 13). In contrast, 6 of 8 (75%) studies reported a beneficial effect of high-protein supplementation on hospital admission rates [48,55,58,60,62,65] (Figure 5, Supplemental Figure 14). Notably, a consistent beneficial effect on postoperative complications and hospitalization rates was demonstrated with high-protein ONS in 2 studies [55,65] and perioperative HMB/omega-3 ONS supplementation in another study [62].

#### Response to cancer therapy/toxicity

High-protein supplementation showed a beneficial effect on cancer therapy-induced toxicity in 8 out of 14 (57%) studies [35,36,48,52,63,64,66,69,70] (Figure 5, Supplemental Figure 15). Nevertheless, results were inconclusive or limited when evaluating the effect based on supplement type. Findings also varied within the context of therapy administration outcomes: 4 of 9 (44%) studies reported a beneficial effect of high-protein ONS [45,46] and omega-3 ONS [66] on chemotherapy modifications, and glutamine supplement on treatment delay [68]; 2 of 9 showed mixed effects of high-protein ONS [54] and Arg/Gln/omega-3 ONS [58]; and 3 of 9 reported no beneficial effect for omega-3 ONS [39,52] and arginine supplementation [51] (Figure 5, Supplemental Figure 16). Additionally, 2 of 2 studies demonstrated a beneficial effect of high-protein ONS on rates of partial and complete response to chemotherapy [40,57] (Figure 5, Supplemental Figure 17).

#### Systemic inflammation

**FIGURE 6:**
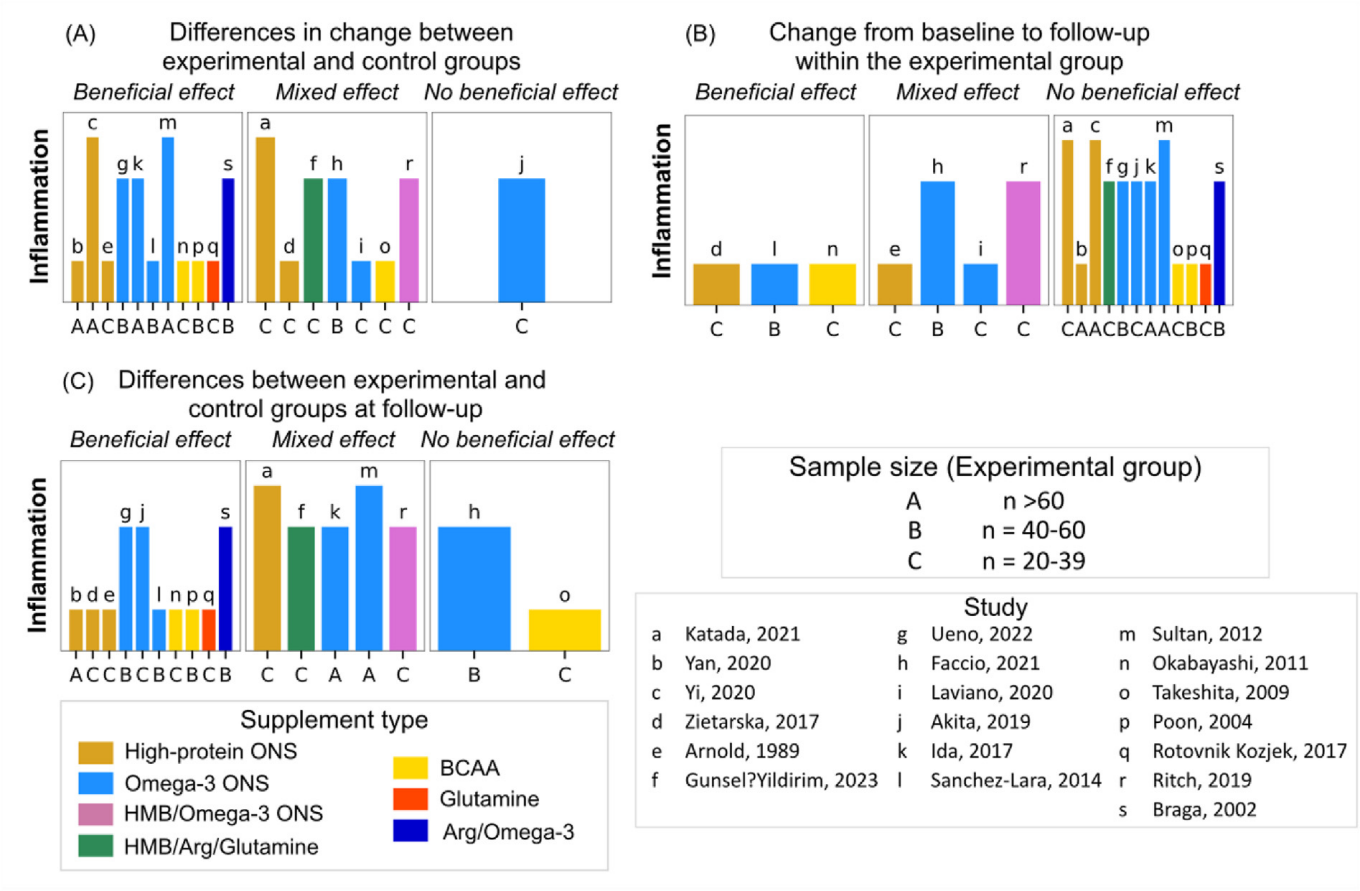
**Harvest plots summarizing the effects of high-protein supplementation on systemic inflammation**. (A) Describes the effects by comparing changes in outcomes between experimental and control groups. (B) Indicates the effects within experimental group considering changes from baseline to follow-up. (C) Represents direction of effects based on the differences between experimental and control groups at follow-up. The height of each bar served as an indicator of study quality, where taller bars signify a lower risk-of-bias, medium-height bars denote a moderate risk-of-bias, and shorter bars signify a high risk-of-bias. Each lowercase letter corresponds to a unique study, while uppercase letters indicate the sample size of experimental groups. Different supplement types are represented by varying colors. Studies examined several markers of systemic inflammation, including albumin, prealbumin, transferrin, white blood cells, lymphocytes, c-reactive protein, IL-6, LPS-binding protein, tumor necrosis factor-alpha, and the neutrophil:lymphocyte ratio. Additionally, systemic inflammation and prognostic nutrition indexes were calculated using inflammatory markers in 1 study [53]. Arg, arginine; BCAA, branched-chain amino acids; HMB, β-hydroxy β-methylbutyrate; ONS, oral nutritional supplement.

Eleven of nineteen (58%) revealed a beneficial effect of high-protein supplementation on systemic inflammation compared with controls, despite variations in cancer types, disease stages, nutritional statuses, and treatment modalities [37,39,42,47,48,50,55,57,67,69,71] (Figure 6, Supplemental Figure 18). Nevertheless, inconsistencies emerged when examining different supplement types, inflammatory markers, and analytical approaches.

#### Safety and tolerability of high-protein supplementation

Sixteen studies evaluated the potential adverse events linked to high-protein supplementation (Supplemental Table 2). Of these, 10 (63%) reported positive safety outcomes, including good tolerance of the high-protein supplementation [42,54,66,68], no occurrence of adverse [67] or serious adverse events [39,55], and comparable rates of adverse outcomes between experimental and control groups [39,54,59,61,69]. Incidence of gastrointestinal adverse events – such as diarrhea, abdominal pain, constipation, and nausea – varied across studies, primarily attributed to differences in supplement types, cancer types, and treatment modalities [51,56,59,61,65,66]. Supplements assessed included omega-3 ONS [56,59,66], high-protein ONS [65], Arg/omega-3 ONS [61], and arginine [51]. Additionally, 2 studies suggested that gastrointestinal symptoms and suboptimal supplement tolerability were associated with low adherence to the prescribed supplement regimen [50,65].

### Potential confounding effects

#### Adherence

Adherence to high-protein supplementation was reported in 23 (66%) studies (Supplemental Table 1) [[37], [38], [39], [40],^43^,^44^,[48], [49], [50],^52^,[54], [55], [56], [57], [58], [59], [60], [61], [62],[65], [66], [67],^69^,^71^]. In 9 studies, ≥80% of the patients adhered to ≥70% of the prescribed high-protein supplementation regimen [44,52,54,55,[60], [61], [62],^65^,^71^]. Conversely, in 8 studies, fewer than 80% of patients had adherence rates below 70% [37,38,43,49,56,57,59,66,69]. Five studies provided information on adherence, but their data could not be categorized into specific groups [39,40,50,58,67]. Lower adherence did not significantly affect the impact of high-protein supplementation on body weight (Supplemental Figure 19). Adherence to the recommended supplementation range was broad, from 53% to 100% [37,38,44,[55], [56], [57],^62^]. Four studies observed a decline in adherence rates over time [37,38,49,60,71]. Additionally, 2 studies reported lower adherence attributed to taste-related issues, specifically in omega-3 ONS supplementation in pancreatic cancer chemoradiotherapy [43] and esophageal or gastric cancer surgery [50].

#### ROB

**FIGURE 7:**
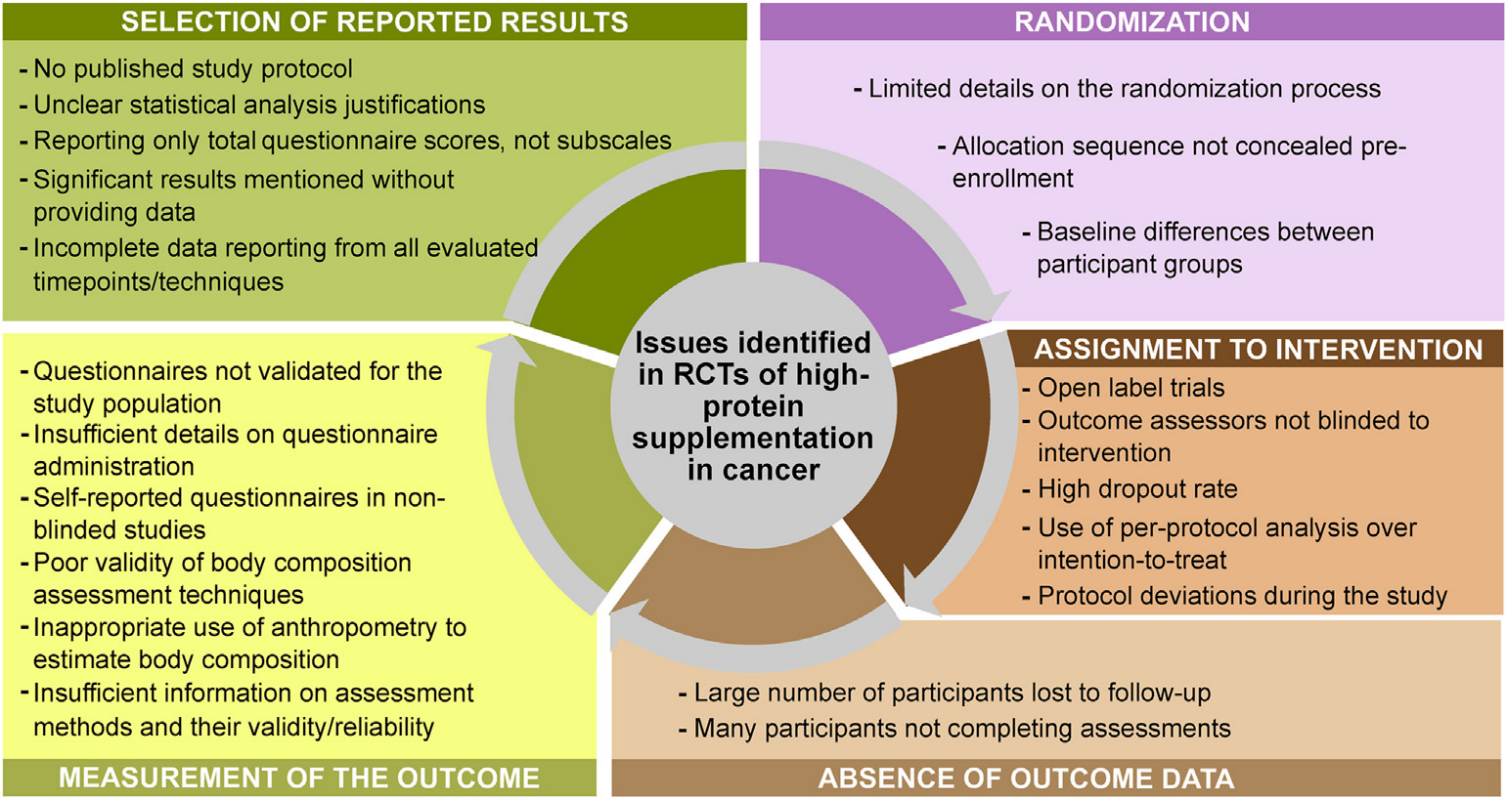
Issues identified in randomized controlled trials (RCTs) of high-protein supplementation in patients with cancer undergoing therapy as part of the risk-of-bias evaluation.

ROB assessment revealed various issues across multiple domains (Figure 7, Supplemental Material 3), including a lack of an intention-to-treat statistical analysis approach and reporting bias. Except for postoperative complications, almost every evaluated outcome had ≥1 study rated as having a high ROB. Outcomes with the highest frequency of high ROB studies were muscle mass (92% of studies), fat mass (83% of studies), physical performance (75% of studies), and HRQoL (60% of studies).

**FIGURE 8:**
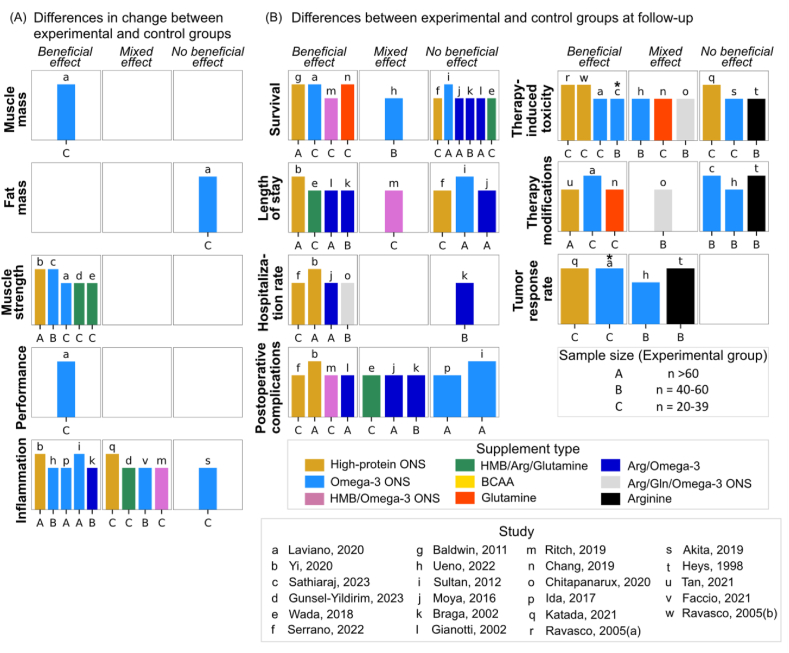
**Evidence overview from higher quality studies on the effects ofhigh-proteinsupplementation on health outcomes of patients with cancer undergoing therapy.** (A) The effects by comparing changes in outcomes between experimental and control groups. (B) The effects based on differences between experimental and control groups at follow-up, otherwise specified by the symbol. Asterisk (∗) indicates differences in change between experimental and control groups. The height of each bar served as an indicator of study quality, where taller bars signify a lower risk-of-bias, and medium-height bars denote a moderate risk-of-bias. Each lowercase letter corresponds to a unique study, while uppercase letters indicate the sample size of experimental groups. Different supplement types are represented by varying colors. Arg, arginine; BCAA, branched-chain amino acids; Gln, glutamine; HMB, β-hydroxy β-methylbutyrate; ONS, oral nutritional supplement.

Sensitivity analysis revealed an effect of high-protein supplementation on body weight in studies with low to intermediate ROB (MD = 1.87 kg; 95% CI: 0.41, 3.34 kg; *P* = 0.01; *I*^*2*^ = 84%) but not in those with high ROB (Supplemental Figure 19). Sensitivity analysis on HRQoL was not possible due to the prevalent high ROB in studies. Among secondary outcomes assessed in ≥3 higher quality studies, improvement in muscle strength was found in all 5 studies, and a decrease in hospitalization rate was observed in 4 of 5 studies (Figure 8). However, the effects of high-protein supplementation on mortality, length of stay, postoperative complications, therapy-induced toxicity, therapy modifications, and tumor response rate varied, indicating a degree of heterogeneity.

## Discussion

This systematic review and meta-analysis examined the effects of high-protein supplementation on patients undergoing cancer therapy. Our findings suggest that high-protein supplementation is safe and leads to less body weight loss, increased handgrip strength, and decreased hospitalization rates (Supplemental Figure 20). These results are primarily supported by studies with low to moderate ROB, ensuring the robustness of these outcomes.

Body weight, often used as an outcome in nutrition research, is a key indicator for diagnosing malnutrition and is clinical/prognostic important for these patients [72,73]. Unintentional weight loss in cancer is associated with postoperative complications, poorer quality of life, and lower overall survival, among other adverse outcomes at all stages of the disease [13,[74], [75], [76]]. Subgroup meta-analyses revealed a significant positive impact of high-protein supplementation on body weight in patients with prior weight loss. However, although this effect was not observed in those at risk of or currently experiencing malnutrition, it remains challenging to isolate weight loss alone from malnutrition. This finding supports the notion that weight gain can be more challenging without prior weight loss [77,78]. It suggests that malnutrition-related factors, such as decreased food intake or absorption, increased disease burden, and inflammation [79,80], can counteract the benefits of high-protein supplementation. Therefore, our findings highlight the potential effectiveness of early and targeted high-protein supplementation in improving treatment outcomes for patients with cancer, especially those who have experienced preintervention weight loss.

HRQoL is an important patient-centered outcome, directly impacted by cancer and its treatment [81]. Although many factors influence HRQoL, previous research links weight loss to lower quality of life [13,82,83]. Managing weight loss with high-protein supplementation may have long-term HRQoL benefits. Although direct HRQoL benefits were not observed, our review consistently showed improved muscle strength with high-protein supplementation. However, evidence for its effects on physical performance was limited.

Our systematic review identified a beneficial effect of high-protein supplementation on muscle mass, a finding supported by biological mechanisms suggesting high protein consumption to promote muscle anabolism by stimulating muscle protein synthesis [79,80]. However, inflammation can inhibit muscle protein synthesis, as reflected in our findings, where 60% of studies showing increased systemic inflammation from baseline to follow-up also reported no gains in muscle mass. Elevated systemic inflammation in cancer accelerates muscle protein breakdown for producing hepatic proteins required in the inflammatory response, reducing responsiveness to nutritional interventions [80,84]. Notably, 2 studies [42,69] using BCAA and omega-3 ONS, known for their anti-inflammatory properties [85,86], reported simultaneous muscle mass increase and systemic inflammation reduction. This concomitant stimulation of muscle protein synthesis and reduction of muscle protein breakdown are ideal for promoting muscle anabolism. As 6 of 7 studies using omega-3 ONS showed a beneficial effect on muscle mass [43,50,52,56,66,69], incorporating immune-modulating nutrients into high-protein supplements may further enhance this process, supporting more effective muscle growth and maintenance. Additionally, high-protein supplementation provides the necessary amino acids for muscle synthesis and contributes to overall energy intake, impacting body weight.

The reviewed supplements contained a minimum of 10 g of protein/serving. Our analysis showed that high-protein supplements, whether in the range of 10−14.9 g or ≥15 g of protein/serving, had a positive effect on body weight. Furthermore, higher daily protein intake (≥40 g) from supplements was more effective than lower doses. Although the precise protein threshold needed to stimulate muscle protein synthesis remains uncertain, our findings suggest that protein requirements likely exceed the minimum recommendation of 1 g/kg/d [17]. This raises the question of whether future guidelines should specify a minimal protein supplement dose per meal or recommend a minimum daily protein intake, a shift from current practice of adjusting total protein intake based on body weight (g/kg/d).

The high-protein supplements reviewed in this study varied, including single and mixed amino acids and ONS. Interestingly, the effects on each outcome differed based on the type of supplement. Concerning ONS, most are recognized for their nutritional completeness and higher energy content. Subgroup meta-analyses revealed that high-protein ONS and omega-3 ONS had beneficial effects on body weight. Additionally, supplements such as BCAA and ONS enriched with HMB and omega-3 also benefited muscle mass. Notably, HMB operates through increased muscle protein synthesis and decreased muscle protein breakdown [87]. Although protein quality plays a role, with essential amino acids and proteins like whey and casein promoting greater muscle protein synthesis [88,89], our analysis was limited due to insufficient information or heterogeneity regarding the protein composition of the supplements. This underscores the importance of understanding supplement composition when planning and prescribing interventions.

Our review found that higher-quality studies suggest that high-protein supplementation reduces hospitalization rates, potentially due to improvements in body weight or muscle mass. Despite the positive impact observed, the overall effects of high-protein supplementation on other outcomes were inconsistent. Contributing factors to this heterogeneity include variations in patient populations, such as cancer type, stage, and nutritional status, as well as differences in supplement composition, including protein content, and methods used for outcome assessment.

High-protein supplementation proves safe for these patients, dispelling concerns that it might fuel tumor growth. These (erroneous) fears, stemming from concerns regarding protein potentially activating the mTOR pathway [17,90] are unfounded, as exercise, widely considered beneficial, also activates mTOR to promote muscle growth [91]. Animal studies showed that protein intake does not affect tumor response to chemotherapy or immune responses [92]. Gastrointestinal events in reviewed studies varied due to cancer, treatments, and supplement types used.

This systematic review assessed the effectiveness of high-protein supplementation with or without nutrition counseling or dietary advice. Although nutrition counseling is a key strategy recommended by nutritional oncology guidelines [18,19], our review excluded studies where high-protein supplementation was provided only after failed nutrition interventions or as a preventive measure in control groups. This exclusion was necessary to avoid issues like inconsistent supplement distribution among participants or cross-contamination. Another limitation was the inclusion of supplements containing additional nutrients or ingredients, such as HMB and/or omega-3, alongside protein. Although these components could not be separated from the effects of protein, they exist in certain commercially available supplements and may provide added benefits. Our results may differ from previous reviews focusing on these specific nutrients/ingredients among patients with cancer [87,93] due to our distinct eligibility criteria and a smaller number of included studies on these supplements. Additionally, our review could not determine if changes in body weight were due to alterations in fat mass and/or muscle mass. Limited higher-quality studies, particularly those addressing HRQoL and secondary outcomes, such as muscle mass, also pose limitations that require cautious interpretation of findings. The high ROB in these outcomes not only constrained our analysis but also underscored the need for higher quality clinical nutrition studies. Researchers should provide clear methodological descriptions, particularly when evaluating body composition, to enhance study quality and advance the field. Additionally, the high variability in body composition techniques presented a challenge in comparing findings. Quality of life, often assessed by questionnaires, also requires more clarity, as it was often unclear whether these assessments were self-reported or administered by researchers, making evaluation difficult. Furthermore, the included studies were not sufficiently powered to detect outcome changes, which limited our analysis.

Finally, integrating these findings into clinical practice is feasible considering the importance of early and continuing nutrition therapy during cancer treatment for optimal benefits. Healthcare professionals should consider using high-protein supplements, especially for patients experiencing weight loss. Regularly monitoring patient responses to supplementation is crucial to assess its effectiveness. Additionally, it is important to recognize that while high-protein supplementation can have a positive impact on specific health outcomes, its full effectiveness may be limited without adequate energy and nutrient intakes.

## Author contributions

The authors’ responsibilities were as follows – CEO, JA, MAEdvdS, NK, AL, and CMP: designed the research; CEO: searched the literature; CEO, AC, and TSP: evaluated the quality of the data and composed the tables; CEO: analyzed data; and all authors: contributed to the interpretation of the findings, contributed to writing the final article, and read and approved the final manuscript.

## Funding

The work of CMP was partially supported by the Canada Research Chairs Program and the Campus Alberta Innovation Program.

## Data availability

Data described in the manuscript, code book, and analytic code will be made available upon request pending approval from the corresponding author.

## Conflict of interest

CEO has received honoraria from Abbott Nutrition. JA reports receiving speaking and lecture fees from Baxter and Nutricia. AL reports receiving honoraria and/or paid consultancy from Abbott, Baxter, B. Braun, Fresenius Kabi, Nestlé Health Science, Nutricia, and Smartfish, and research grant from Fresenius Kabi. CMP has previously received honoraria and/or paid consultancy from Abbott Nutrition, Nutricia, Almased, Nestlé Health Science, Pfizer, and AMRA Medical. NK reports speaking and lecture fees and paid consultancy from Abbott Nutrition. The other authors report no conflicts of interest.
