# Supplementary material for: Effects of high-protein supplementation during cancer therapy: a systematic review and meta-analysis
Source: Am J Clin Nutr. 2024 Dec 2;120(6):1311–24. doi: 10.1016/j.ajcnut.2024.08.016 (PMC11619795; doi:10.1016/j.ajcnut.2024.08.016)
Supplement: multimedia component 1 [file mmc1.docx]

**Appendix A: Supplementary data**

[Supplemental Material 1. Eligibility criteria.](#_Toc176864879)

[Supplemental Material 2. Search strategy.](#_Toc176864880)

[Supplemental Material 3. Risk-of-bias assessment of included studies, stratified by study, outcomes, and domains.](#_Toc176864881)

[Supplemental Table 1. Characteristics of the included studies.](#_Toc176864882)

[Supplemental Table 2. Adverse events reported in the included studies.](#_Toc176864883)

[Supplemental Figure 1. Summary of clinical characteristics of patients with cancer included in the systematic review and meta-analysis.](#_Toc176864884)

[Supplemental Figure 2. Summary of characteristics of high-protein supplements.](#_Toc176864885)

[Supplemental Figure 3. Meta-analysis of the effects of high-protein supplementation on body weight in kilograms, percentage change, or BMI.](#_Toc176864886)

[Supplemental Figure 4. Subgroup meta-analyses of the effects of high-protein supplementation on body weight (in kg) based on total daily protein intake from high-protein supplement and based on the duration of high-protein supplementation](#_Toc176864887)

[Supplemental Figure 5. Subgroup meta-analyses of the effects of high-protein supplementation on body weight (in kg) based on cancer and treatment types.](#_Toc176864888)

[Supplemental Figure 6. Subgroup meta-analyses of the effects of high-protein supplementation on body weight (in kg) based on pre-intervention nutrition status and changes in inflammation.](#_Toc176864889)

[Supplemental Figure 7. Overview of the studies assessing the effects of high-protein supplementation on body composition.](#_Toc176864890)

[Supplemental Figure 8. Changes in muscle-related measures from baseline to follow-up within high-protein supplementation (green bar) and control (blue bar) groups.](#_Toc176864891)

[Supplemental Figure 9. Changes in fat mass (and related measures) from baseline to follow-up within high-protein supplementation (green bar) and control (blue bar) groups.](#_Toc176864892)

[Supplemental Figure 10. Changes in measures of muscle function and performance from baseline to follow-up within high-protein supplementation (green bar) and control (blue bar) groups.](#_Toc176864893)

[Supplemental Figure 11. Survival outcomes at follow-up within high-protein supplementation (green bar) and control (blue bar) groups.](#_Toc176864894)

[Supplemental Figure 12. Complications at follow-up within high-protein supplementation (green bar) and control (blue bar) groups.](#_Toc176864895)

[Supplemental Figure 13. Length of stay at follow-up within high-protein supplementation (green bar) and control (blue bar) groups.](#_Toc176864896)

[Supplemental Figure 14. Hospital admission or readmission rates and unplanned emergency room visits at follow-up within high-protein supplementation (green bar) and control (blue bar) groups.](#_Toc176864897)

[Supplemental Figure 15. Incidence of cancer therapy-induced toxicities at follow-up within high-protein supplementation (green bar) and control (blue bar) groups.](#_Toc176864898)

[Supplemental Figure 16. Cancer therapy modifications within high-protein supplementation (green bar) and control (blue bar) groups.](#_Toc176864899)

[Supplemental Figure 17. Tumor response at follow-up within high-protein supplementation (green bar) and control (blue bar) groups.](#_Toc176864900)

[Supplemental Figure 18. Changes in inflammation markers from baseline to follow-up within high-protein supplementation (green bar) and control (blue bar) groups.](#_Toc176864901)

[Supplemental Figure 19. Subgroup meta-analyses of the effects of high-protein supplementation on body weight (in kg) based on (A) adherence to prescribed high-protein supplementation regimen, and (B) risk-of-bias of included studies.](#_Toc176864902)

[Supplemental Figure 20. Graphical abstract illustrating the overall findings of this systematic review on the effects of high-protein supplementation during cancer therapy. The number of studies showing a beneficial effect on each outcome is depicted in colored boxes, stratified by different supplement types and relative to the total number of studies analyzed per supplement. The remaining percentages in each box represent studies with mixed findings or no beneficial effect. Dashed boxes represent all supplements combined. Panel A shows all studies analyzed regardless of study quality, while Panel B shows only higher-quality studies (i.e., those with low to moderate risk of bias).](#_Toc176864903)

[References](#_Toc176864904)

## Supplemental Material 1. Eligibility criteria.

|  | **Inclusion criteria** | **Exclusion criteria** |
| --- | --- | --- |
| **Participants/**  **population** | Patients with cancer:   - adults (≥18 years) - of any nutritional status - undergoing cancer treatment (i.e., radiotherapy, chemotherapy, surgery, immunotherapy, hormone therapy, and targeted therapy). | - Healthy adults (≥18 years), pregnancy and lactation. - Adults diagnosed with diseases other than cancer. - Children and adolescents (<18 years) with or without cancer. - Animal studies. |
| **Intervention** | Oral nutritional supplements (ONS) or protein supplementation (i.e., provided as a single amino-acid or as an amino acid mixture)   - containing at least 10 g of protein per serving of the supplement - provided in any form (e.g., powder, ready to drink liquids, crèmes, bars) - studies of any duration - supplements may be enriched with anabolic agents (e.g., n-3 long-chain polyunsaturated fatty acids [n-3 PUFAs], leucine, β-hydroxy β-methylbutyrate [HMB], glutamine, carnitine, creatine, arginine, ribonucleic acid [RNA], vitamin D, Adenosine-5′-Triphosphate) - patients may be receiving dietary counseling concurrently. - in patients undergoing surgery for cancer, protein supplementation must be applied both preoperatively and postoperatively. | - Dietary counselling alone. - Enteral tube feeding alone. - Parenteral nutrition. - ONS with <10 g of protein per serving. - Protein supplement with <10 g of protein per serving. - Dietary and exercise interventions combined. - Other nutrition interventions not focusing on increasing protein intake. - Preoperative protein supplementation alone in patients undergoing surgery for cancer. |
| **Comparator** | Placebo or standard of care   - no nutritional intervention - nutrition counseling alone | Not applicable |
| **Outcomes** | Total energy intake   - assessed by subjective methods (e.g., 24-hour dietary recall, dietary record, food frequency questionnaire), objective observation (e.g., intake recorded by a research or health care staff), or doubly labeled water.   Total protein intake   - measured by subjective methods (e.g., 24-hour dietary recall, dietary record, food frequency questionnaire), objective observation (e.g., intake recorded by a research or health care staff), or biomarkers (e.g., nitrogen in 24-hour urine).   Body weight or body mass index  Fat mass, fat-free mass, lean soft tissue, or skeletal muscle   - assessed by any anthropometric or body composition technique.   Muscular strength   - measured using isometric or isokinetic testing.   Physical performance   - evaluated by the short physical performance battery, gait speed, timed up and go, chair raise, balance tests, step count, or cardiorespiratory capacity.   Health-related quality of life   - using validated instruments (e.g., Short Form 36 [SF-36], European Organization for Research and Treatment of Cancer Quality of Life Questionnaire Core 30 [EORTC QLQ-C30], EuroQol 5-Dimension [EQ-5D].   Survival   - (any type of death or survival rate measures presented as unadjusted or adjusted ratio data).   Cancer therapy-induced toxicity   - measured using any validated toxicity grading system.   Tumor response   - evaluated by any validated tool, such as the Response Evaluation Criteria in Solid Tumors (RECIST).   Changes in scheduling of anti-cancer treatments.  Hospitalization rate, length of hospital stay, incidence of postoperative complications. | Not applicable |
| **Study design** | Randomized controlled trials   - with at least 20 participants in each study arm. | - Nonrandomized controlled trials. - Reports not including original data (e.g., meta-analyses, systematic reviews, study protocols, or case series). |
| **Others** | - Full text article - English Language. | - Language other than English. - Conference proceeding. - Abstract only. |

## Supplemental Material 2. Search strategy.

**MEDLINE (via OVID)**

**Search date:** First searched on Feb 12, 2021; updates on Oct 20, 2021; July 27, 2022; July 05, 2023.

**Search results:** 2856 on Feb 12, 2021; 3068 on Oct 20, 2021; 3540 on July 27, 2022; 3467 on July 05, 2023.

| No. | Search term |
| --- | --- |
| 1 | oral nutrition* supplement*.mp. |
| 2 | (protein* adj2 ONS).mp. |
| 3 | oral protein*.mp. |
| 4 | (protein* adj3 diet*).mp. |
| 5 | exp Diet, High-Protein/ |
| 6 | high-protein*.mp. |
| 7 | exp Dietary Proteins/ |
| 8 | (protein* adj3 supplement*).mp. |
| 9 | (protein* adj3 intake*).mp. |
| 10 | (protein* adj3 consum*).mp. |
| 11 | (protein* adj3 isolate*).mp. |
| 12 | (nutrition* adj3 protein*).mp. |
| 13 | soy protein*.mp. |
| 14 | exp Whey Proteins/ |
| 15 | (milk adj2 protein*).mp. |
| 16 | casein.mp. |
| 17 | Amino Acids, Branched-Chain/ |
| 18 | Amino Acids, Essential/ |
| 19 | diet* amino acid*.mp. |
| 20 | (leucine adj3 supplement*).mp. |
| 21 | (albumin adj3 supplement*).mp. |
| 22 | branched-chain amino acid*.mp. |
| 23 | (arginine adj3 supplement*).mp. |
| 24 | HMB.mp. |
| 25 | beta-hydroxy beta-methylbutyrate.mp. |
| 26 | Dietary Supplements/ |
| 27 | (diet* adj3 supplement*).mp. |
| 28 | Nutritional Support/ |
| 29 | (nutrition* adj3 supplement*).mp. |
| 30 | sip feed*.mp. |
| 31 | or/1-30 |
| 32 | exp Neoplasms/ |
| 33 | neoplasm*.mp. |
| 34 | cancer*.mp. |
| 35 | exp Carcinoma/ |
| 36 | oncolog*.mp. |
| 37 | cancer* cell*.mp. |
| 38 | malignan*.mp. |
| 39 | or/32-38 |
| 40 | randomized controlled trial.pt. |
| 41 | controlled clinical trial.pt. |
| 42 | randomized.ab. |
| 43 | placebo.ab. |
| 44 | randomly.ab. |
| 45 | trial.ab. |
| 46 | groups.ab. |
| 47 | or/40-46 |
| 48 | exp animals/ not humans/ |
| 49 | 47 not 48 |
| 50 | 31 and 39 and 49 |
| 51 | limit 50 to english language |
| 52 | limit 51 to ("all infant (birth to 23 months)" or "all child (0 to 18 years)") |
| 53 | limit 51 to "all adult (19 plus years)" |
| 54 | 51 not 52 |
| 55 | 53 or 54 |

**CINAHL**

**Search date:** First searched on February 12, 2021; updates on Oct 20, 2021; July 27, 2022; July 05, 2023.

**Search results:** 1200 on Feb 12, 2021; 1333 on Oct 20, 2021; 3126 on July 27, 2022; 1564 on July 05, 2023.

| No. | Search term |
| --- | --- |
| S1 | "oral nutrition* supplement*" OR (protein*) N3 (ONS OR diet* OR supplement* OR intake* OR consum* OR isolate* OR nutrition*) OR "oral protein*" OR MH Dietary proteins OR "high-protein*" OR MH Protein intake |
| S2 | "soy* protein*" OR "whey protein*" OR (milk) N2 (protein*) OR casein OR "branched chain amino acid*" OR "essential amino acid*" OR "diet* amino acid*" OR (supplement*) N3 (leucine OR albumin OR arginine) OR HMB OR "Beta hydroxy beta methylbutyrate" |
| S3 | (diet*) N3 (supplement*) OR (nutrition*) N3 (support* OR supplement*) OR "sip feed*" |
| S4 | S1 OR S2 OR S3 |
| S5 | MH neoplasms OR neoplasm* OR cancer* OR MH carcinoma OR MH oncology OR "cancer* cell*" OR malignan |
| S6 | ( MH ( randomized controlled trials OR double‐blind studies OR single‐blind studies OR random assignment OR pretest‐posttest design OR cluster sample ) OR TI ( randomised OR randomized ) OR AB random* OR TI trial OR ( (MH (sample size) AND AB (assigned OR allocated OR control)) ) OR MH ( placebos OR crossover design OR comparative studies ) OR AB ( (control W5 group) OR (cluster W3 RCT) OR PT (randomized controlled trial)) ) NOT ( ( MH animals+ OR MH (animal studies) OR TI (animal model*) ) NOT MH (human) ) |
| S7 | S4 AND S5 AND S6 |

**Embase (via Elsevier)**

**Search date:** First searched on Feb 12, 2021; updates on Oct 20, 2021; July 27, 2022; July 05, 2023.

**Search results:** 2872 on Feb 12, 2021; 3070 on Oct 20, 2021; 4550 on July 27, 2022; 4550 on July 05, 2023.

| No. | Search term |
| --- | --- |
| #1 | 'oral nutrition* supplement*':ab,ti,kw OR ((protein* NEAR/2 ons):ab,ti,kw) OR 'oral protein*':ab,ti,kw OR ((protein* NEAR/3 diet*):ab,ti,kw) OR 'protein diet'/exp OR 'high-protein*':ab,ti,kw OR ((protein* NEAR/3 supplement*):ab,ti,kw) OR ((protein* NEAR/3 intake*):ab,ti,kw) OR 'protein intake'/exp OR ((protein* NEAR/3 consum*):ab,ti,kw) OR ((protein* NEAR/3 isolate*):ab,ti,kw) OR ((nutrition* NEAR/3 protein*):ab,ti,kw) OR 'soy* protein*':ab,ti,kw OR 'soybean protein'/exp OR 'whey protein'/exp OR 'whey protein*':ab,ti,kw OR ((milk NEAR/2 protein*):ab,ti,kw) OR casein:ab,ti,kw OR 'casein'/exp OR 'branched chain amino acid'/de OR 'branched chain amino acid*':ab,ti,kw OR 'essential amino acid'/de OR 'essential amino acid*':ab,ti,kw OR 'diet* amino acid*':ab,ti,kw OR ((leucine NEAR/3 supplement*):ab,ti,kw) OR ((albumin NEAR/3 supplement*):ab,ti,kw) OR ((arginine NEAR/3 supplement*):ab,ti,kw) OR hmb:ab,ti,kw OR 'beta hydroxy beta methylbutyrate':ab,ti,kw OR 'dietary supplement'/de OR ((diet* NEAR/3 supplement*):ab,ti,kw) OR 'nutritional support'/de OR ((nutrition* NEAR/3 support*):ab,ti,kw) OR 'nutrition supplement'/de OR ((nutrition* NEAR/3 supplement*):ab,ti,kw) OR 'sip feed*':ab,ti,kw |
| #2 | 'neoplasm':de,ab,ti,kw OR neoplasm*:ab,ti,kw OR 'malignant neoplasm':de,ab,ti,kw OR cancer*:ab,ti,kw OR 'carcinoma':de,ab,ti,kw OR carcinoma:ab,ti,kw OR 'oncology':de,ab,ti,kw OR oncolog*:ab,ti,kw OR 'cancer cell':de,ab,ti,kw OR 'cancer* cell*':ab,ti,kw |
| #3 | 'crossover procedure':de OR 'double-blind procedure':de OR 'randomized controlled trial':de OR 'single-blind procedure':de OR random*:de,ab,ti OR factorial*:de,ab,ti OR crossover*:de,ab,ti OR ((cross NEXT/1 over*):de,ab,ti) OR placebo*:de,ab,ti OR ((doubl* NEAR/1 blind*):de,ab,ti) OR ((singl* NEAR/1 blind*):de,ab,ti) OR assign*:de,ab,ti OR allocat*:de,ab,ti OR volunteer*:de,ab,ti |
| #4 | #1 AND #2 AND #3 |
| #5 | 'animal'/exp NOT 'human'/exp |
| #6 | #4 NOT #5 |

**Cochrane CENTRAL**

**Search date:** First searched on Feb 12, 2021; updates on Oct 20, 2021; July 27, 2022; July 05, 2023.

**Search results:** 1393 on Feb 12, 2021; 1454 on Oct 20, 2021; 1559 on July 27, 2022; 1781 on July 05, 2023.

| No. | Search term |
| --- | --- |
| #1 | (“oral nutrition* supplement*” OR ( protein near/2 ( ons OR oral OR diet* OR high OR supplement* OR intake* OR consum* OR isolate* OR nutrition* OR soy OR whey OR milk ) ) OR casein OR “branched chain amino acid*” OR “essential amino acid*” OR “dietary amino acid*” OR ( supplement* near/2 ( leucine OR albumin OR arginine OR hmb OR “beta hydroxy beta methylbutyrate” OR diet* OR nutrition* ) ) OR “sip feed*”):ti,ab,kw AND (neoplasm* OR "malignant neoplasm" OR cancer* carcinoma OR oncolog* OR "cancer* cell*"):ti,ab,kw (Word variations have been searched) in Trials |

**SCOPUS**

**Search date:** First searched on Feb 12, 2021; updates on Oct 20, 2021; July 27, 2022; July 05, 2023.

**Search results:** 4240 on Feb 12, 2021; 4474 on Oct 20, 2021; 4535 on July 27, 2022; 4785 on July 05, 2023.

| No. | Search term |
| --- | --- |
| #1 | ( ( TITLE-ABS-KEY ( {Clinical-trial} OR {controlled-trial} OR randomi* OR randomly OR ( random W/4 ( allocat* OR distribut* OR assign* ) ) OR {placebo} OR {trial} OR {groups} OR {subgroups} ) OR TITLE ( rct ) ) AND ( TITLE-ABS-KEY ( neoplasm* OR {malignant neoplasm} OR cancer* OR carcinoma OR oncolog* ) ) AND ( ( TITLE-ABS-KEY ( {oral nutrition supplement} OR {oral nutritional supplement} OR {oral nutrition supplementation} OR {oral nutritional supplementation} ) ) OR ( TITLE-ABS-KEY ( {protein ons} OR {ons protein} OR {oral protein} OR {dietary protein} OR {protein diet} OR {high protein} OR {protein supplement} OR {protein supplementation} OR {supplementing with protein} OR {protein intake} OR {protein consumption} OR {protein isolate} OR {isolated protein} OR {nutritional protein} OR {soy protein} OR {soybean protein} OR {whey protein} OR {milk protein} ) ) OR ( TITLE-ABS-KEY ( {casein} OR {branched chain amino acids} OR { essential amino acids} OR {dietary amino acid} ) ) OR ( TITLE-ABS-KEY ( {sip feed} OR {sip feeding} ) ) OR ( TITLE-ABS-KEY ( {leucine supplement} OR {albumin supplement} OR {arginine supplement} OR {hbm supplement} ) ) OR ( TITLE-ABS-KEY ( {diet supplement} OR {diet supplementation} OR {dietary supplement} OR {dietary supplementation} OR {diet supplementation} OR {supplemental diet} OR {nutrition supplement} OR {nutrition supplementation} OR {nutritional supplement} OR {nutritional supplementation} OR {supplemental nutrition} ) ) ) ) AND NOT ( ( animals ) AND NOT ( humans ) ) AND ORIG-LOAD-DATE AFT 20210209 AND ( LIMIT-TO ( DOCTYPE , "ar" ) ) AND ( LIMIT-TO ( LANGUAGE , "English" ) ) |

**Grey literature**

**Search date:** First searched on Feb 12, 2021; updates on Oct 20, 2021; July 27, 2022; July 05, 2023.

| **Source Searched** | **Date searched** | **Search terms** | **Results** |
| --- | --- | --- | --- |
| Clinicaltrials.gov | Feb 12, 2021  At 20:40  Oct 20, 2021  At 10:37  July 27, 2022  At 15:07  July 05, 2023  At 19:02 | Condition or disease: neoplasm OR "malignant neoplasm" OR cancer OR carcinoma OR oncology  Other terms: "oral nutritional supplement" OR "protein ONS" OR "dietary protein" OR "protein supplement" OR "protein diet" OR "protein isolate" OR "nutritional protein" OR "soy protein" OR "whey protein" OR "milk protein" OR "amino acid" OR "dietary supplement"  Filters: Adult, older  Completed, with results, interventional  Adult (18-64), older adult (65+) | 140 on Feb 12, 2021  154 on Oct 20, 2021  166 on July 27, 2022  27 on July 05, 2023 |
| Google | Last search July 05, 2023  At 20:07 | "cancer" AND oral-nutritional-supplement OR protein supplement OR soy protein OR whey protein AND -pubmed -wiley -sciencedirect -review | First 100 results reviewed |

Time is in Brasilia time.

## Supplemental Material 3. Risk-of-bias assessment of included studies, stratified by study, outcomes, and domains.

### Quality assessment – Body weight


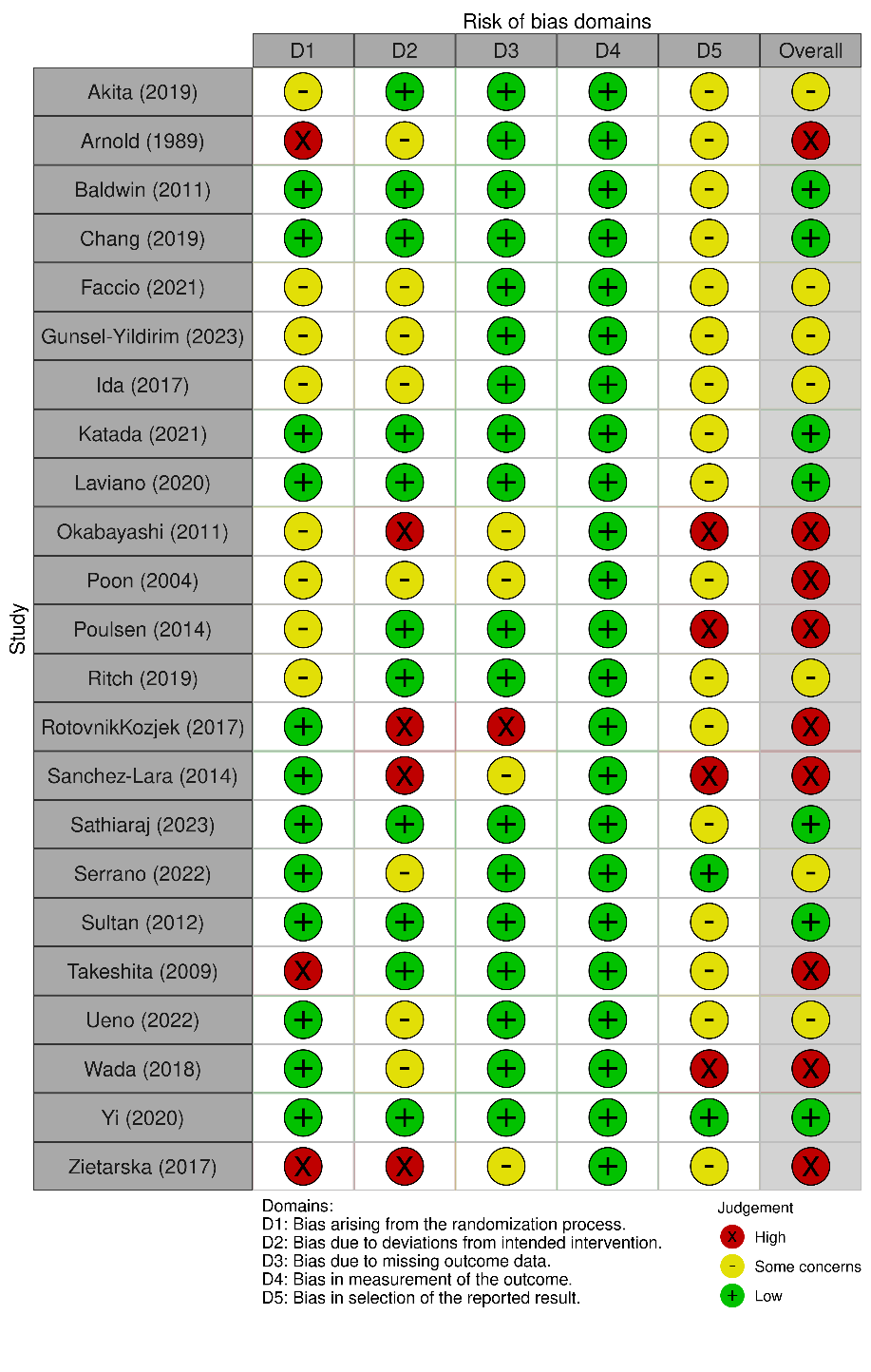


### Quality assessment – Quality of life


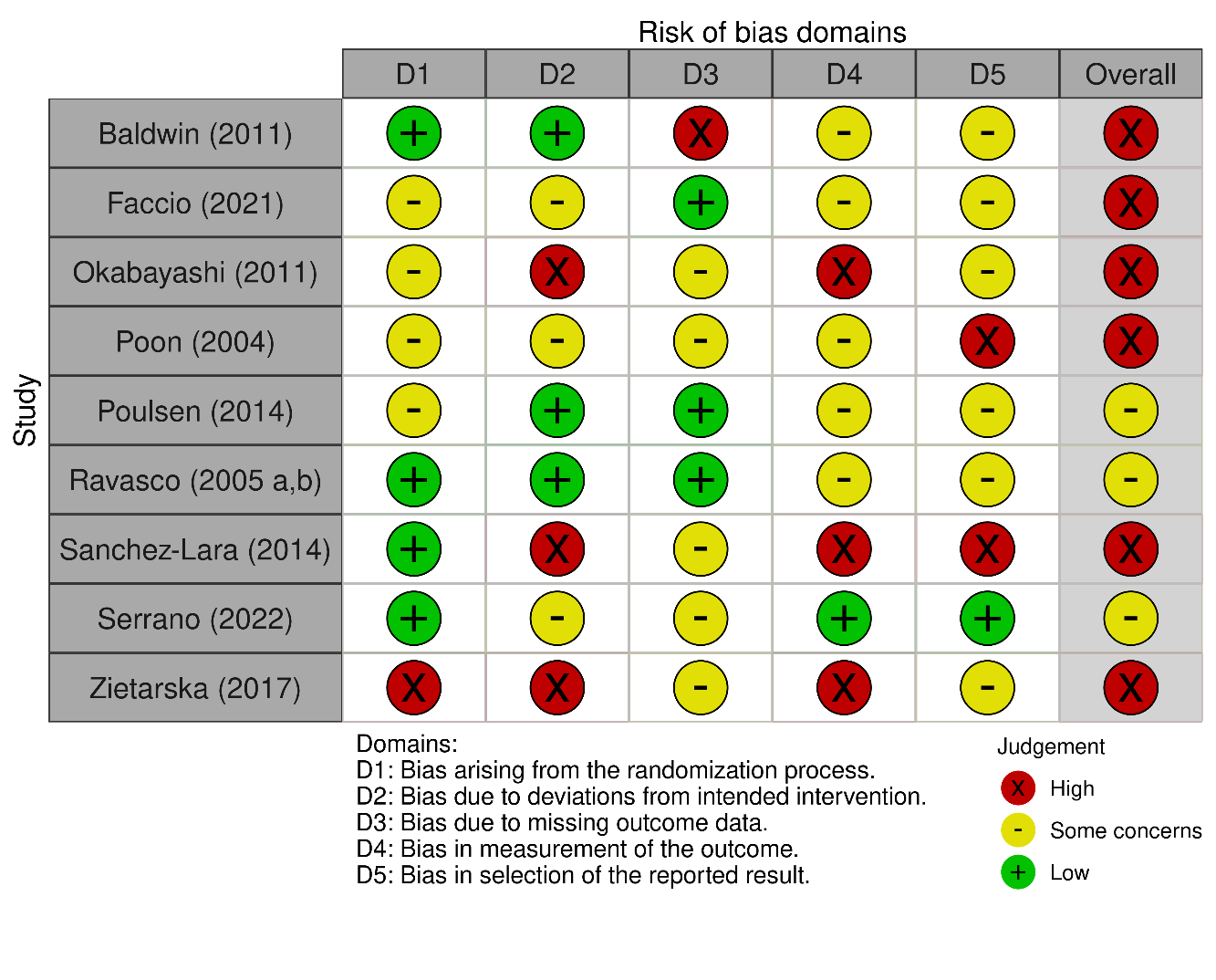


### Quality assessment – Muscle mass


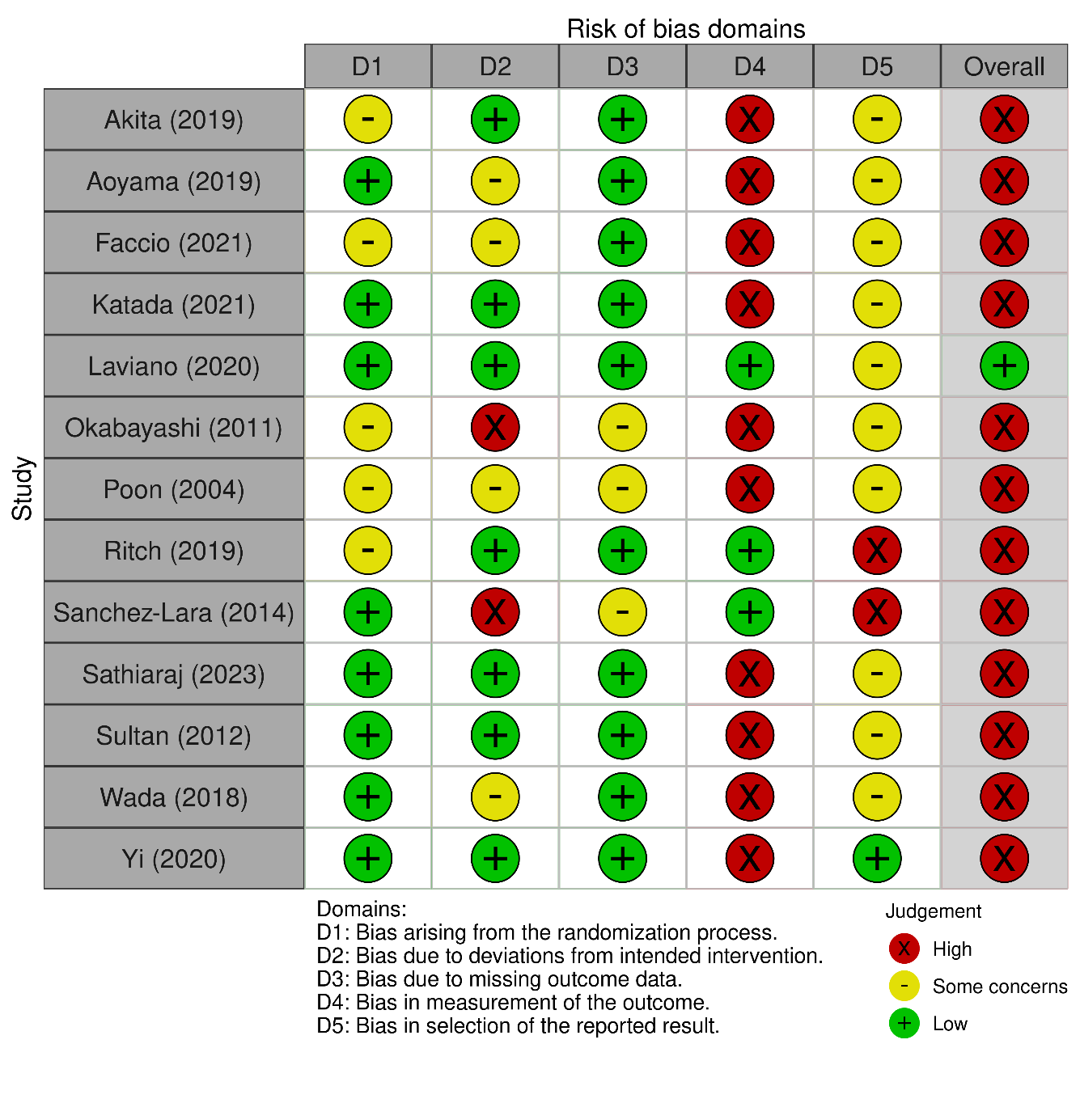


### Quality assessment – Fat mass


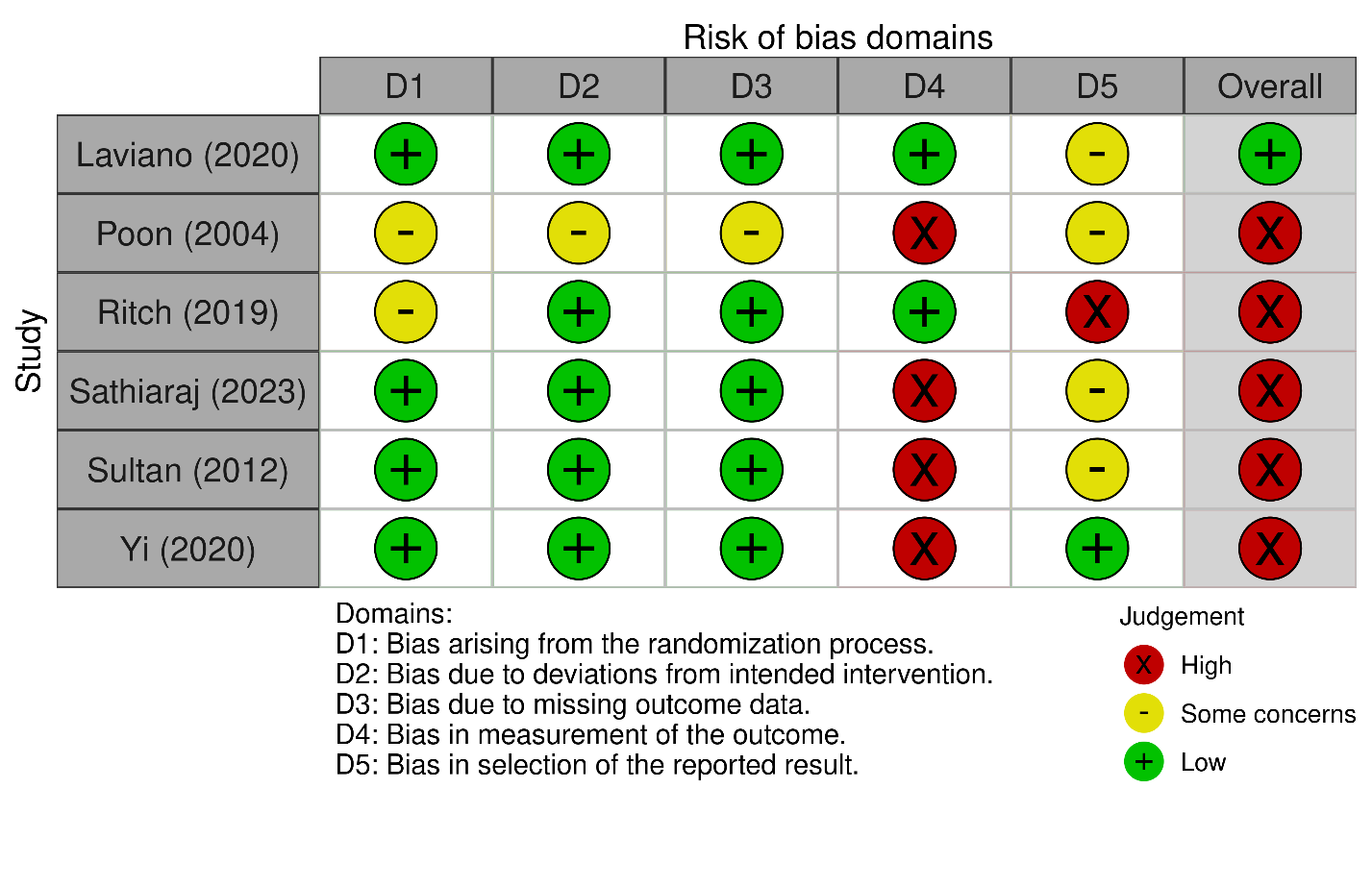


### Quality assessment – Muscle strength


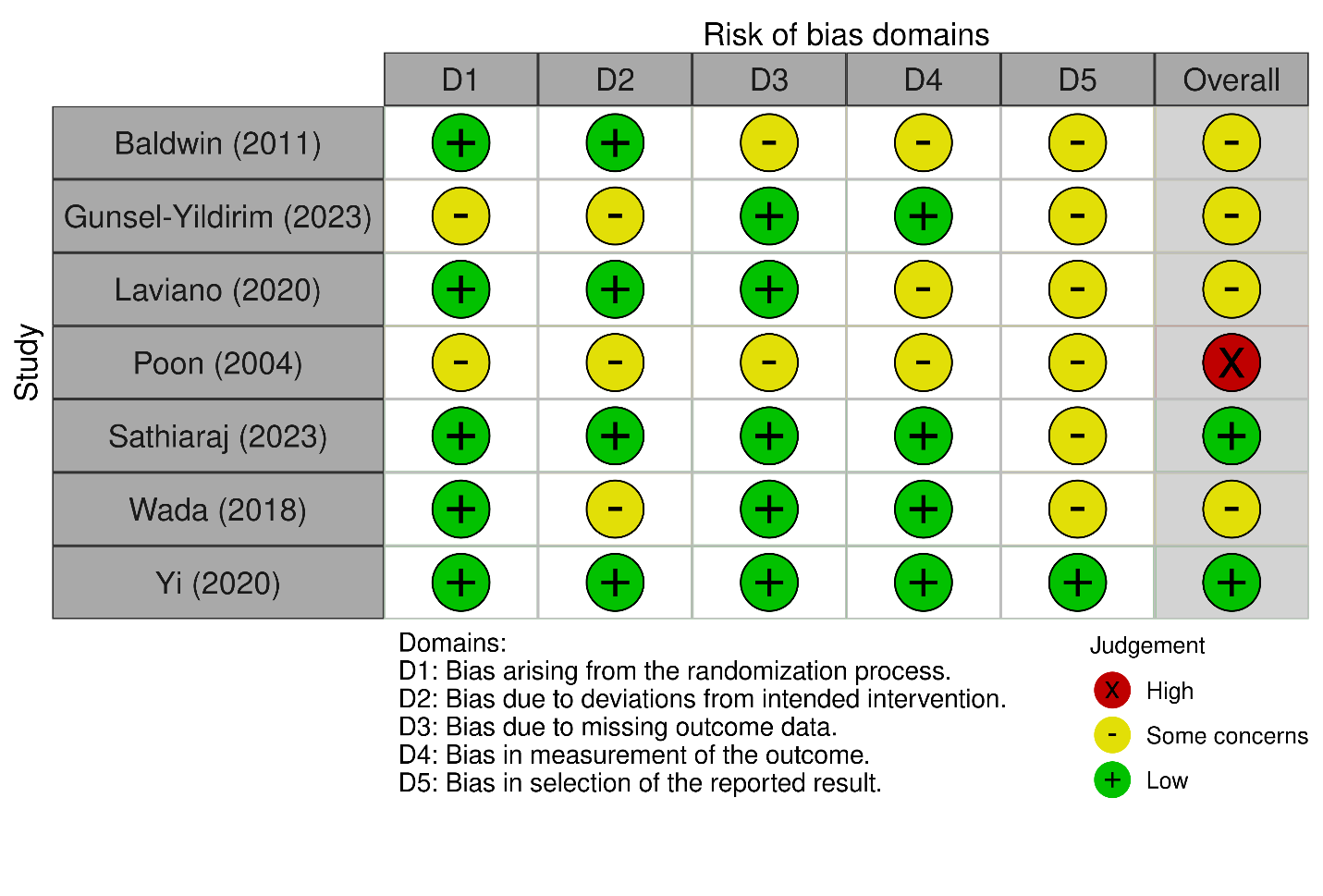


### Quality assessment – Physical performance


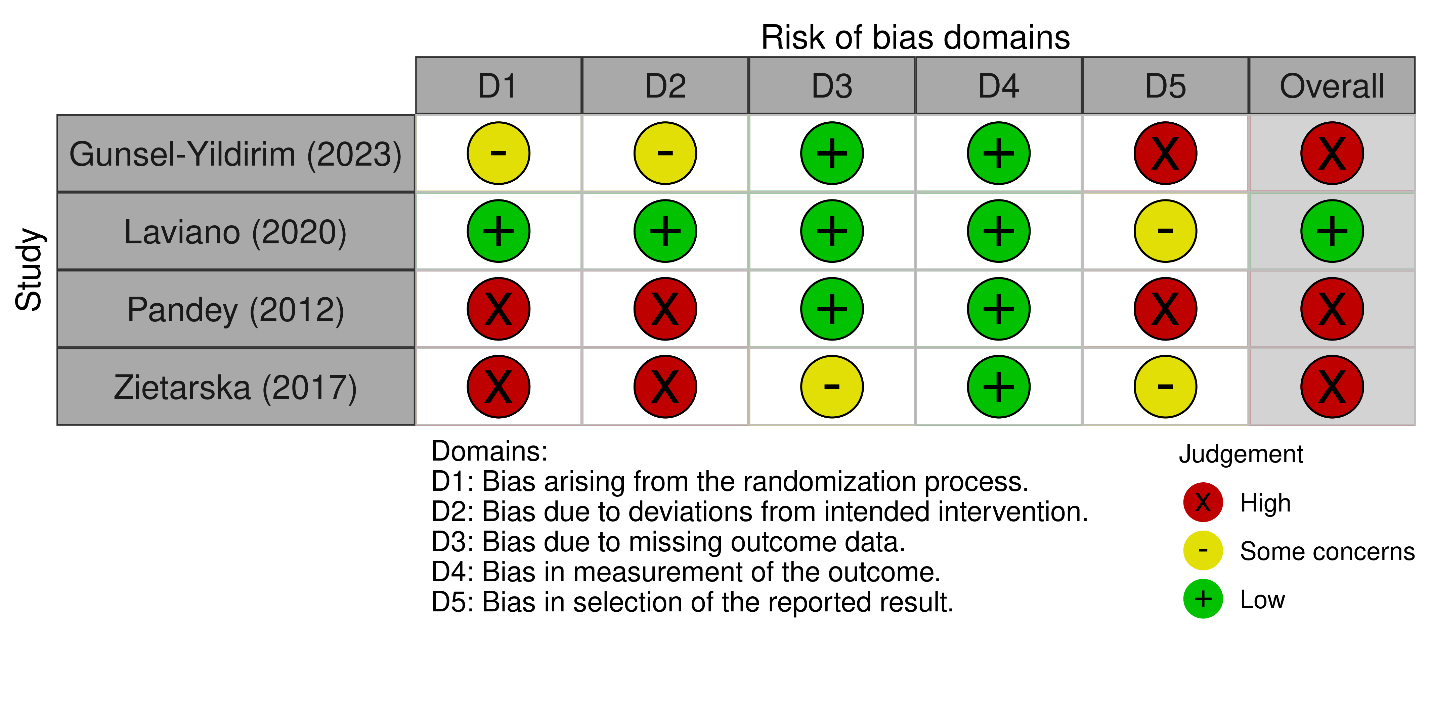


### Quality assessment – Survival


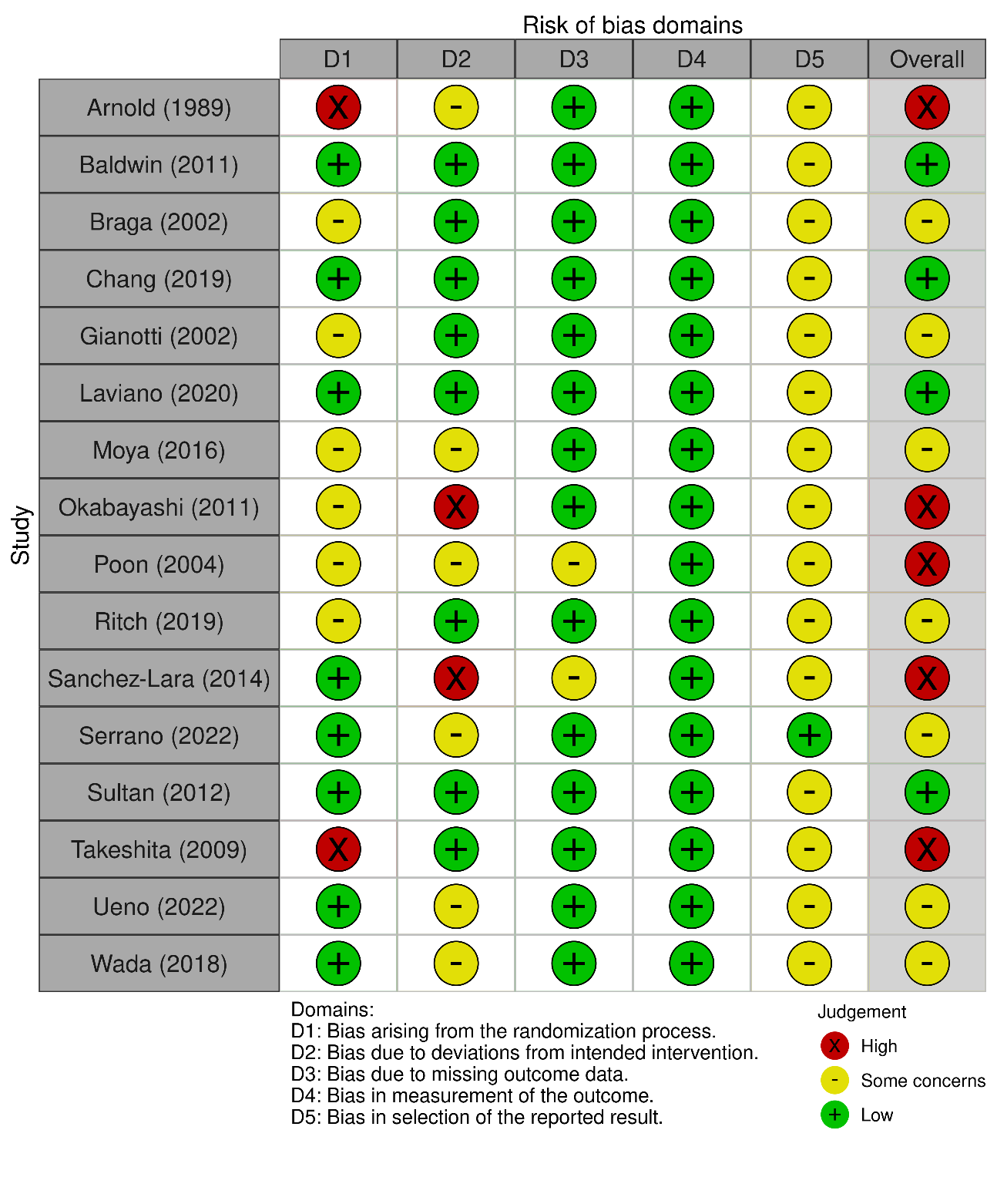


### Quality assessment – Hospitalization rate


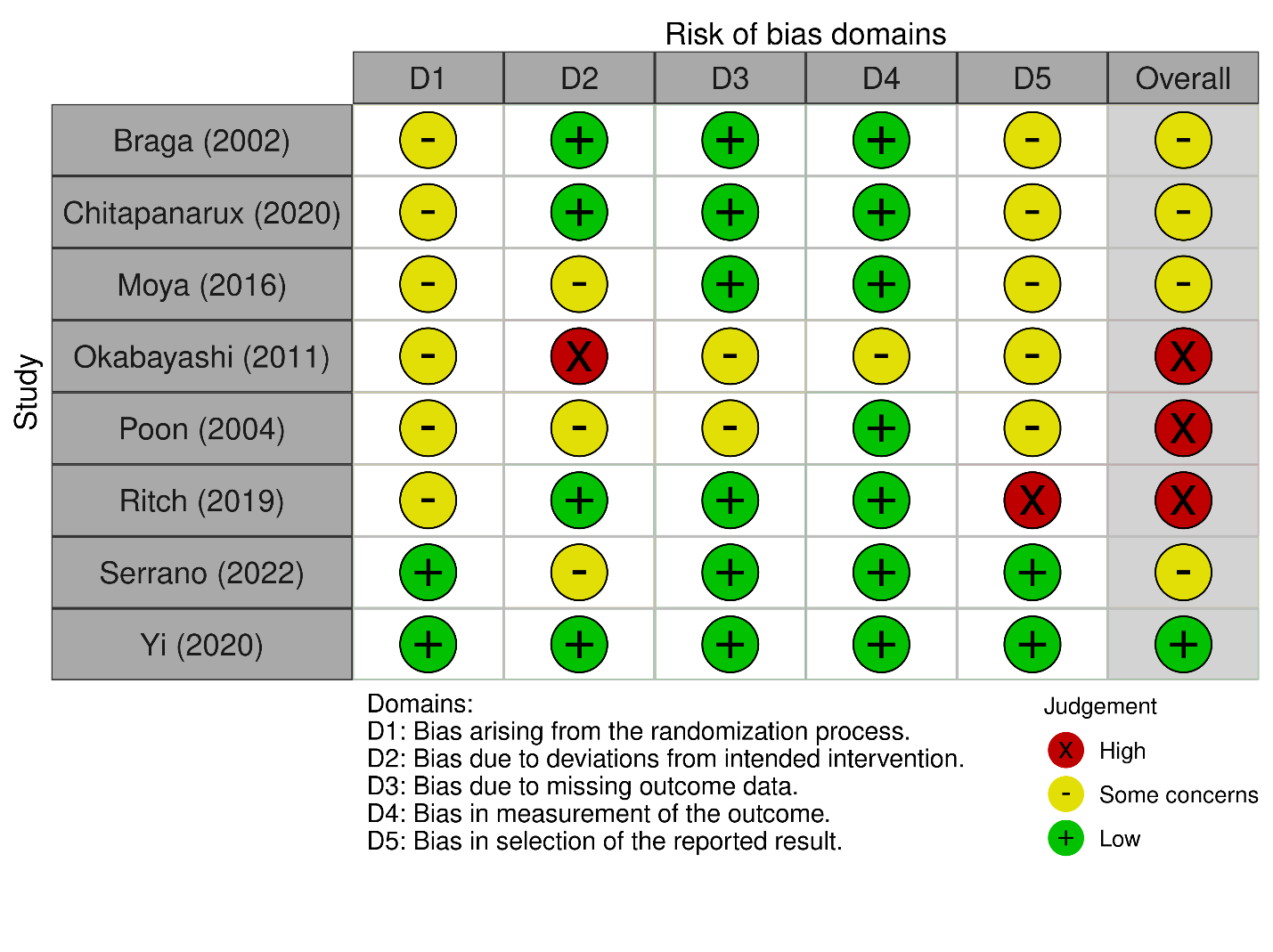


### Quality assessment – Postoperative complications
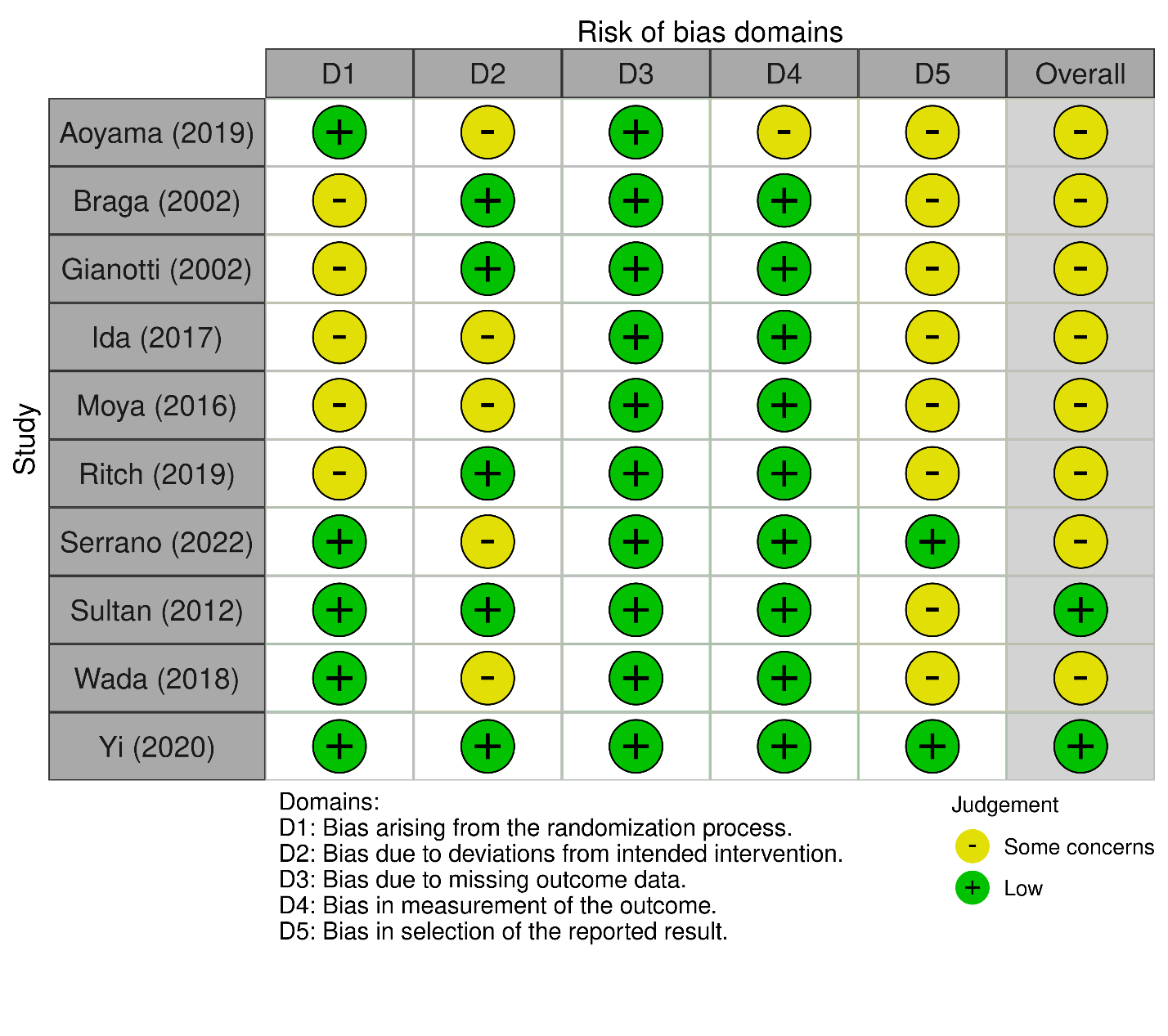


### Quality assessment – Length of stay


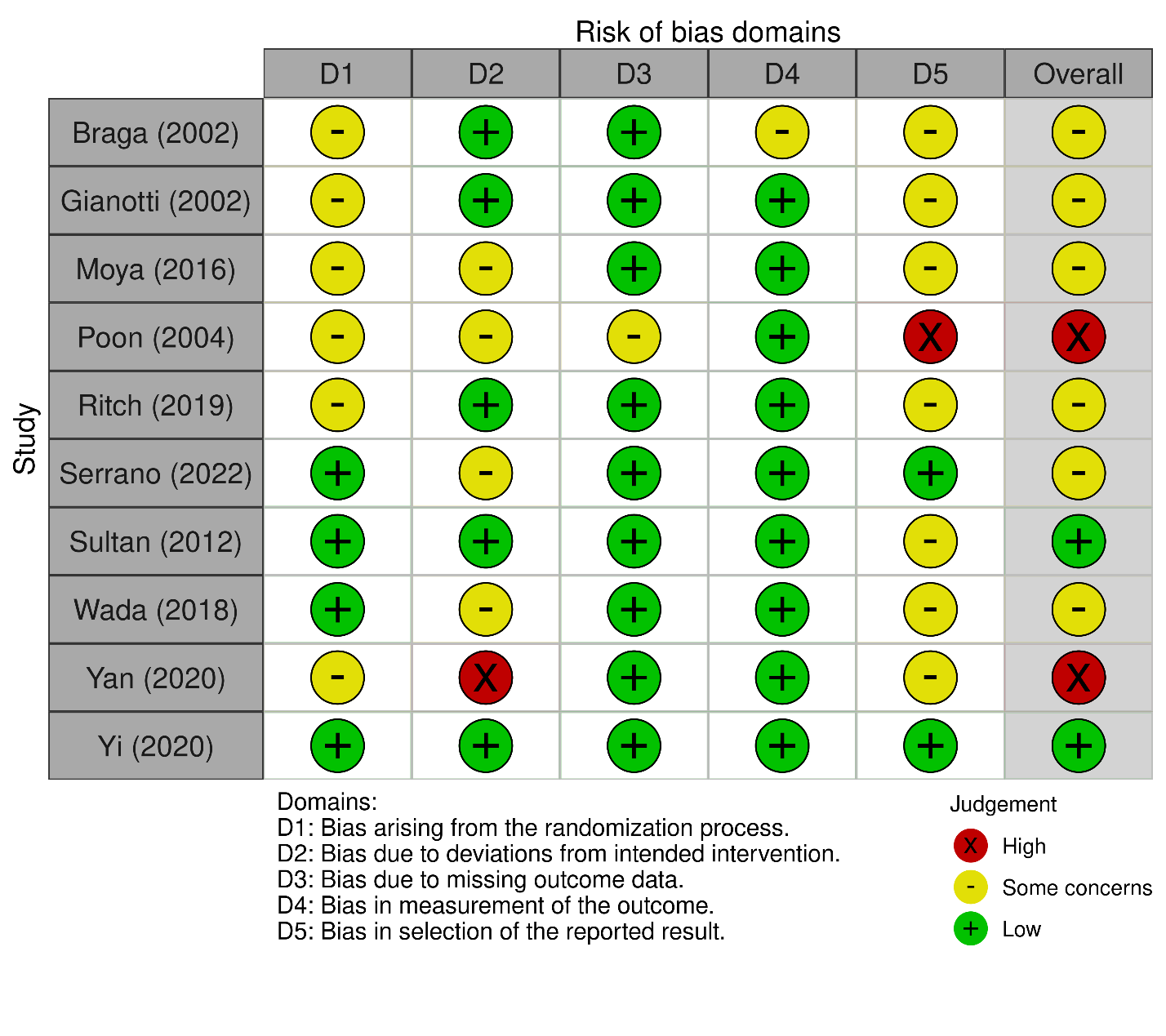


### Quality assessment – Cancer therapy-induced toxicity


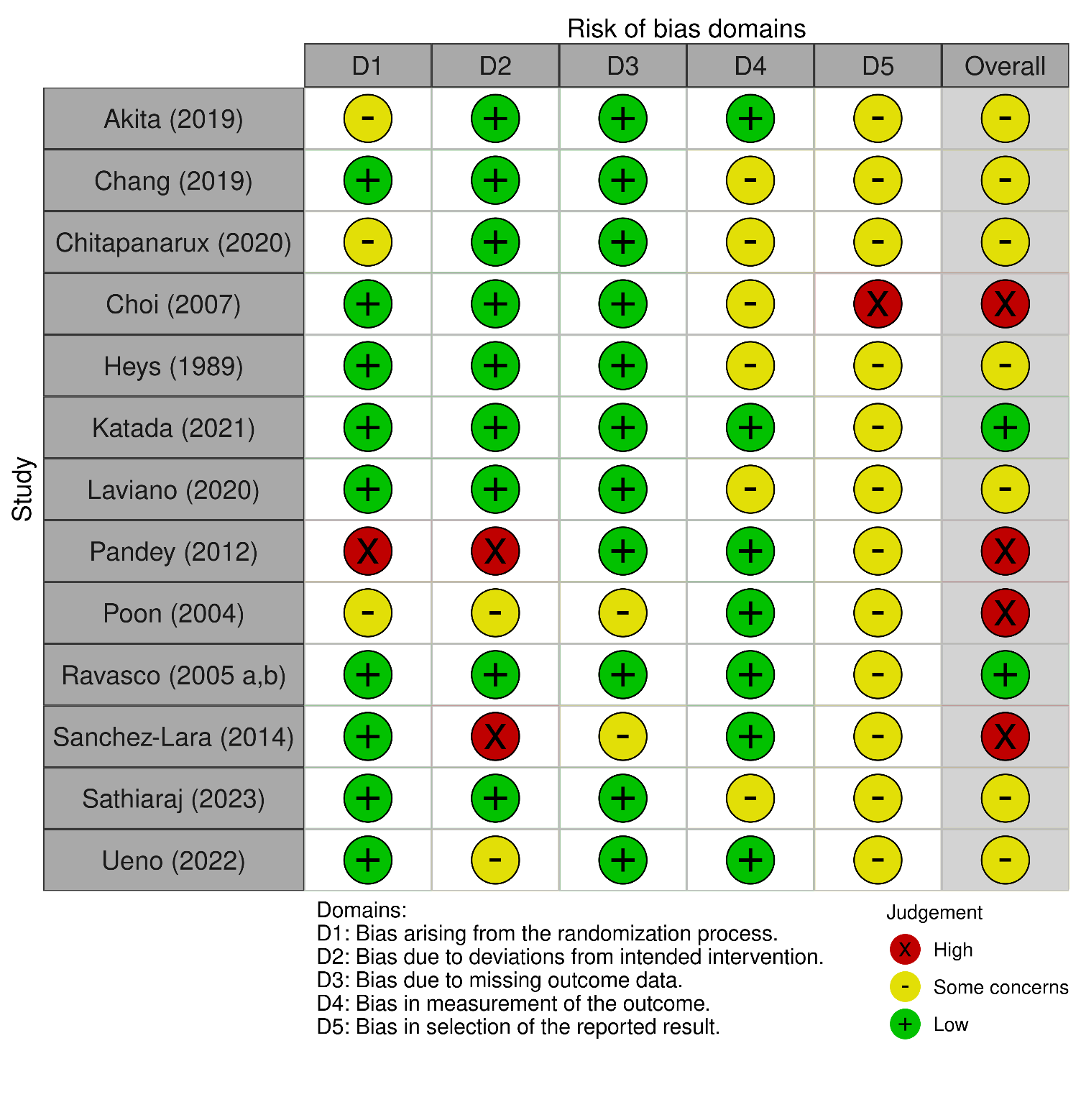


### Quality assessment – Therapy modifications


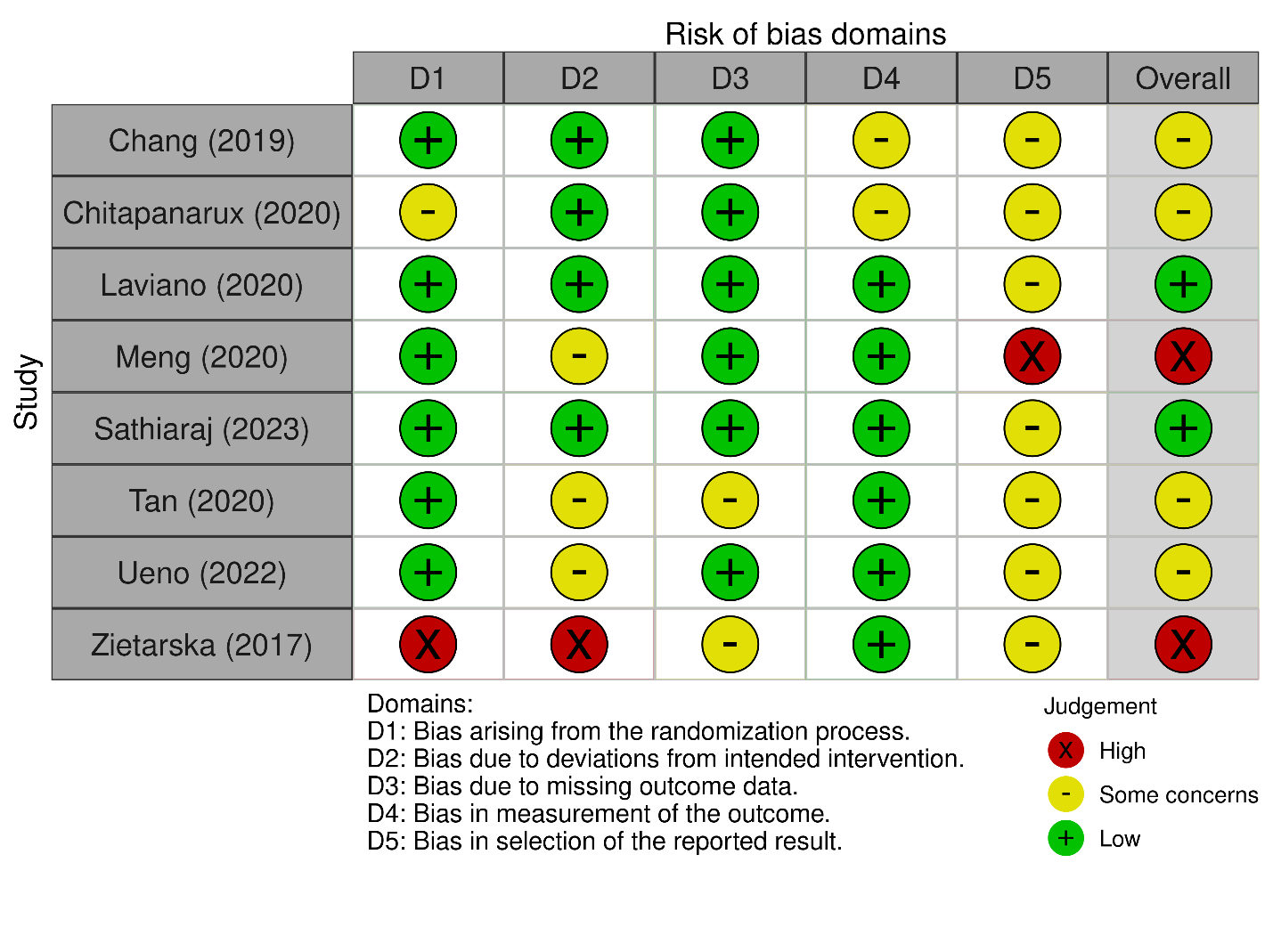


### Quality assessment – Tumor response


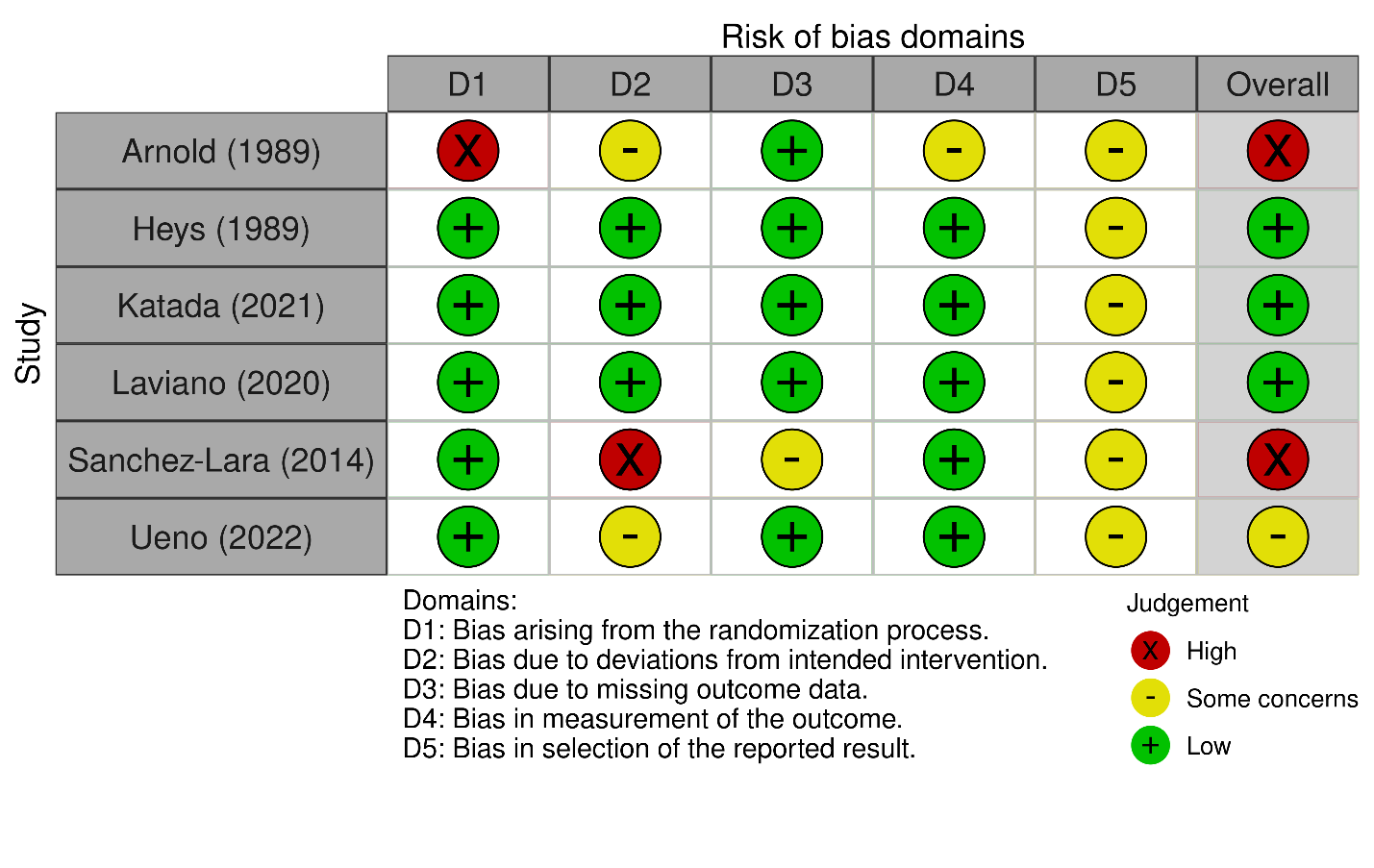


### Quality assessment – Systemic Inflammation


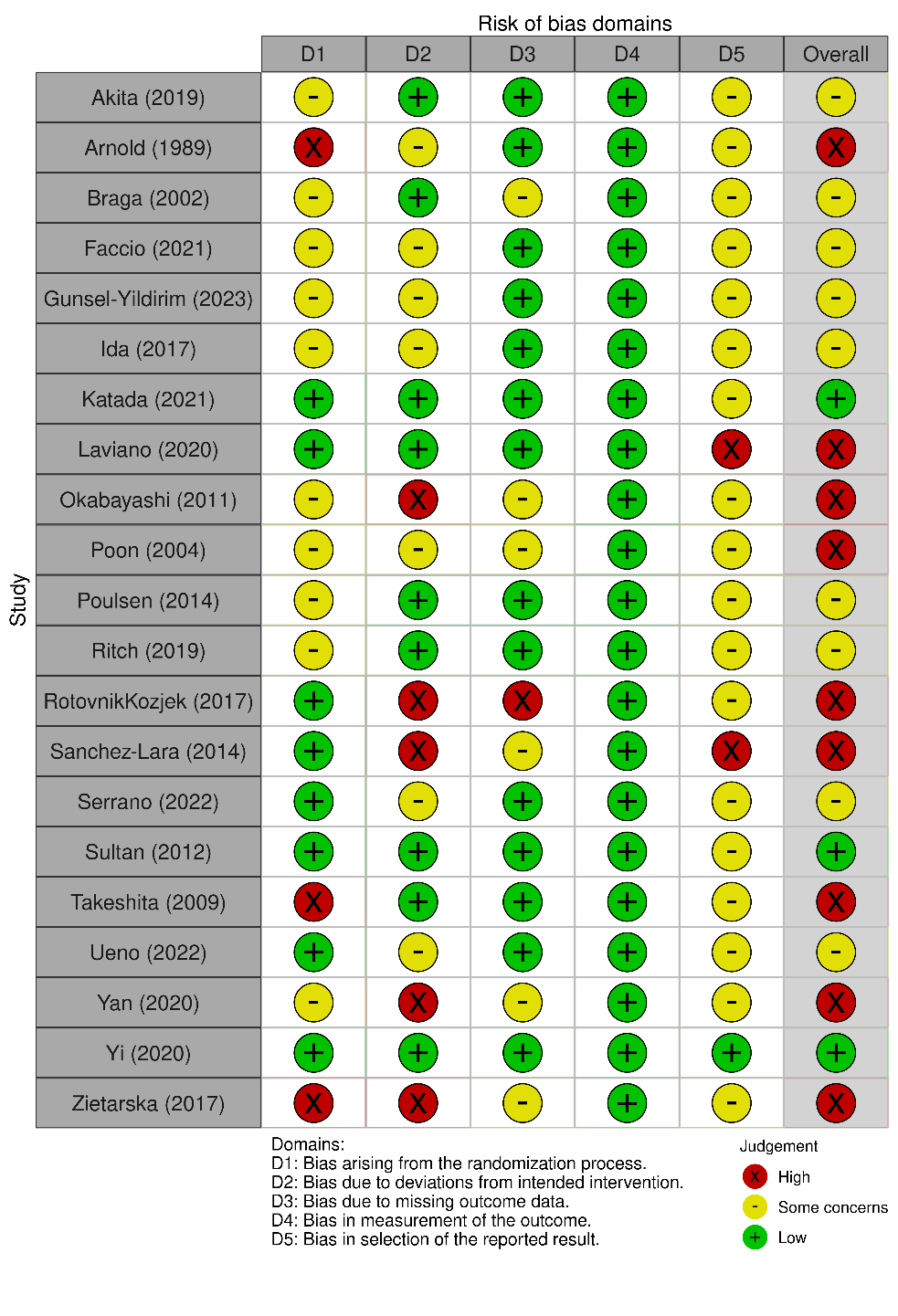


## Supplemental Table 1. Characteristics of the included studies.

| **Reference** | **Study design** | **Sample*,* Total *(*Int, Ctr*)*** | **Baseline population characteristics and setting** | **Cancer therapy** | **Intervention** | **Control** | **Length of intervention** | **Adherence, TEI and protein intakes** |
| --- | --- | --- | --- | --- | --- | --- | --- | --- |
| Sathiaraj, 2023 (1)  India | RCT (open-label, single-center) | 103 (52 Int, 51 Ctr) | Breast cancer; stage I-III; no medical contraindications to adherence to a high-protein diet; PG-SGA scores NR  Age, mean±SD:  51.47±10.14 Int,  51.14±9.76 Ctr | During adjuvant chemotherapy | **Omega-3 ONS** (Kabipro, Fresenius Kabi, India), 1 x (10 g protein [whey], 86 kcal, 0.24 g n-3 PUFAs, 84 kcal)  + Nutrition counseling (weekly) to achieve high-protein (1.2 g/kg BW) using plant-based diet (no meat; eggs and dairy only) | Standard care (regular food intake)  + Nutrition counseling (at least two sessions) | 12 weeks | 90% adhered to the prescribed amount and frequency of whey protein supplement intake. Mean difference in TEI was −330 kcal/d in Int and −4 kcal/d in Ctr. Mean difference in protein intake was +18 g/d in Int and +2.5 in Ctr. |
| Gunsel-Yildirim, 2023 (2)  Turkey | RCT (open-label, single-center) | 70 (35 Int, 35 Ctr) | Lung cancer; stage I-IV; severe malnutrition, low nutritional intake and contraindications for enteral nutrition were exclusion criteria; PG-SGA scores, median (min-max): 2 (0–4) Int, 2 (1–11) Ctr; moderately malnourished or suspected malnutrition: 0% Int, 5.7% Ctr  Age, mean±SD:  61.8±8.7 Int,  62.9±10.5 Ctr | Pre- and post-surgery | **HMB/Arg/Gln** (Abound, Abbott Nutrition, USA), 2 x (1.3 g HMB, 14.8 g protein [7.4 g Arg, 7.4 g Gln], 89 kcal)  + Standard care (energy and protein requirements estimated using Harris-Benedict formula) | Standard care (same as Int arm) | 10 days preoperatively + 5 days postoperatively | NR |
| Serrano, 2022 (3)  Canada | RCT (double-blinded, single-center) | 65 (34 Int, 31 Ctr) | Liver, pancreatic, and colorectal cancer; ASA class IV (67% Int, 71% Ctr); MUST score ≥1 (36% Int, 36% Ctr)  Age, median (IQR):  65 (59−72) Int,  63 (59−68) Ctr | Pre- and post-surgery; adjuvant chemotherapy (27.0% Int vs 19.0% Ctr, received at a median of 60 d and 70 d after surgery, respectively) | **Supplement 1: High-protein ONS** (ISOlution, Enhanced Medical Nutrition, Canada), 2 x (10 g protein [100% whey, 1.4 g Leu], 40 kcal)  **Supplement 2: Arg/Omega-6 ONS**  (INergy-FLD, Enhanced Medical Nutrition, Canada), 3 x (20 g protein, 3.6 g Arg, 0.64 g omega-6, kcal NR)^†^  **Supplement 3: CHO-rich solution** (PreCovery, Enhanced Medical Nutrition, Canada), 3 x (50 g maltodextrin, 200 kcal) | Placebo supplement (collagen-based filler, 0 kcal; similar taste and texture to each supplement) | Supplement 1: 24 days preoperatively (days −30 to −6 of surgery)  Supplement 2: 5 days preoperatively + 5 days postoperatively  Supplement 3: 2 servings in the evening prior to surgery + 1 serving 2−3 h prior to anesthesia | 80% of all patients consumed >70% of study packets |
| Ueno, 2022 (4)  Japan | RCT (open-label, multicenter) | 66 (43 Int, 23 Ctr) | Pancreatic cancer; stage III-IV; ability to maintain food intake >2/3 of normal intake; adequate organ functioning and no serious comorbidities; outpatient  Age, median (IQR):  68 (48−83) Int,  69 (42−84) Ctr | During chemotherapy | **Omega-3 ONS** (Prosure^®^, Abbott, USA), 2 x (16 g protein, 302 kcal, 1.1 g n-3 PUFAs)  If a patient was intolerant to supplements, the number of servings was reduced to 1/day. Patients were allowed to reduce servings based on their appetite. | No supplement | Treatment continued until disease progression, unacceptable toxic effects, or the withdrawal of consent | Mean volume of omega-3 ONS was 1.13 servings/d in Int |
| Faccio, 2021 (14)  Brazil | RCT (open-label, multicenter) | 85 (43 Int, 42 Ctr) | Colorectal, breast, lung, upper digestive tract, ovarian and others  Age, median:  59.16 Int,  58.38 Ctr | Neoadjuvant or adjuvant chemotherapy or concurrent chemoradiotherapy | **Omega-3 ONS** (Immax^®^, Prodiet, Brazil), 3 x (12.5 g protein [61% whey isolate, 28% milk protein isolate, 17% Leu], 197 kcal, 0.15 mg n-3 PUFAs)  + Nutrition counseling (isocaloric diet) | Nutrition counseling (isocaloric diet) | 4 weeks | Average omega-3 ONS intake was 81 g/d in Int, corresponding to 332 kcal/d and 53% of prescribed dose. At 4 weeks, TEI was 207 kcal/d higher in Int (p = 0.03) and 119 kcal/d lower in Ctr (p = 0.22); protein intake was 0.38 g/kg higher in Int (p < 0.01) and 0.06 g/kg higher in Ctr (p = 0.48). |
| Pandey, 2021 (31)  India | RCT (single-blinded, single-center) | 48 (25 Int, 23 Ctr) | Breast cancer; stage N0-N3  Age, mean±SD:  44.1±9.5 Int,  44.4±9.3 Ctr | During neoadjuvant or adjuvant chemotherapy | **Gln** (NR), 2 x (1 g/kg BW Gln) | Placebo supplement (intravenous normal saline) | 5 days | NR |
| Tan, 2021 (5)  China | RCT (open-label, single-center) | 212 (105 Int, 107 Ctr) | Colorectal cancer; stage I-IV; NRS 2002 score ≥3; cardiovascular and respiratory comorbidities, diabetes; outpatient  Age, mean±SD:  59.97±9.18 Int,  58.35±9.29 Ctr | Adjuvant chemotherapy (81.0% Int vs 81.3% Ctr) | **High-protein ONS**  (Nutren^®^ Optimum, Nestle Health Science, Switzerland), 2 x (10.2 g protein [50% whey], 251 kcal)  + Dietary advice to increase intake of fat- and protein-rich food | Dietary advice | 3 months | NR for those receiving chemotherapy |
| Katada, 2021 (6)  Japan | RCT (open-label, single-center) | 71 (36 Int, 35 Ctr) | Esophageal cancer; stage IB-IV; outpatient  Age, mean±SD:  67.8±4.8 Int,  66.7±5.0 Ctr | During chemotherapy | **High-protein ONS**  (ELENTAL^®^, EA Pharma Co., Ltd, Japan), 2 x (13.14 g protein, 300 kcal) | No supplement | 9 weeks | Total high-protein ONS intake was 8661.2±6922.5 kcal in Int over 9 weeks. Blood levels of total protein reduced 1.0 g/dL in Int and 1.1 g/dL in Ctr (p = 0.507). Blood levels of total amino acids reduced 115.5 nmol/mL in Int and 318.6 nmol/mL in Ctr |
| Meng, 2021 (7)  China | RCT (open-label, single-center) | 337 (171 Int, 166 Ctr) | Gastric cancer; stage I-IV; NRS 2002 score ≥3; cardiovascular and respiratory comorbidities, diabetes; outpatient  Age, mean±SD: 60.82±11.50 Int, 59.01±10.93 Ctr | Adjuvant chemotherapy (71.9% Int vs 70.5% Ctr) | **High-protein ONS**  (Nutren^®^ Optimum, Nestle Health Science, Switzerland), 2 x (10.2 g protein, 251 kcal)  + Dietary advice to increase intake of fat and protein-rich food | Dietary advice | 3 months | NR for those receiving chemotherapy |
| Yan, 2021 (8)  China | RCT (open-label, multicenter) | 142 (74 Int, 68 Ctr) | Liver cancer; NRS-2002 score >1 (39.2% Int, 50% Ctr)  Age, mean±SD: 56.07±11.05 Int,  55.44±8.85 Ctr | Pre- and post-surgery | **High-protein ONS**  (TP-MCT, Nutricia, China), 1-2 x (25 g protein x 500 kcal)  + Standard care preoperatively (regular food intake) | Standard care (regular food intake; low-fat diet preoperatively and liquid/semiliquid diet postoperatively) | 3 days preoperatively + 7 days postoperatively | NR |
| Chitapanarux, 2020 (19)  Thailand | RCT (open-label, multicenter) | 88 (44 Int, 44 Ctr) | Nonmetastatic head and neck, esophageal, and cervical cancer; outpatient  Age, median (IQR):  53 (47-60) Int,  59 (50-64) Ctr | During concurrent chemoradiotherapy | **Arg/Gln/Omega-3 ONS** (Neo-Mune, Thai Otsuka Pharmaceutical Co., Thailand), 2 x (15.6 g protein [10.95 g sodium caseinate, 3.13 g Arg, 1.57 g Gln], 250 kcal, n-3 PUFAs NR)  + Nutrition counseling (weekly) to prevent under-nutrition | Regular diet (1500 kcal/d and 60 g/d protein); patients with esophageal cancer: 400 mL of enteral feed with blenderized diet (2000 kcal/d with 75 g/d protein)  + Nutrition counseling (weekly) to prevent under-nutrition | Overall length, median (IQR): 40 (35-49) days | 7 patients interrupted supplementation. All patients achieved 50% of energy requirements (750 kcal) by regular diet |
| Laviano, 2020 (25)  Croatia, Italy, Slovakia, Sweden | RCT (double-blinded, multicenter) | 55 (26 Int, 29 Ctr) | Lung cancer; stage IB-IV; weight loss over the previous 12 months, mean± SD: 3.8±3.6% Int, 4.0±3.9% Ctr; outpatient  Age, mean±SD:  64.4±7.7 Int,  66.0±8.0 Ctr | During first line chemotherapy | **Omega-3 ONS**  (Nutrifriend Cachexia, Smartfish, Norway), 2 x (10 g protein, [10 g whey], 200 kcal, 2.0 g n-3 PUFAs) | Isocaloric placebo ONS supplement, 2 x (6 g protein [milk], 200 kcal) | 12 weeks | Adherence to omega-3-enriched ONS in Int was 58.5% and to placebo ONS in Ctr was 73.6% (p=0.06) |
| Yi, 2020 (13)  Malaysia | RCT (open-label, single-center) | 118 (62 Int, 56 Ctr) | Ovarian, endometrial, cervical, and uterine cancer; stage I-IV; any weight loss over the previous month (% weight change, mean±SD: −4.5±6.8% Int, −5.3±7.2% Ctr); PG-SGA, mean±SD: 6.7±5.2 Int, 7.0±5.5 Ctr; inpatient  Age, mean±SD:  49.5±12.2 Int,  51.2±11.9 Ctr | Pre- and post-surgery | **Supplement 1: High-protein ONS** (NR), 2 x (18 g protein [whey], 500 kcal)  **Supplement 2: High-protein ONS** (NR), 1 x (9 g protein [whey], 250 kcal) | Standard care (12-hour fasting prior to surgery) | Supplement 1: At 12 h prior to and 4 h after surgery  Supplement 2: At 3 h prior to and after pts tolerate 500 ml after surgery  Preoperatively + postoperatively (length of intervention not clearly reported) | Adherence to the ERAS protocol was 96.7−100.0% and to postoperative diet was 100% in Int |
| Akita, 2019 (24)  Japan | RCT (open-label, single-center) | 62 (31 Int, 31 Ctr) | Pancreatic cancer; IIA-IIB; outpatient  Age, mean±SD:  67.8±10.7 Int,  66.4±9.8 Ctr | During neoadjuvant concurrent chemoradiotherapy | **Omega-3 ONS** (Prosure^®^, Abbott, Japan), 2 x (16 g protein, 303 kcal, 1.1 g n-3 PUFAs)  + Dietary advice | Standard care + Dietary advice | 5 weeks | 45.2% of patients in Int consumed ≥50% of the supplement dose  Mean TEI was 1370±630 kcal/d in Int and 1340±400 kcal/d in Ctr (p=0.830). Mean difference from baseline to follow-up was −380 kcal/d in Int and −330 kcal/d in Ctr |
| Chang, 2019 (30)  Taiwan | RCT (double-blinded, single-center) | 60 (30 Int, 30 Ctr) | Lung cancer; stage IIIB-IV  Age, mean±SD:  60.3 ± 18.2 Overall | During concurrent chemoradiotherapy | **Gln** (NR),3 x (10 g protein [10 g Gln])  + Standard care | Standard care | 12 months | NR |
| Ritch, 2019 (32)  United States | RCT (open-label, single-center) | 61 (31 Int, 30 Ctr) | Bladder cancer; stage I-IV  Age, mean±SD:  69.0±14.1 Int,  66.7±9.6 Ctr | Pre- and post-surgery; neoadjuvant chemotherapy (58% Int, 47% Ctr) | **HMB/Omega-3 ONS** (Ensure Clinical Strength, Abbott, USA)  2 x (13 g protein [soy, casein, whey], 350 kcal, 1.5 g HMB, 0.64 g n-3 PUFAs)  + Routine nutrition care postoperatively | Multivitamin (Member’s Mark Multivitamin), twice daily  + Routine nutrition care postoperatively | 3 to 4 weeks preoperatively + 4 weeks postoperatively | Supplement intake adherence was 88% in Int. TEI decreased −162 kcal in Int and −191 kcal in Ctr (p > 0.05). Protein intake decreased −8.6 g/d in Int and −12.9 g/d in Ctr (p = 0.82) |
| Wada, 2018 (29)  Japan | RCT (double-blinded, single-center) | 60 (30 Int, 30 Ctr) | Liver, pancreas, stomach, and bile duct cancer  Age, median (range):  66 (40-81) Int,  69 (25-81) Ctr | Pre- and post-surgery | **HMB/Arg/Gln** (Abound, Abbott, Japan), 1 x (14 g protein [7 g Arg, 7 g Gln], 79 kcal, 1.5 g HMB)  + Routine nutrition care (regular hospital diet) | Isocaloric placebo supplement (Bireley’s, Asahi Soft Drinks, Tokyo, Japan) with  a similar taste  + Routine nutrition care (regular hospital diet) | 3 days preoperatively + 7 days postoperatively | Patients consumed 95% and 90% of the planned volume in Int and Ctr, respectively |
| Ida, 2017 (10,11)  Japan | RCT (open-label, multicenter) | 123 (63 Int, 60 Ctr) | Gastric cancer; stage IA-IIIC; inpatient  Age, median (range):  65.1 (31–79) Int,  65.6 (30–80) Ctr | Pre- and post-surgery | **Omega-3 ONS** (Prosure^®^, Abbott, Japan), 2 x (16 g protein, 302 kcal, 1.1 n-3 PUFAs) | Standard perioperative diet | 7 days preoperatively + 21 days postoperatively | Relative performance (*[actual dose * 100] / planned dose*) was 100% before surgery and 54% after surgery in Int |
| Rotovnik Kozjek, 2017 (28)  Slovenia | RCT (double-blinded, single-center) | 73 (33 Int, 40 Ctr) | Rectal cancer; stage T2-T4, N0-N3; at risk of malnutrition by NRS-2002: 12.1% Int, 12.5% Ctr  Age, mean±SD:  60.8±11.9 Int,  61.4±9.9 Ctr | Neoadjuvant concurrent chemoradiotherapy | **Gln** (Glutamine Supplement, Peeroton, Austria), 3 x (10 g protein [10 g Gln]) | Placebo supplement (30 g/d maltodextrin) | 5 weeks | Patients consumed an average of 0.40±0.06 g/kg/d Gln |
| Ziętarska, 2017 (12)  Poland | RCT (open-label, single-center) | 72 (38 Int, 34 Ctr) | Colorectal cancer; <10% weight loss over the previous 6 months; SGA score, mean±SD: 4.68±0.91 Int, 4.68±0.91 Ctr; NRS-2002: 3.19±0.77 Int, 3.02±0.56 Ctr; all patients had pre-cachexia; outpatient  Age, mean±SD:  65.00±9.97 Int,  63.66±9.39 Ctr | During first line chemotherapy | **High-protein ONS** (NR), 2 x (18 g protein, 300 kcal) | No supplement | 12 weeks | 80% of patients consumed >75% of the high-protein ONS in Int |
| Moya, 2016 (22)  Spain | RCT (open-label, single-center) | 122 (61 Int, 61 Ctr) | Colorectal cancer; outpatient preoperatively, inpatient postoperatively  Age, median (range):  69 (51–85) Int,  68 (45–92) Ctr | Pre- and post-surgery | **Arg/Omega-3 ONS** ((IEF)-ATEMPERO^®^, Vegenat^®^, Spain), 2 x (16.6 g protein [2 g Arg], 302 kcal, 1.54 g n-3 PUFAs)  + Dietary advice + ERAS protocol | Dietary advice + ERAS protocol | 7 days preoperatively + 5 days postoperatively | All patients in Int consumed 400 ml/d of the preoperative supplement. Postoperatively, 88.5% of patients in Int consumed 400 ml/d of the supplement |
| Poulsen, 2014 (20)  Denmark | RCT (open-label, single-center) | 61 (32 Int, 29 Ctr) | Gynecological, gastric, and esophageal cancer; weight loss prior to intervention: 4 pts lost <5% and 15 lost ≥5% in Int, 3 pts lost <5% and 16 lost ≥5% in Ctr; outpatient  Age, median (IQR):  62 (53–68) Int,  64 (57–70) Ctr | During palliative or preoperative chemotherapy, radiotherapy, or concurrent chemoradiotherapy | **Omega-3 ONS** (Forticare^®^, Nutricia, The Netherlands), 3 x (11.3 g protein [casein, whey, 1.8 g nitrogen], 204 kcal, 0.37 g n-3 PUFAs)  + Nutrition counseling (weekly) to meet protein and energy requirements | + Dietary advice by nurses with the possibility to schedule an appointment with a dietitian if needed | 5 to 12 weeks | 50% of patients in Int consumed ≥75% of recommended dose. At the end of treatment, calorie intake and protein intake (p < 0.001) were greater in Int than Ctr |
| Sanchez-Lara, 2014 (21)  Mexico | RCT (single-blinded, single-center) | 92 (46 Int, 46 Ctr) | Lung cancer; stage III-IV; weight loss over the previous 6 months, mean±SD: 8.8±8% Int, 7.1±9% Ctr; outpatient  Age, mean±SD:  58.8±14 Int,  61±12.4 Ctr | During first line chemotherapy | **Omega-3 ONS** (ProSure^®^, Abbott, USA), 2 x (16 g protein, 302 kcal, 1.1 g n-3 PUFAs)  + Isocaloric diet using standardized menus of 1400, 1600, 1800, 2000 or 2200 kcal | Isocaloric diet using standardized menus of 1400, 1600, 1800, 2000 or 2200 kcal | Over two chemotherapy cycles ± 7 days | Mean intake of Omega-3 ONS was 1.4±0.6 servings/d. More than 73% of patients consumed 2 servings/d of the supplement. After the second chemotherapy cycle, mean difference in TEI was +72 kcal/d in Int (p = 0.597) and −344 kcal/d in Ctr (p = 0.08); mean difference in protein intake was +0.1 g/d in Int (p = 0.657) and −12 g/d in Ctr (p = 0.04) |
| Sultan, 2012 (23)  United Kingdom | RCT (double-blinded, multicenter) | 195 (66 Int Arm 1, 63 Int Arm 2, 66 Ctr) | Esophageal or gastric cancer; median ASA class II; >10% weight loss 3 months preoperatively (12% Int Arm 1; 5% Int Arm 2; 8% Ctr); outpatient preoperatively, inpatient postoperatively  Age, median (IQR):  67 (42−79) Int Arm 1,  60 (42−79) Int Arm 2,  66 (43−84) Ctr | Pre- and post-surgery; neoadjuvant chemotherapy (56% Int Arm 1; 57% Int Arm 2; 52% Ctr) | **Int Arm 1: Omega-3 ONS** (Oxepa^®^, Abbott, UK), 675 mL/d (2.8 bottles of 237 mL/d) = 2.8 x (14.8 g protein [casein], 356 kcal, 1.09 g n-3 PUFAs)  **Int Arm 2: Omega-3 ONS** (Ensure Plus^®^, Abbott Nutrition, UK)  675 mL/d (2.8 bottles of 237 mL/d) = 2.8 x (16.0 g protein [soy protein, cow’s milk], 350 kcal, 0.65 g n-3 PUFAs) | Isotonic liquid feed (Osmolite^®^, Abbott  Nutrition, UK) without immunonutrients and lower energy (1 kcal/ml) and protein (4 g per 100 ml) | Int Arm 1 & 2: 7 days preoperatively + 7 days postoperatively  Ctr: 7 days postoperatively | Predicted vs. actual protein intake (g/kg BW/d), mean±SD:  Arm 1: 1.20±0.15 vs. 0.72±0.33  Arm 2: 1.21±0.12 vs. 0.62±0.27  Ctr: 1.21±0.12 vs. 0.55±0.21  TEI and protein intake on postoperative day 7 differed between study arms (p = 0.001)  Postoperatively, 46.7% of all patients reached the maximum feeding rate |
| Baldwin, 2011 (9)  United Kingdom | RCT (open-label, multicenter) | 358 (86 Int ARM_1_, 86 Int ARM_2_, 96 Ctr ARM_3_, 90 Ctr ARM_4_) | Gastrointestinal, lung, and mesothelioma cancer; advanced stage; outpatient  Age, median (IQR):  66.8 (24.0–88.0) Overall | Chemotherapy | **Int ARM_1_: Option 1: High-protein ONS** (ScandiShake SHS International, UK), 1 x (12.5 g protein x 585 kcal)  **Option 2: High-protein ONS** (Calshake (Fresenius-Kabi, UK), 1 x (12 g protein x 599 kcal)  + Multivitamin supplements (Forceval, Unigreg, Morden, UK or Supradyn;  Roche, Welwyn, UK)  **Int ARM_2_: High-protein ONS** as give to ARM_1_ + Dietary advice to increase food intake by 600 kcal/day | **Ctr ARM_3_:** No intervention  **Ctr AMR_4_:** Dietary advice to increase food intake by 600 kcal/day | 6 weeks | 40% of all patients returned supplement diaries, showing that 31% and 19% of patients consumed all supplement at weeks 1 and 6, respectively |
| Okabayashi, 2011 (18)  Japan | RCT (open-label, single-center) | 76 (40 Int, 36 Ctr) | Liver cancer; inpatient and outpatient  Age, mean±SD:  68.7±7.6 Int,  65.1±11.3 Ctr | Pre- and post-surgery | **BCAA** (Aminoleban EN, Otsuka Pharmaceutical Company, Japan), 2 x (13.5 g protein [6.5 g AAs, 6.5 g peptides, 0.5 g casein], 210 kcal) | Isocaloric diet | 2 weeks preoperatively + 6 months postoperatively | NR |
| Takeshita, 2009 (17)  Japan | RCT (open-label, single-center) | 56 (28 Int, 28 Ctr) | Liver cancer; inpatient  Age, mean±SD:  69.1±8.231 Int,  70.6±9.745 Ctr | During TACE | BCAA (Aminoleban EN, Otsuka Pharmaceutical Company, Japan), 1 x (13.5 g protein [6.5 g Aas, 6.5 g peptides, 0.5 g casein], 210 kcal) | No supplement | 1 day before TACE until 2 weeks after TACE | NR |
| Choi, 2007 (33)  Republic of Korea | RCT (open-label, NR) | 51 (22 Int, 29 Ctr) | Stomach, esophageal, colorectal, periampullary, biliary, head and neck, and others  Age, median (range):  54 (26-73) Int,  54 (26-79) Ctr | During chemotherapy | **Gln** (Daesang Wellife Seoul, Korea), 3 x (10 g protein [10 g Gln]) | Best supportive care | 15 days | NR |
| Ravasco, 2005 (35–36)  Portugal | RCT  (open-label, single-center) | 111 (37 Int, 37 Ctr ARM_1_, 37 Ctr ARM_2_) | Colorectal cancer; stage I-IV; moderately or severely malnourished by PG-SGA: 37.8% Int, 35.1% Ctr ARM_1_, 40.5% Ctr ARM_2_  Age, mean±SD:  58±15 Overall | During concurrent chemoradiotherapy | **High-protein ONS** (NR), 2 x (20 g protein, 200 kcal)  Participants selected flavors and used the same commercial brand throughout the study | Ctr ARM_1_: No intervention (ad libitum intake)  Ctr ARM2: Nutrition counseling based on regular foods | 6 weeks | At follow-up, protein intake increased +30 g/d and TEI +296 kcal/d in Int; protein decreased −10 g/d and TEI −285 kcal/d in Ctr ARM_1_; protein increased +27 g/d and TEI +555 kcal/d in Ctr ARM_2_ |
| Ravasco, 2005  (37)  Portugal | RCT  (open-label, single-center) | 75 (25 Int, 25 Ctr ARM_1_, 25 Ctr ARM_2_) | Head and neck cancer; stage I-IV; moderately or severely malnourished by PG-SGA: 56% Int, 60% Ctr ARM_1_, 64% Ctr ARM_2_  Age, mean±SD:  60±11 Overall | During radiotherapy | **High-protein ONS** (NR), 2 x (20 g protein, 200 kcal)  Participants selected flavors and used the same commercial brand throughout the study | Ctr ARM_1_: No intervention (ad libitum intake)  Ctr ARM2: Nutrition counseling based on regular foods | 7 weeks | At follow-up, protein intake increased +35 g/d and TEI +322 kcal/d in Int; protein decreased −15 g/d and TEI −400 kcal/d in Ctr ARM_1_; protein increased +26 g/d and TEI +521 kcal/d in Ctr ARM_2_ |
| Poon, 2004 (16)  China | RCT (single-blinded, single-center) | 84 (41 Int, 43 Ctr) | Liver cancer; Okuda stage I-II; >10% weight loss: 17% Int, 19% Ctr; diabetes mellitus and chronic respiratory, cardiovascular or renal diseases; outpatient  Age, median (range):  59 (24–84) Int,  59 (27–80) Ctr | During TACE | **BCAA** (Aminoleban EN, Otsuka Pharmaceutical Company, Japan), 2 x (13.5 g protein [6.5 g AAs, 6.5 g peptides, 0.5 g casein], 210 kcal)  + Standard care (usual diet) | Standard care (usual diet) | 1 week before first TACE and continued for up to 1 year (4 sessions of TACE maximum) | Intake of BCAA was satisfactory for all patients in Int. At follow-up, median difference in TEI was −1.6 kcal/kg/d in Int and −1.5 kcal/kg/d in Ctr (p ≥ 0.05); median difference in protein intake was −0.02 g/kg/d in both Int and Ctr (p ≥ 0.05) |
| Braga, 2002 (26)  Italy | RCT (NR, NR) | 100 (50 Int, 50 Ctr) | Colorectal cancer; 10% pts lost >10% of weight in Int, and 12% pts lost >10% in Ctr over the previous 6 months; outpatient  Age, mean±SD:  60.5±11.5 Int,  63.0±8.1 Ctr | Pre- and post- surgery | **Arg/Omega-3 ONS**  (Oral Impact, Novartis Consumer Health, Switzerland), 1000 mL/d (4 sachets of 74 g dissolved in 250 ml of water each) = 4 x (18 g protein [100% whey, 3.13 g Arg], 309 kcal, 0.825 g n-3 PUFAs) | No intervention | 5 days preoperatively + 4 days postoperatively | Mean preoperative supplement intake was 890 mL/d. Postoperatively, 82% of patients reached the target supplement dose |
| Gianotti, 2002 (27)  Italy | RCT (open-label, single-center) | 203 (101 Int, 102 Ctr) | Gastrointestinal cancer; weight loss over the previous 6 months, mean±SD: 2.5 ± 2.7% Int, 2.4 ± 2.6% Ctr; outpatient  Age, mean±SD:  65.6±11.5 Int,  63.4±11.9 Ctr | Pre- and post- surgery | **Arg/Omega-3 ONS** (Oral Impact, Novartis Consumer Health, Switzerland)  Preoperatively: 1000 mL/d (4 sachets of 74 g dissolved in 250 ml of water each) = 4 x (18 g protein [100% whey, 3.13 g Arg], 309 kcal, 0.825 g n-3 PUFAs)  Postoperatively:  1500 mL/d (6 sachets of 74 g dissolved in 250 ml of water each) = 6 x (18 g protein [100% whey, 3.13 g Arg], 309 kcal, 0.825 g n-3 PUFAs)  Formula was administered via jejunal feeding within 12 hours after surgery and continued until patients resumed oral food intake | No preoperative intervention  + Postoperative intravenous solution of 5% glucose and electrolytes (28 mL/kg/d) until oral intake is resumed | 5 days preoperatively + 12 hours postoperatively or until patients resumed oral food intake | Mean preoperative supplement intake was 925 mL/d. Tolerance to postoperative jejunal infusion was 88.1% |
| Heys, 1998 (34)  United Kingdom | RCT (double-blinded, single-center) | 96 (48 Int, 48 Ctr) | Breast cancer; stage T2-T4  Age, mean (range):  49 (31-73) Int,  50 (30-72) Ctr | During neoadjuvant chemotherapy; surgery and radiotherapy when indicated | **Arg** (NR), 3 x (10 g protein [10 g Arg]) | Placebo supplement (composition NR) | 3 days prior to each 21-day chemotherapy cycle for a total of 6 cycles | NR |
| Arnold, 1989 (15)  United States | RCT (open-label, single-center) | 50 (23 Int, 27 Ctr) | Head and neck cancer; stage I-IV; no significant pre-treatment weight loss prior to intervention; outpatient  Age, median (range):  64.1 (34-88) Int,  68.3 (43-80) Ctr | During radiotherapy | **High-protein ONS**  (Sustacal™ liquid, Nestle Health Science, Switzerland), 4 x (10.2 g protein [50% whey, 50% casein], 251 kcal)    + Nutrition counseling | Nutrition counseling | 10 weeks | 30.4% of patients in Int consumed ≥80% of high-protein ONS. Mean intake was 67% and 56% of prescribed dose in males and females, respectively. Mean difference in TEI between groups at follow-up was 300 kcal/d (p = 0.07) |

* Study with mixed population; reported data correspond to the overall study population (not specific to patients with cancer). ^†^ Information on supplement composition provided by the company.

Abbreviation list: AAs, amino acids; Arg, arginine; ASA, American Society of Anesthesiologists; BW, body weight; CHO, carbohydrate; Ctr, control group; ERAS, Enhanced Recovery After Surgery; Gln, glutamine; HMB, β-hydroxy β-methylbutyrate; Int, intervention group; IQR, interquartile range; Leu, leucine; MUST, Malnutrition Universal Screening Tool; n-3 PUFAs, n-3 Polyunsaturated Fatty Acids; NR, not reported; NRS-2002, Nutrition Risk Screening 2002; ONS, oral nutritional supplement; PG-SGA, Patient-Generated Subjective Global Assessment; pts, patients; RCT, randomized controlled trial; SD, standard deviation; SGA: subjective global assessment; TACE, transarterial chemoembolization; TEI, total energy intake.

## Supplemental Table 2. Adverse events reported in the included studies.

| **Author, Year** | **Intervention**  Supplement type, number of servings/day, protein per serving | **Control** | **Length of intervention** | **Adverse events related to the high-protein supplement** |
| --- | --- | --- | --- | --- |
| Serrano, 2022 | Supplement 1: High-protein ONS  2 x (10 g protein [100% whey, 1.4 g Leu])  Supplement 2: Arg/Omega-6 ONS  3 x (3.6 g Arg, 0.64 g omega-6 fatty acids)  Supplement 3: CHO-rich solution  3 x (50 g maltodextrin) | Placebo (collagen-based filler, 0 kcal; similar taste and texture to each supplement) | Supplement 1: High-protein ONS: 24 days preoperatively (days −30 to −6 of surgery)  Supplement 2: Arg/Omega-3 ONS: 5 days preoperatively + 5 days postoperatively  Supplement 3: CHO-rich solution: 2 servings in the evening prior to surgery + 1 serving 2−3 h prior to anesthesia | Patients in the experimental and control groups experienced nausea (73% vs. 57%, respectively), which was a common reason for non-compliance with the postoperative supplement. However, this was related to the higher proportion of participants in the experimental group undergoing pancreatectomies (35% vs 7%). |
| Ueno, 2022 | **Omega-3 ONS**  2 x (16 g protein, 1.1 g n-3 PUFAs)    If patient were intolerant to supplements, number of servings was reduced to 1/day. Patients were allowed to reduce servings based on their appetite. | No supplement | Treatment continued until disease progression, unacceptable toxic effects, or the withdrawal of consent | No serious adverse events were recorded, and there were no differences between groups in gastrointestinal symptoms. |
| Faccio, 2021 | **Omega-3 ONS**  3 x (12.5 g protein [61% whey isolate, 28% milk protein isolate, 17% L-leucine], 0.15 mg n-3 PUFAs) | Nutritional counseling (isocaloric diet) | 4 weeks | Patients in the experimental group reported diarrhea (7%), constipation (2.3%), and abdominal pain (2.3%) based on onset with start of supplement consumption and resolution with supplement discontinuation. |
| Pandey, 2021 | Gln  2 x (1 g/kg body weight Gln) | Placebo (intravenous normal saline) | 5 days | Among all patients and independently of cause, fatigue (84.7%), anorexia (62.5%), and cough (9.7%) were common. However, none of the patients discontinued treatment due to adverse events. |
| Laviano, 2020 | **Omega-3 ONS**  2 x (10 g protein, [10 g whey], 2.0 g n-3 PUFAs) | Placebo ONS (milk-based isocaloric comparator drink) | 12 weeks | Patients in the experimental group experienced constipation (3.8%) and dyspepsia (3.8%). Patients in the control group (Placebo ONS) experienced diarrhea (3.4%), nausea 3.4%, and vomiting (3.4%). Despite this, the authors stated that the supplement was well tolerated with a favorable safety profile. |
| Yi, 2020 | **Supplement 1: High-protein ONS**  2 x (18 g protein [whey])  **Supplement 2: High protein ONS**  1 x (9 g protein [whey]) | Standard care (12-hour fasting prior to surgery) | Supplement 1: At 12 h prior to and 4 h after surgery  Supplement 2: At 3 h prior to and after pts tolerate 500 ml after surgery  Preoperatively + postoperatively (length of intervention not clearly reported) | No serious adverse events were recorded, and there was a low anesthetic risk (vomiting/nausea) of drinking the supplement 3 hours preoperatively. |
| Chang, 2019 | **Gln** (NR),3 x (10 g protein [10 g Gln])  + Standard care | Standard care | 12 months | All patients tolerated the supplement well. |
| Wada, 2018 | **HMB/Arg/Gln**  1 x (14 g protein [7 g Arg, 7 g Gln], 1.5 g HMB) | Isocaloric placebo + Routine nutritional care (regular hospital diet) | 3 days preoperatively + 7 days postoperatively | Patients with supplement intake ≥ 80% of planned volume showed increased growth hormone levels compared to controls. |
| Rotovnik Kozjek, 2017 | **Gln**  3 x (10 g protein [10 g glutamine]) | Placebo supplement (30 g/d maltodextrin) | 5 weeks | No adverse events were recorded. |
| Zietarska, 2017 | **High-protein ONS**  2 x (18 g protein) | No supplement | 12 weeks | The high-protein ONS supplement was well-tolerated. There were no differences between experimental and control groups in number and severity of nausea, vomiting, diarrhea, abdominal pain. |
| Poulsen, 2014 | **Omega-3 ONS**  3 x (11.3 g protein [casein, whey, 1.8 g nitrogen], 0.37 g n-3 PUFAs) | Dietary advice by nurses with the possibility to schedule an appointment with a dietitian if needed | 5 to 12 weeks | Among all patients, side effects included loss of appetite (28.4%), nausea (16.7%) and flatulence (7.5%). However, there was no significant difference in side effects between groups. |
| Sanchez-Lara, 2014 | **Omega-3 ONS**  2 x (16 g protein, 1.1 g n-3 PUFAs) | Isocaloric diet using standardized menus of 1400, 1600, 1800, 2000 or 2200 kcal | Over two chemotherapy cycles ± 7 days | Patients in the experimental group (omega-3 ONS) did not have an increased frequency of diarrhea compared to controls. |
| Sultan, 2012 | **Int ARM 1: Omega 3-ONS**  2.8 x (14.8 g protein [casein], 1.09 g n-3 PUFAs)  **Int Arm 2: Omega-3 ONS**  2.8 x (16.0 g protein [soy protein, cow’s milk], 0.65 g n-3 PUFAs) | Isotonic liquid feed without immunonutrients and lower energy (1 kcal/ml) and protein (4 g per 100 ml) | Arms 1 & 2: 7 days preoperatively + 7 days postoperatively  Ctr: 7 days postoperatively | Preoperatively, patients reported disliking the supplement due to taste, bloating and/or nausea. Postoperatively, 46.7% of the patients reached the maximum planned feeding rate due to issues related to tolerance and/or complications such as diarrhea, ileus, nausea, vomiting, or bloating. |
| Okabayashi, 2011 | **BCAA**  2 x (13.5 g protein [6.5 g AAs, 6.5 g peptides, 0.5 g casein], 210 kcal) | Isocaloric diet | 2 weeks preoperatively + 6 months postoperatively | The BCAA supplement was well tolerated by all patients. |
| Gianotti, 2002 | **Arg/Omega-3 ONS**  Preoperatively:  4 x (18 g protein [100% whey, 3.13 g Arg], 309 kcal, 0.825 g n-3 PUFas)  Postoperatively:  6 x (18 g protein [100% whey, 3.13 g Arg], 0.825 g n-3 PUFas)  Formula was administered via jejunal feeding within 12 hours after surgery and continued until patients resumed oral food. | No intervention preoperatively, intravenous solution of 5% glucose and electrolytes (28 mL/kg/day) until the day of recovery of oral food | 5 days preoperatively + 12 hours postoperatively or until patients resumed oral food | Patients receiving postoperative jejunal infusion of high-protein supplement experienced more abdominal cramping and bloating (41.6%) than controls (13.7%); however, rates of diarrhea (6.9% vs. 2.9%) and vomiting (2% vs. 2%) did not differ between groups. |
| Heys, 1998 | **Arg**  3 x (10 g protein [10 g Arg]) | Placebo supplement | 3 days preoperatively + 6 pulses at 21 days intervals | Patients in the experimental group experienced diarrhea (data not reported). |

Abbreviations: Arg, arginine; BCAA, branched-chain amino acids; CHO: Carbohydrate; Gln, glutamine; HMB, β-hydroxy β-methyl butyrate; ONS, oral nutritional supplement. ; n-3 PUFAs, n-3 Polyunsaturated Fatty Acids.

## Supplemental Figure 1. Summary of clinical characteristics of patients with cancer included in the systematic review and meta-analysis.


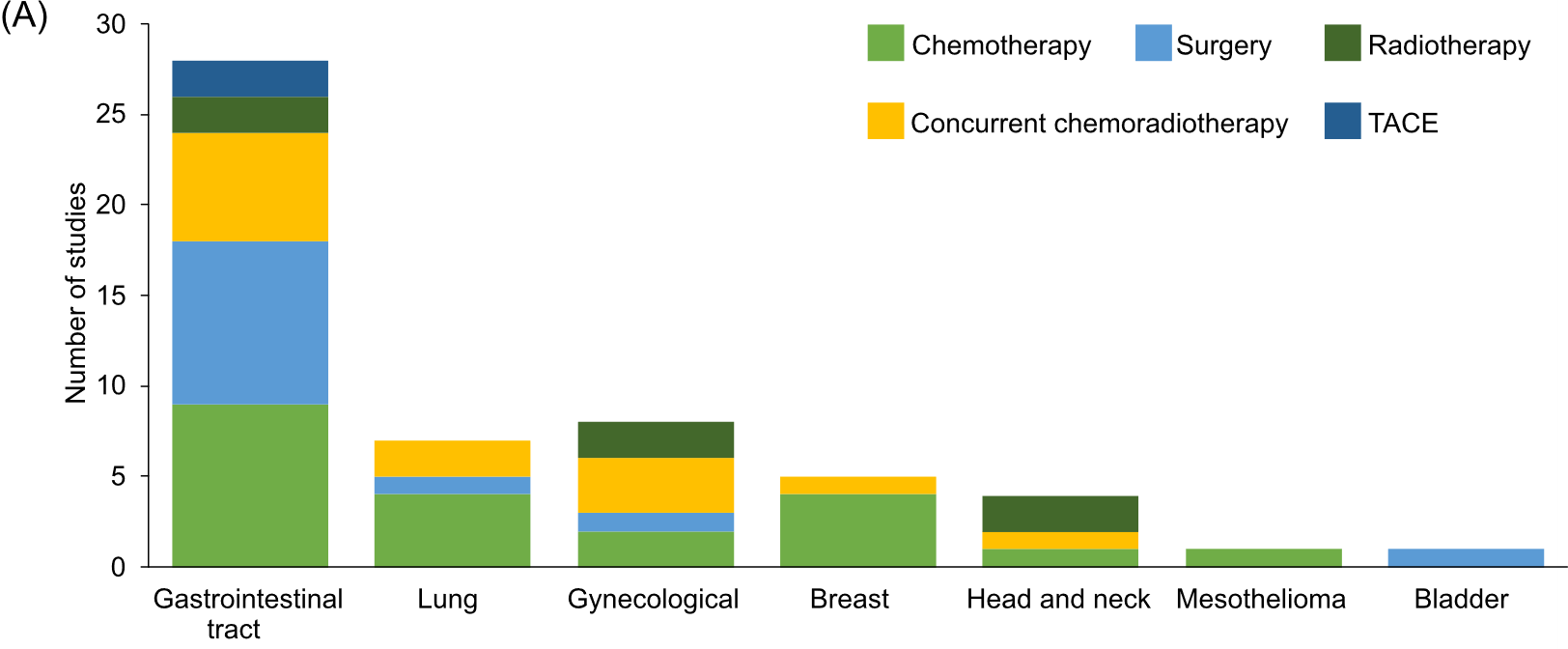


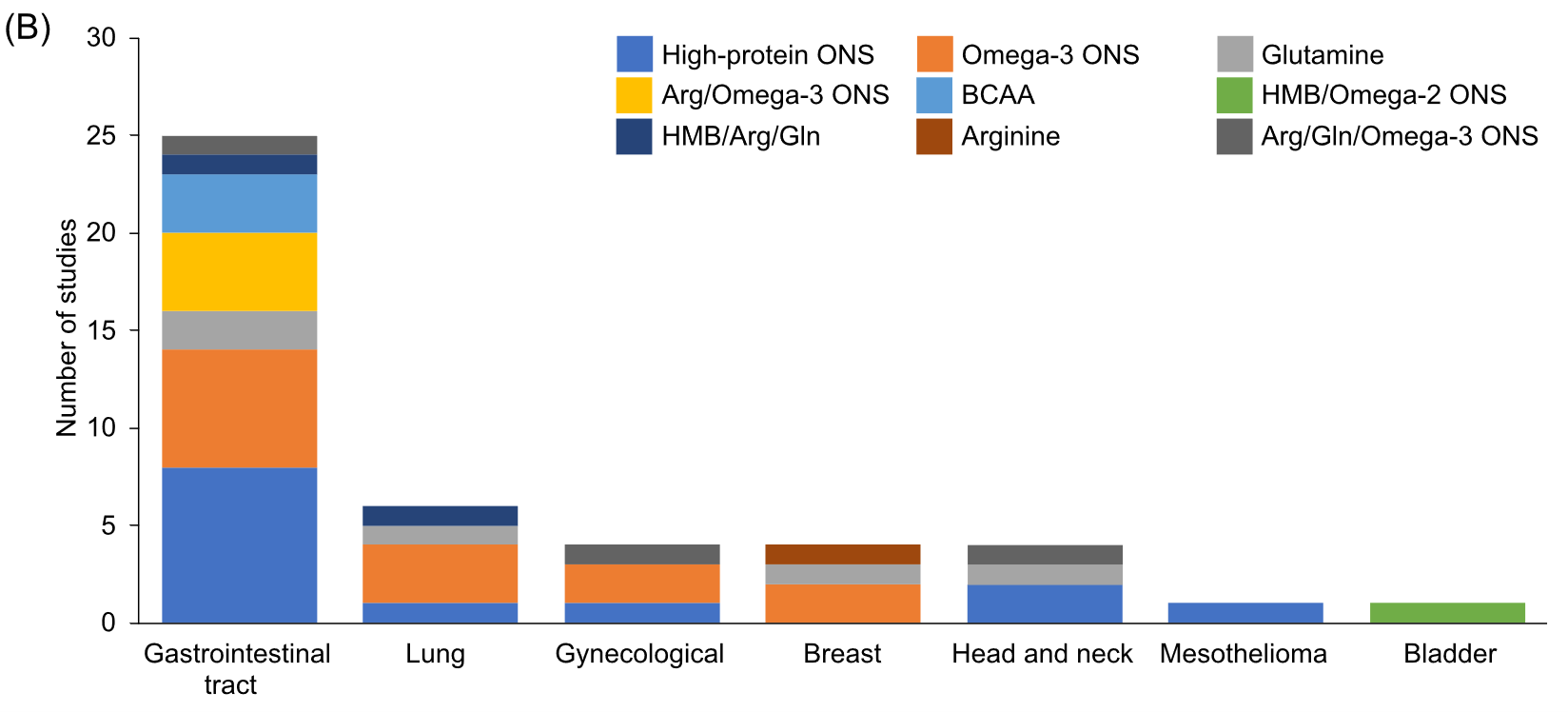


Abbreviations: Arg, arginine; BCAA, branched-chain amino acids; Gln, glutamine; HMB, *β*-hydroxy *β*-methylbutyrate; ONS, oral nutritional supplement. TACE, transarterial chemoembolization.


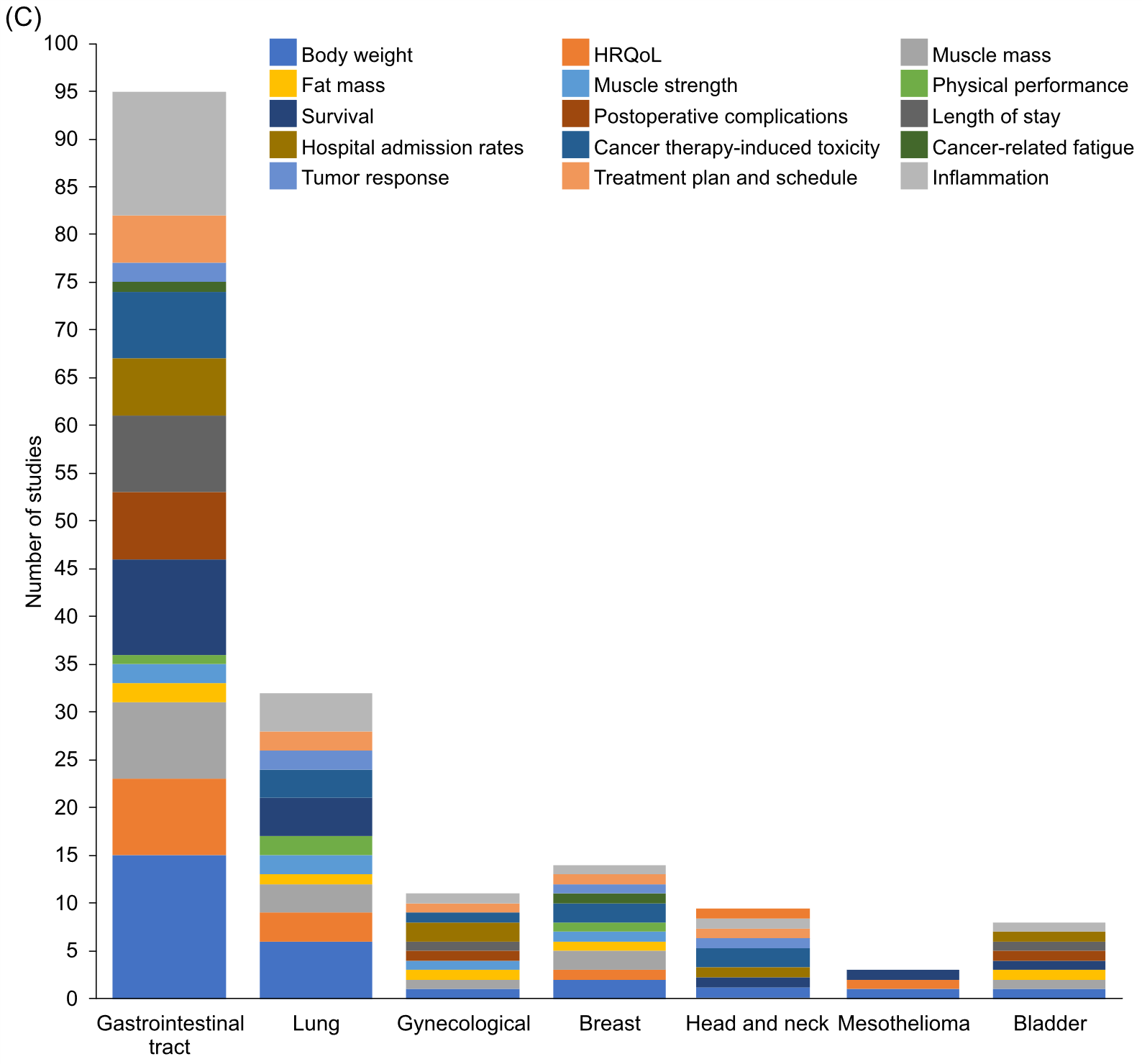


(A) Number of studies stratified by cancer types and modalities of cancer therapy. (B) Number of studies stratified by cancer types and high-protein supplements administered. (C) Number of studies stratified by cancer type and outcomes evaluated. Abbreviations: HRQoL: Health-related quality of life.

## Supplemental Figure 2. Summary of characteristics of high-protein supplements.


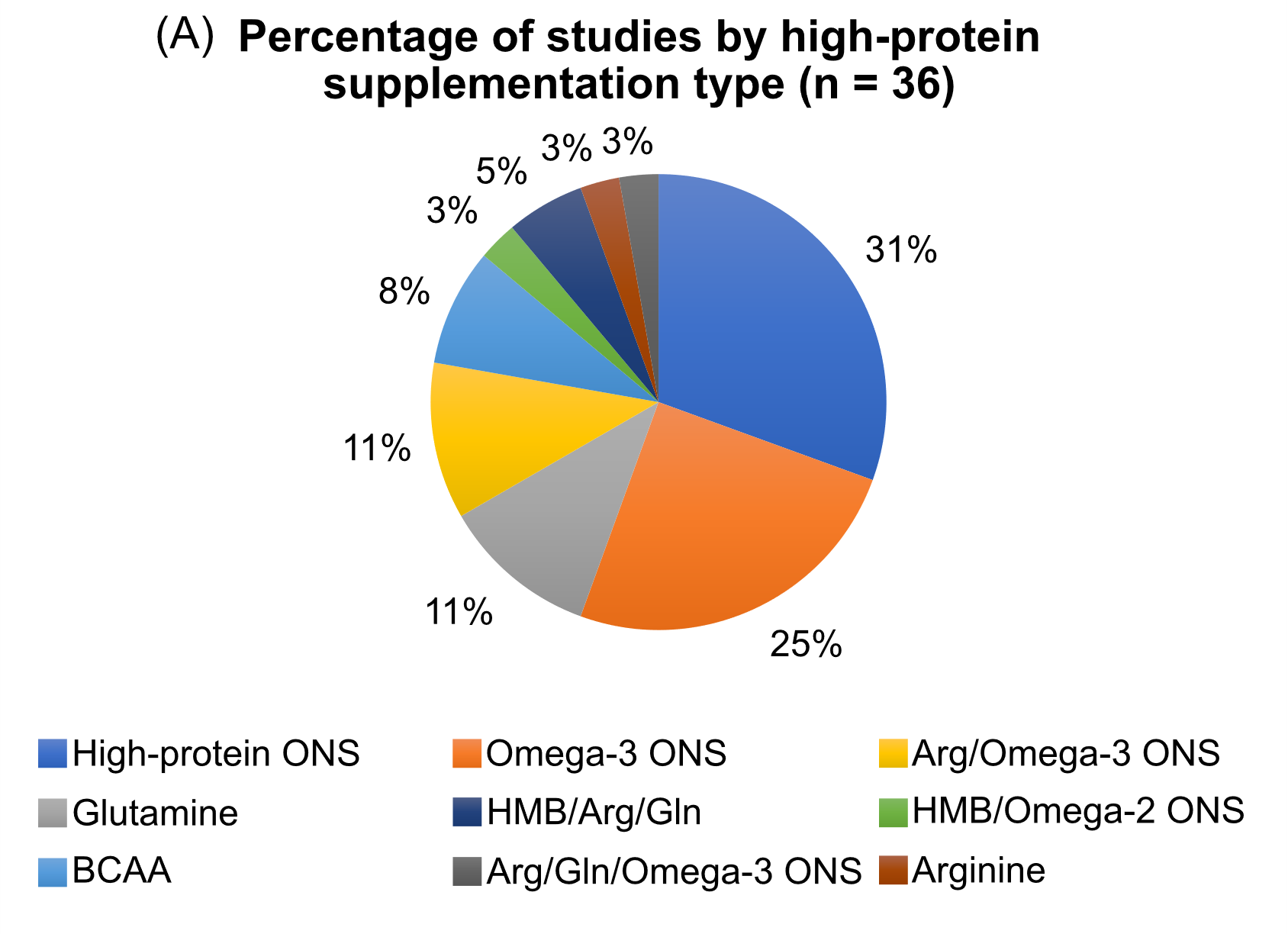


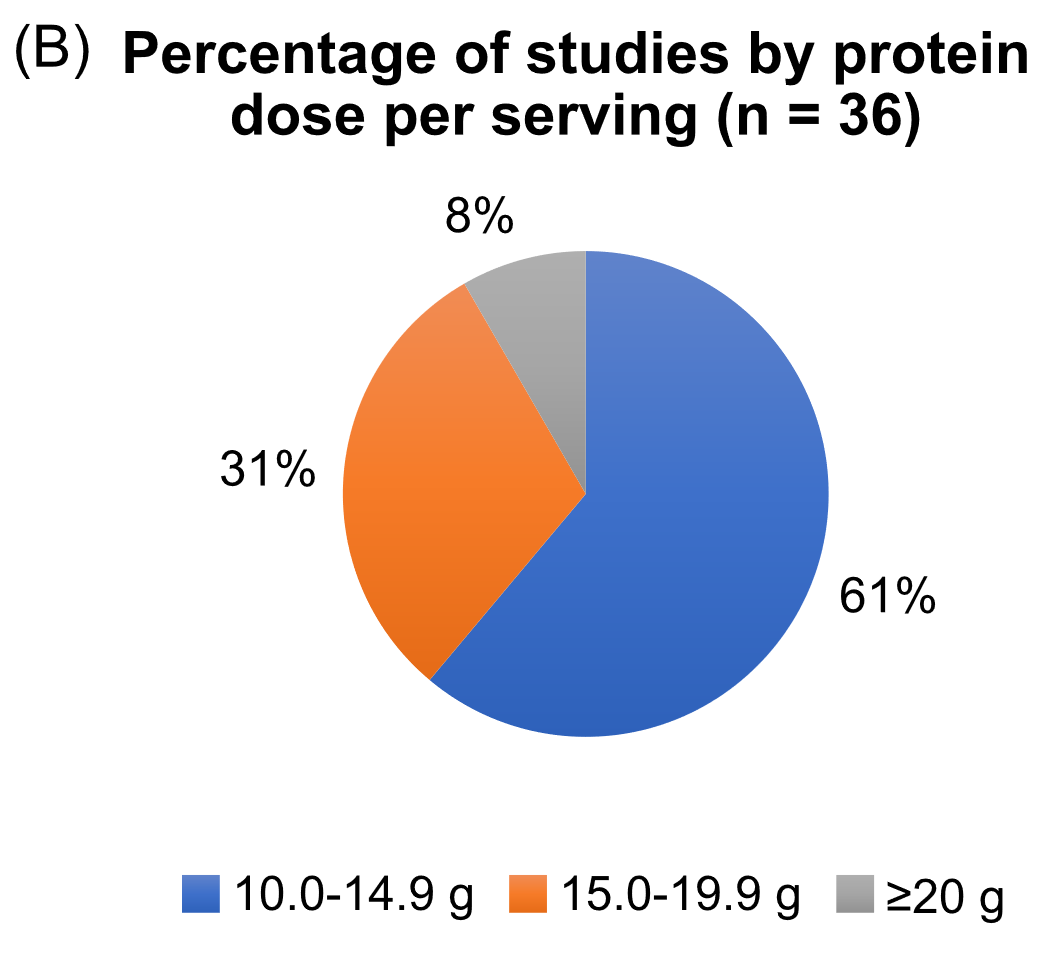

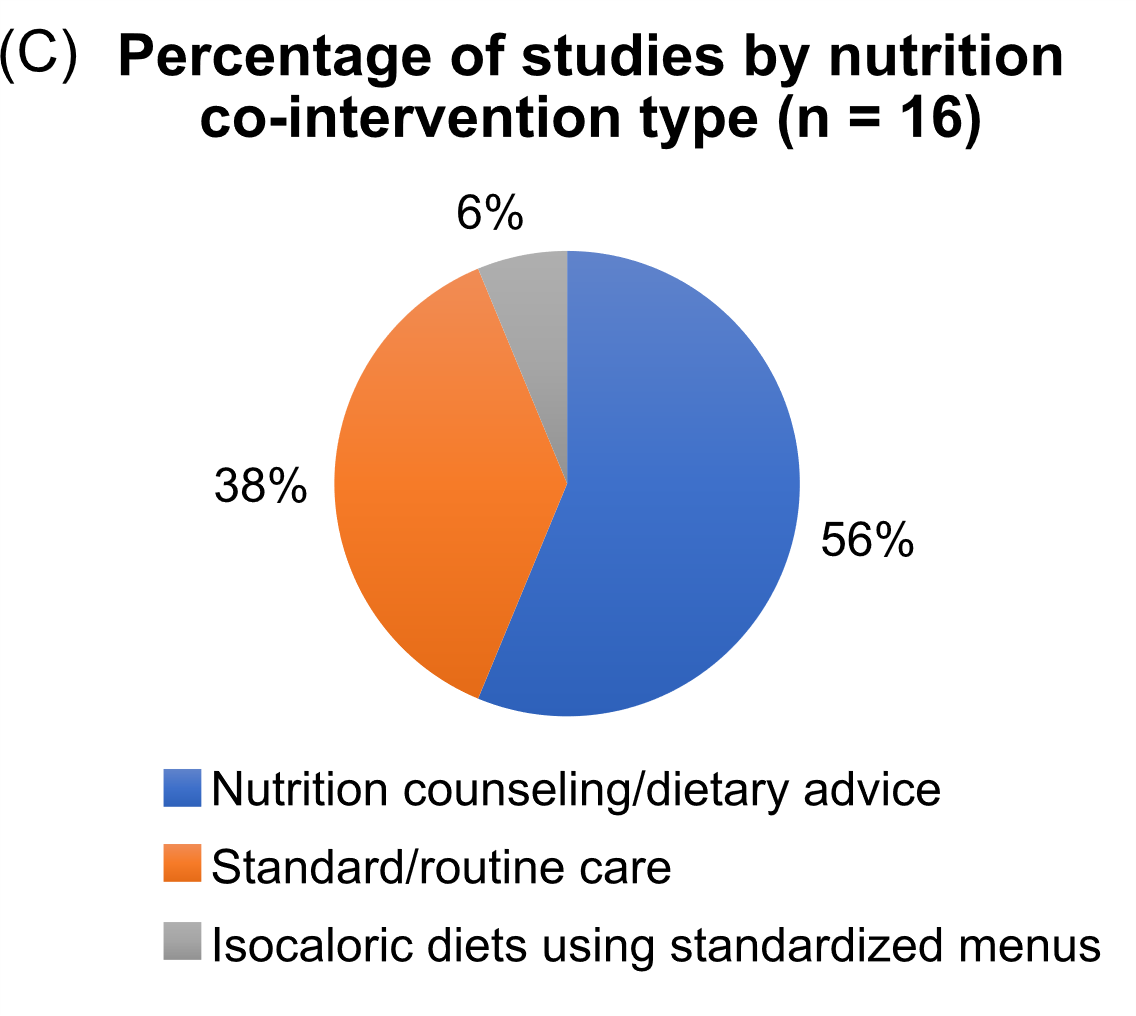


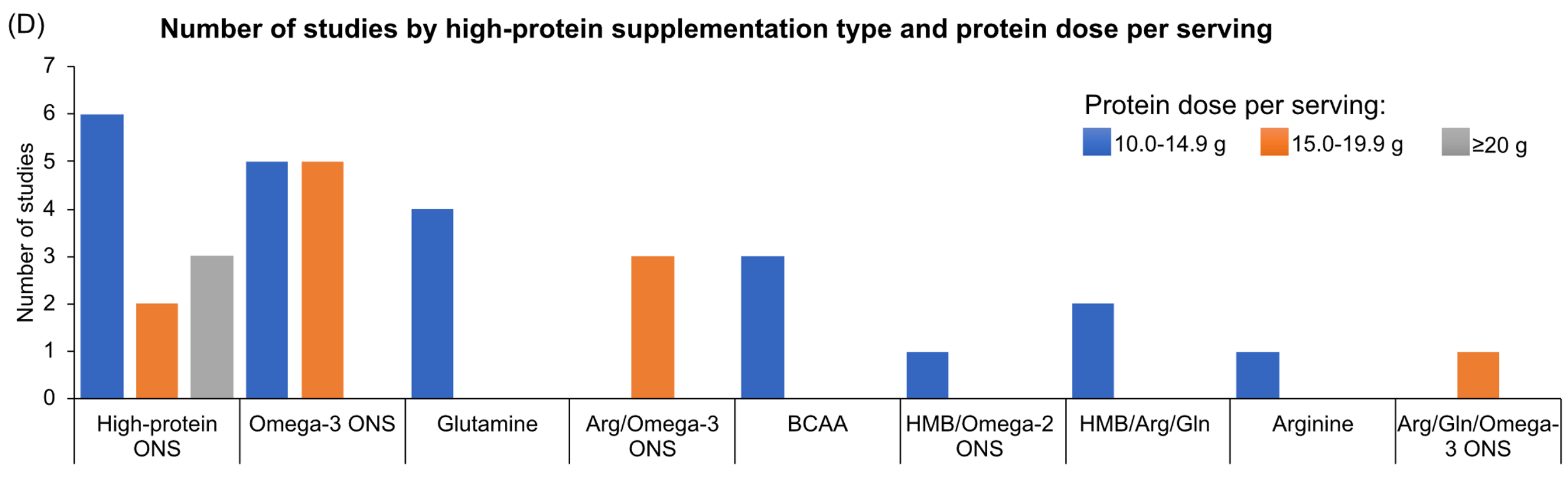


Abbreviations: Arg, arginine; BCAA, branched-chain amino acids; Gln, glutamine; HMB, *β*-hydroxy *β*-methylbutyrate; ONS, oral nutritional supplement.

## Supplemental **Figure 3.** Meta-analysis of the effects of high-protein supplementation on body weight in kilograms, percentage change, or BMI.

**
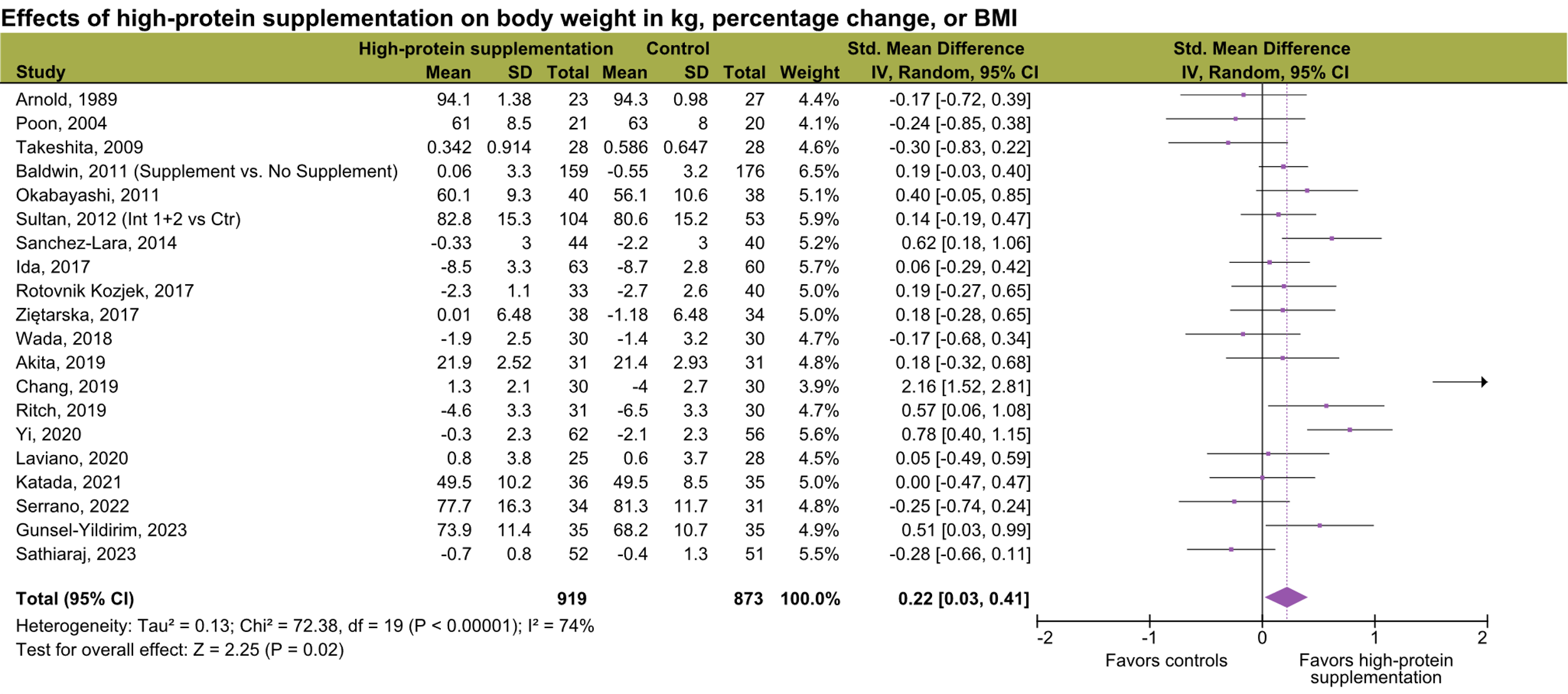
**

## Supplemental Figure 4. Subgroup meta-analyses of the effects of high-protein supplementation on body weight (in kg) based on total daily protein intake from high-protein supplement and based on the duration of high-protein supplementation

**
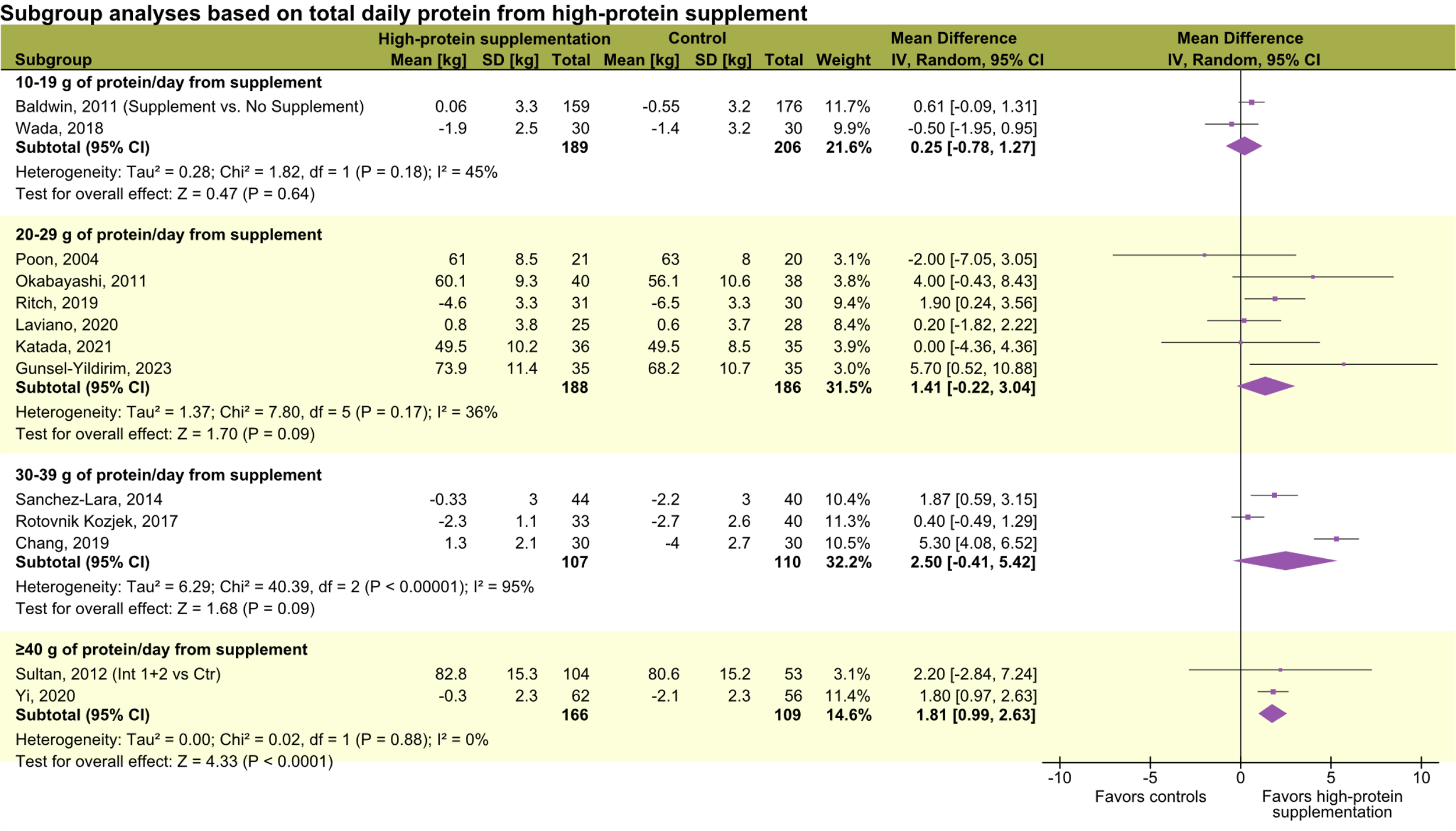
**


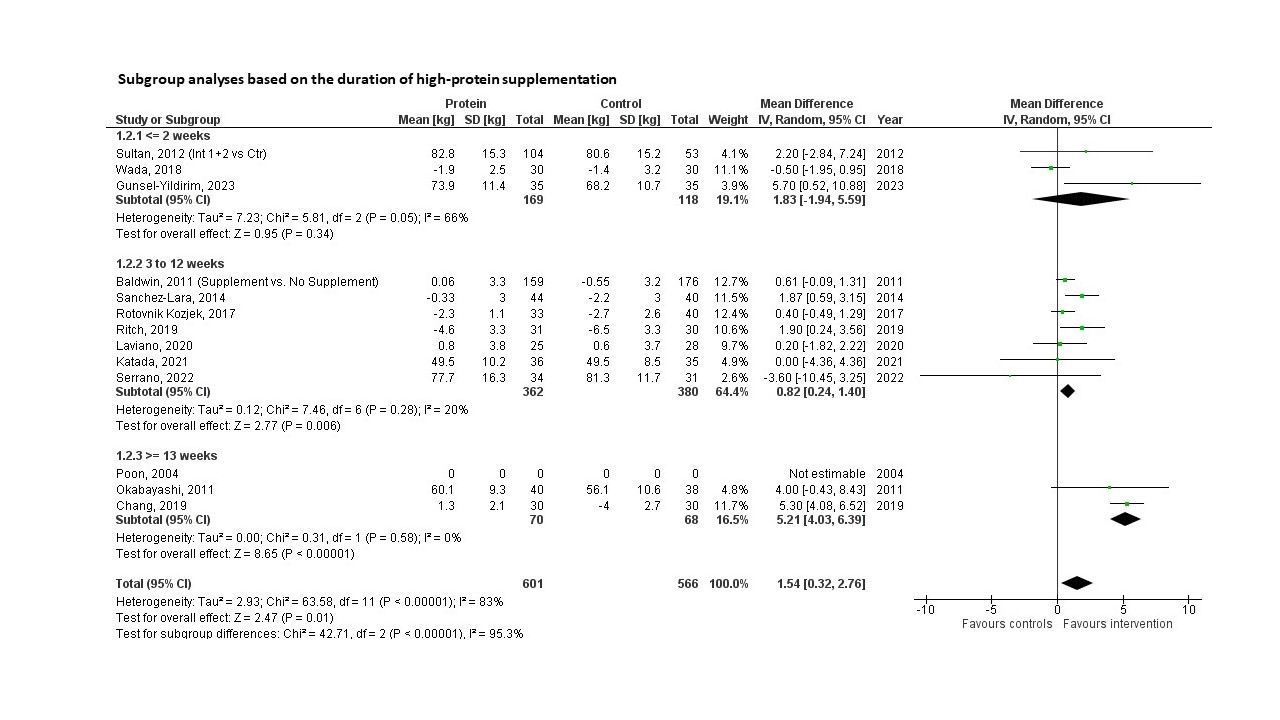


## Supplemental Figure 5. Subgroup meta-analyses of the effects of high-protein supplementation on body weight (in kg) based on cancer and treatment types.


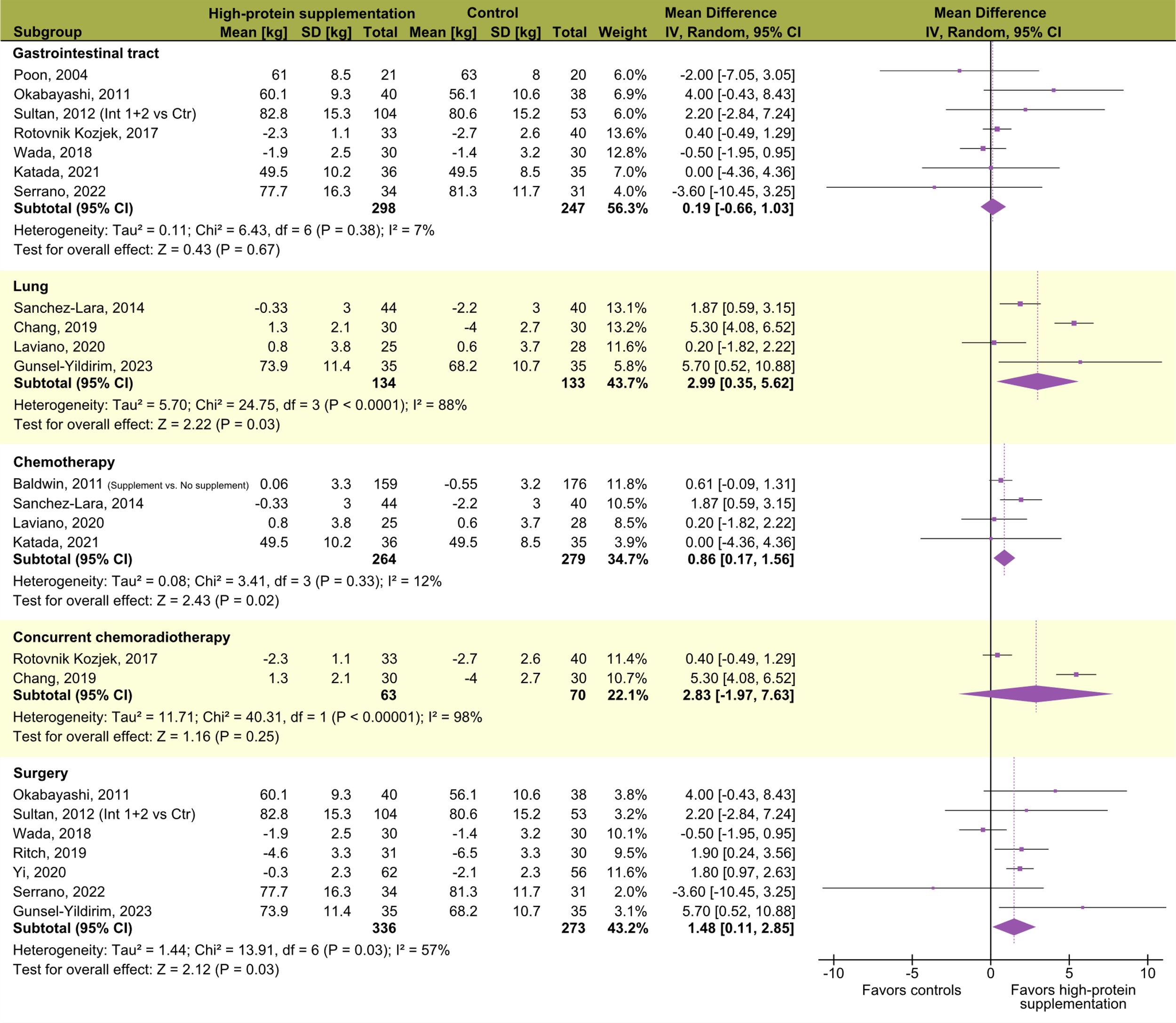


## Supplemental **Figure 6.** Subgroup meta-analyses of the effects of high-protein supplementation on body weight (in kg) based on pre-intervention nutrition status and changes in inflammation.

**
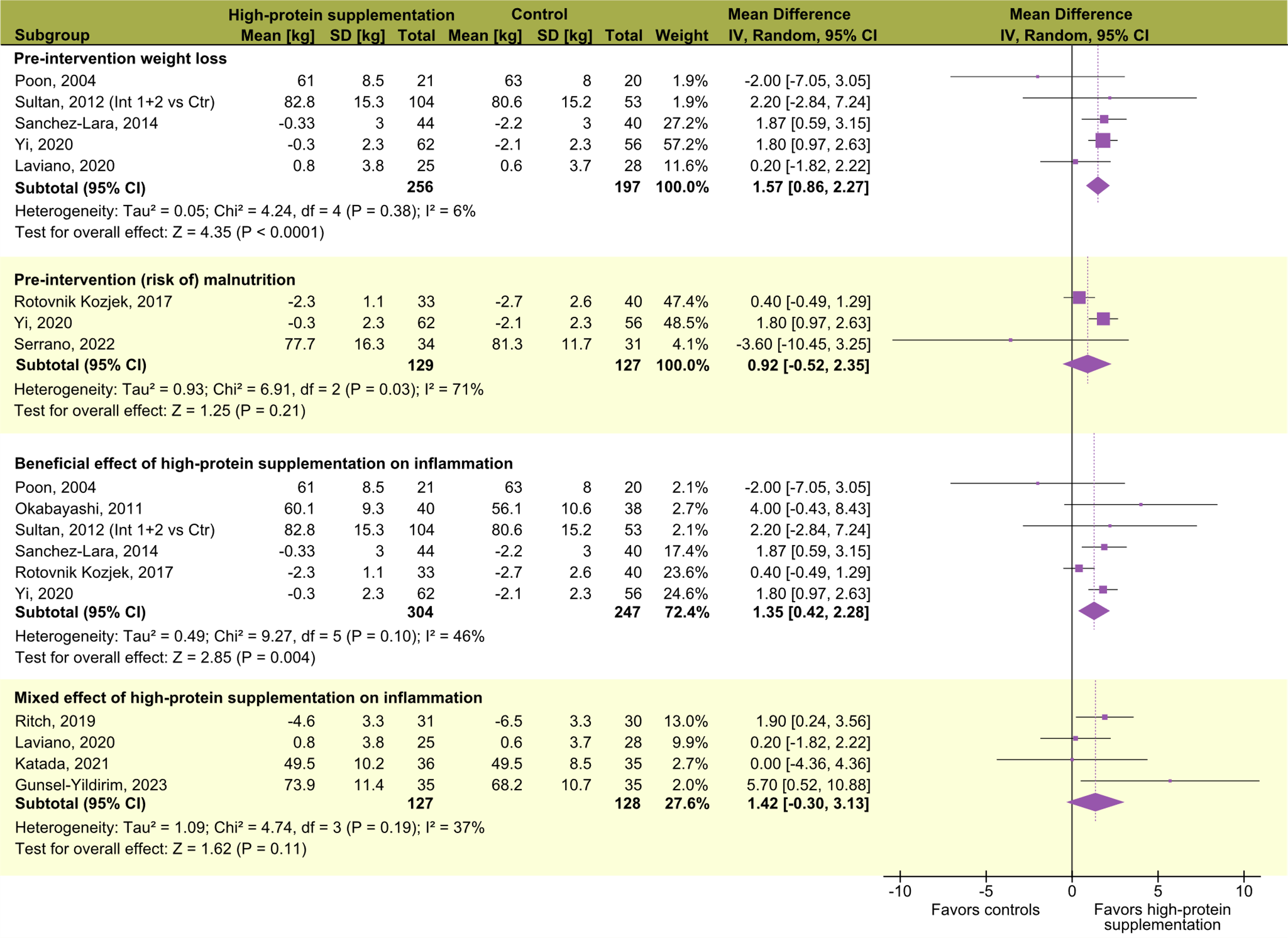
**

## Supplemental Figure 7. Overview of the studies assessing the effects of high-protein supplementation on body composition.

**
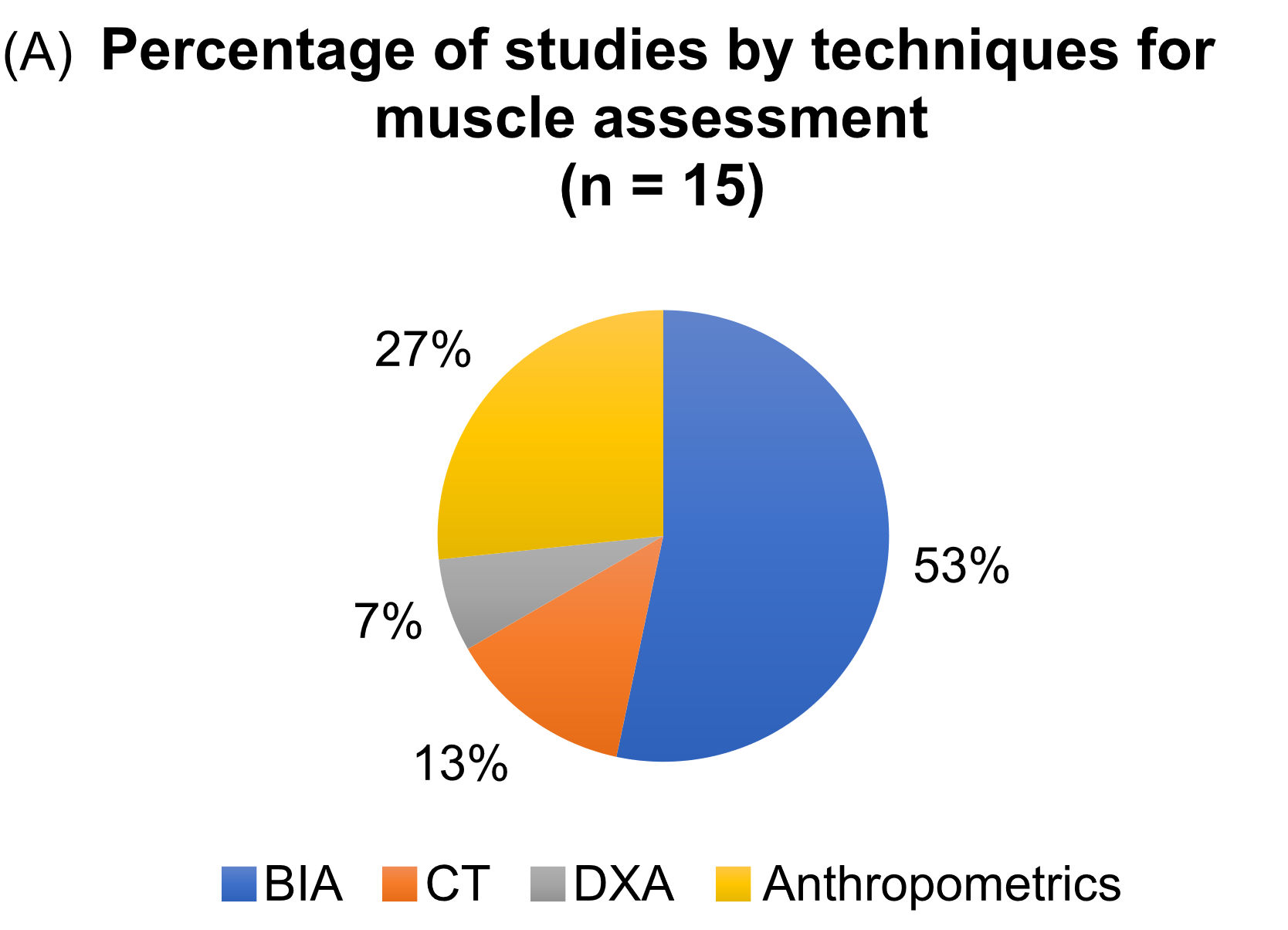

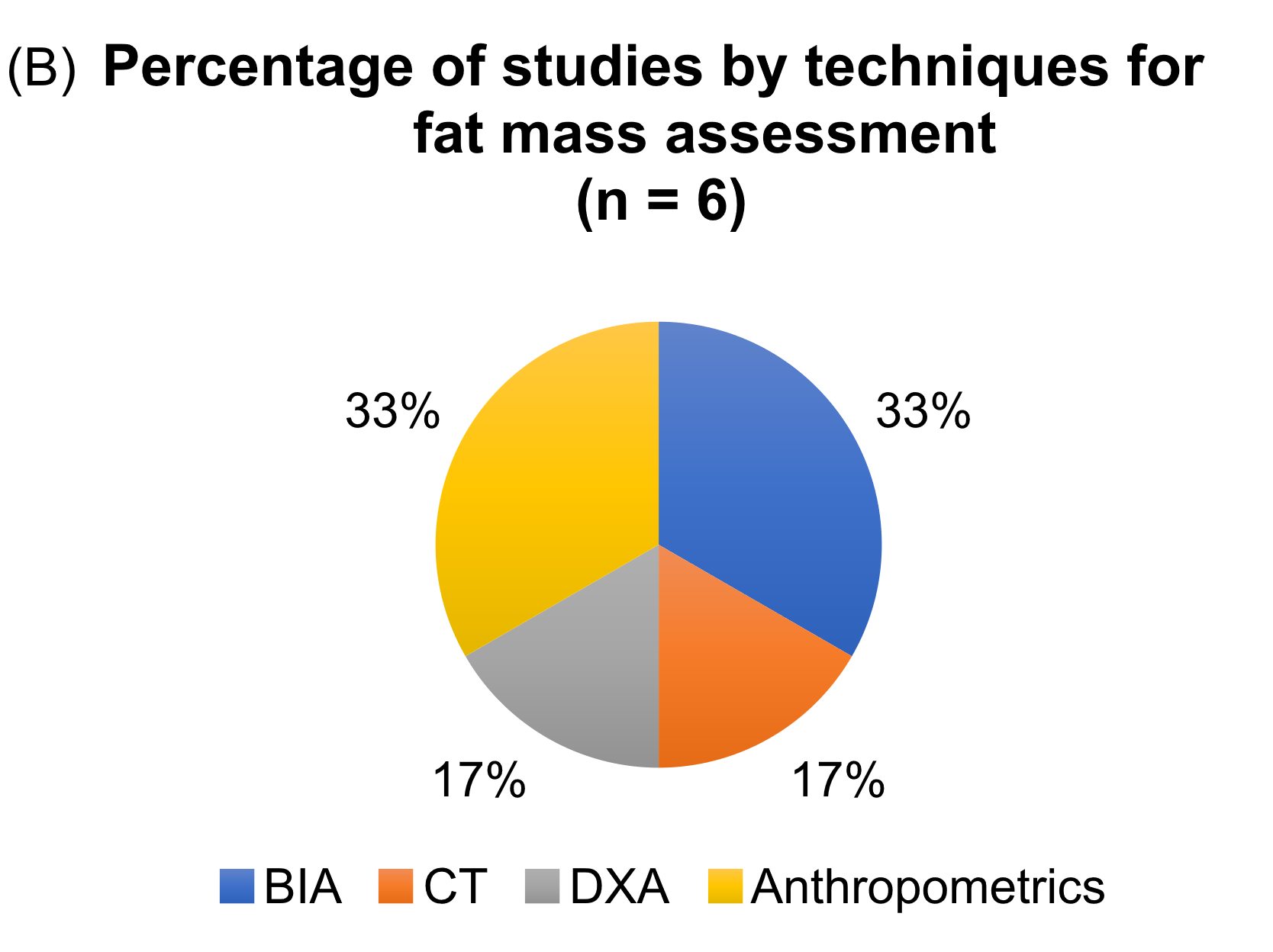
** **
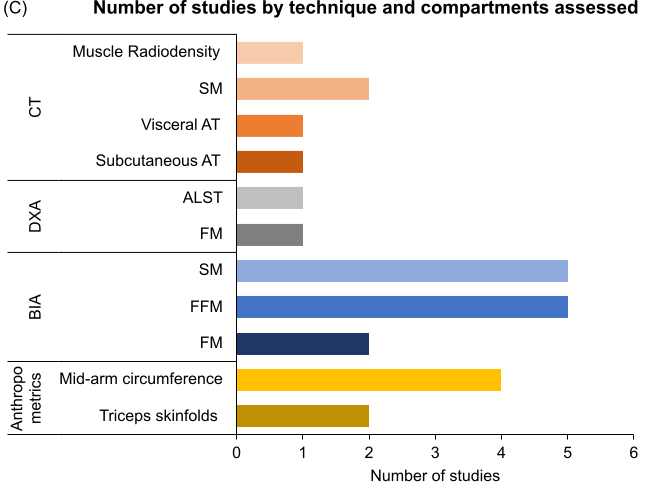
**
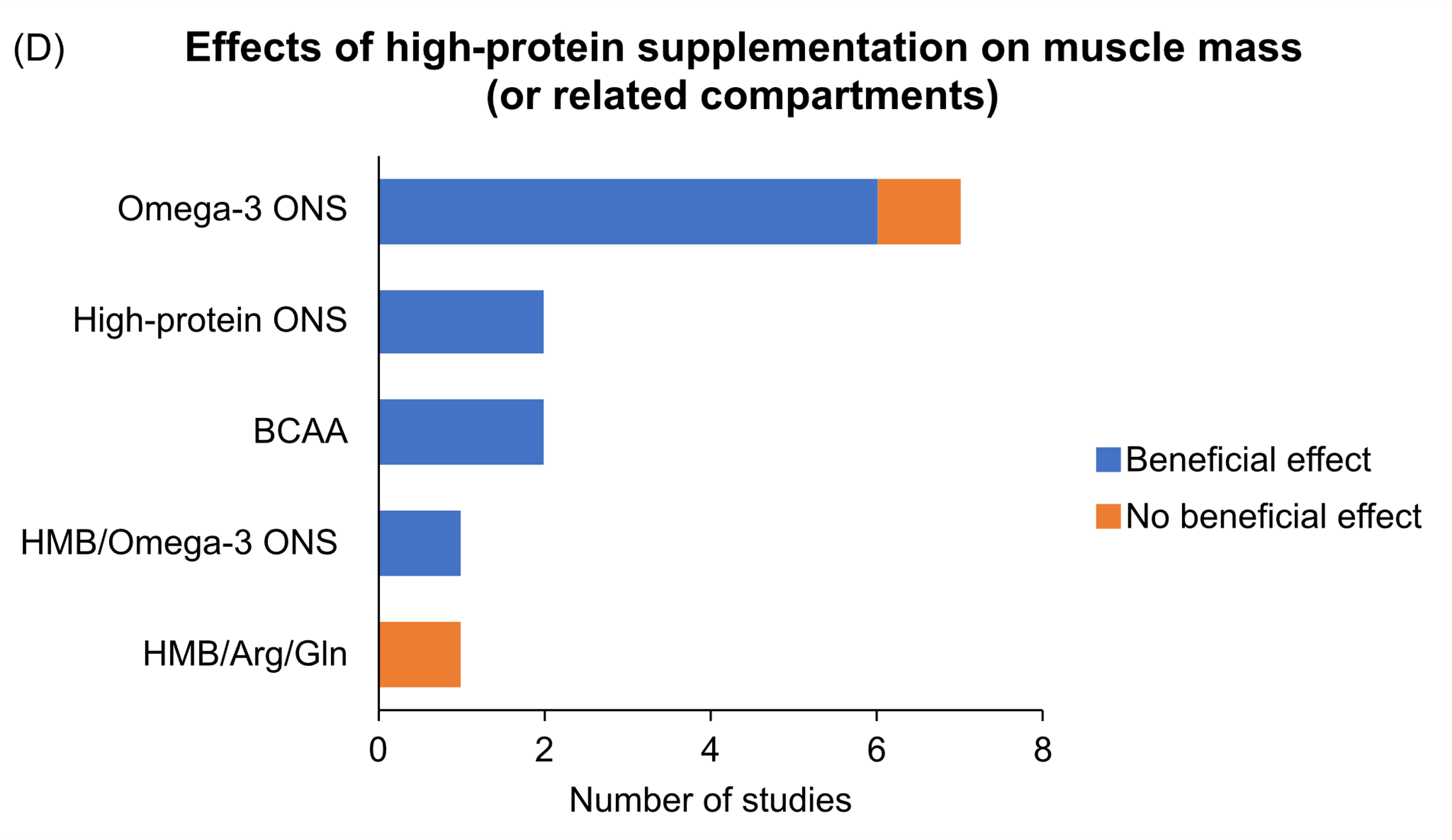


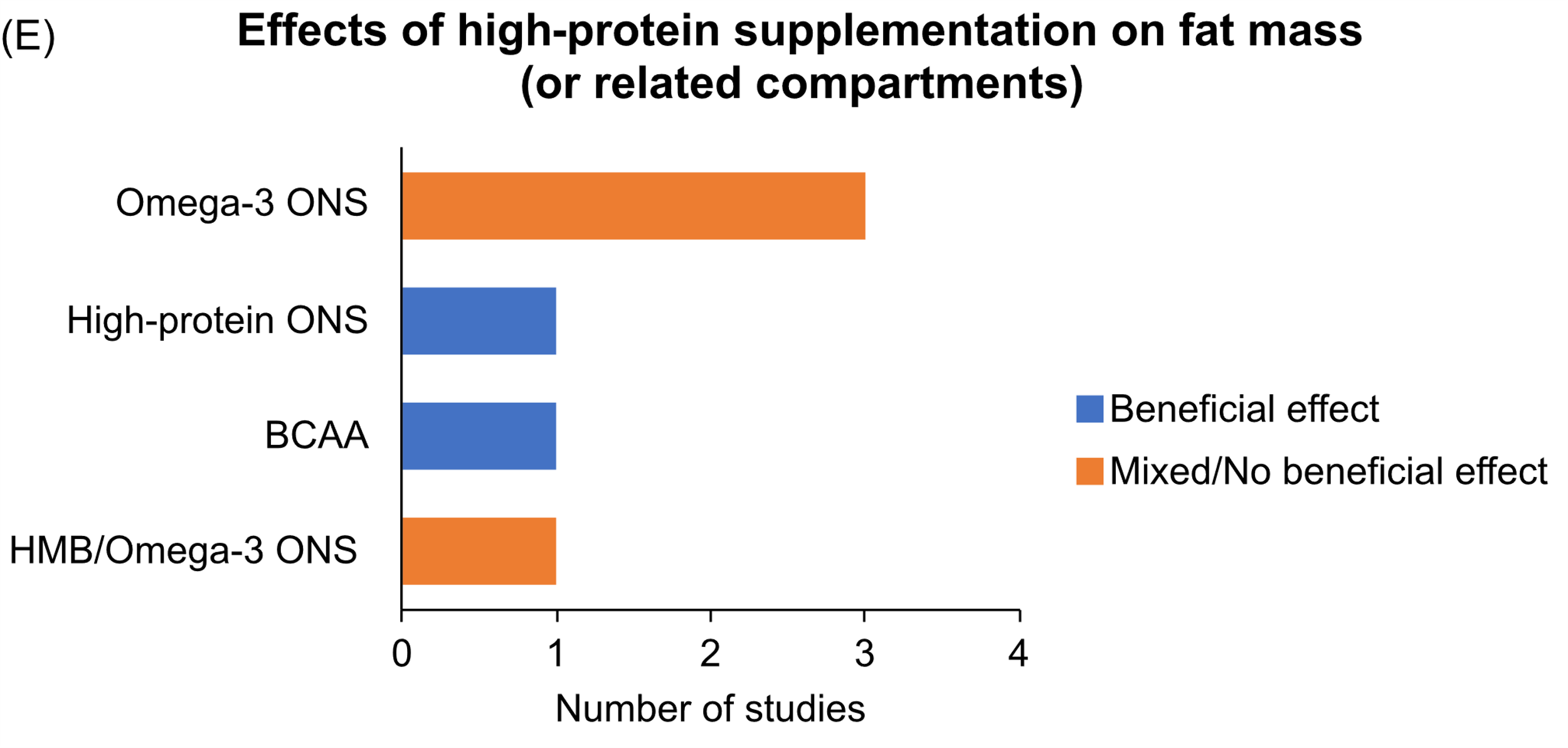


(A) Percentage of studies assessing muscle-related compartments by body composition technique. (B) Percentage of studies assessing fat mass-related compartments by body composition technique. (C) Number of studies by body composition techniques and compartments assessed. (D) Number of studies showing a beneficial effect and no beneficial effect on muscle-related compartments stratified by supplement type. (E) Number of studies showing a beneficial effect and mixed/no beneficial effect on fat mass-related compartments stratified by supplement type. Abbreviations: ALST, appendicular lean soft tissue; Arg, arginine; AT, adipose tissue; BCAA, branched-chain amino acids; BIA, bioelectrical impedance analysis; CT, computed tomography; DXA, dual energy x-ray absorptiometry; FFM, fat-free mass; FM, fat mass; Gln, glutamine; HMB, *β*-hydroxy *β*-methylbutyrate; ONS, oral nutritional supplement; SM, skeletal muscle.

## Supplemental Figure 8. Changes in muscle-related measures from baseline to follow-up within high-protein supplementation (green bar) and control (blue bar) groups.


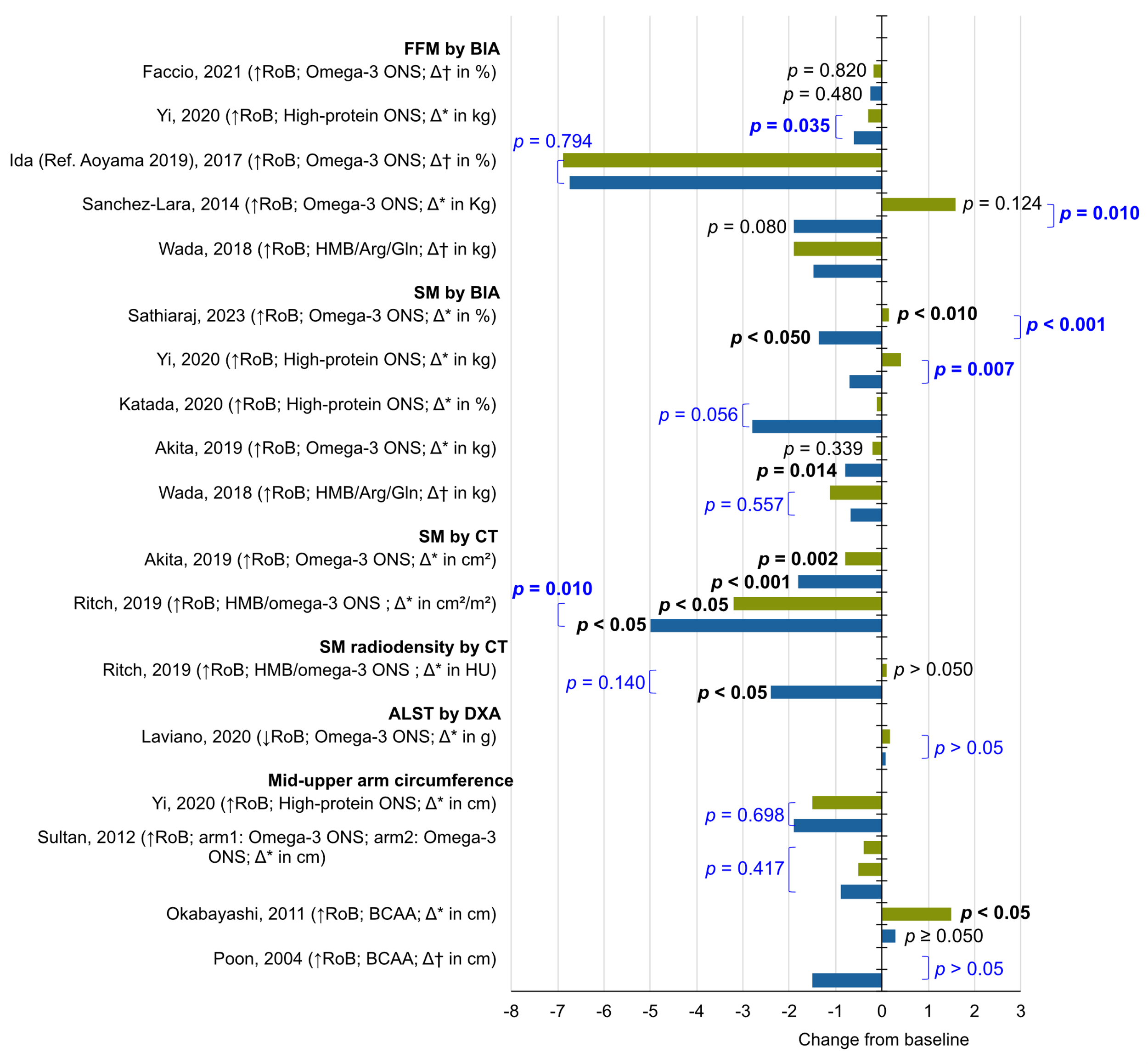
 Δ* and Δ† represent absolute or percent mean and median change, respectively. P-value in black corresponds to testing of differences from baseline to follow-up within each group. P-value in blue represents testing of differences between experimental and control groups. Bolded p-values are statistically significant. Abbreviations: ALST, appendicular lean soft tissue; Arg, arginine; BCAA, branched-chain amino acids; BIA, bioelectrical impedance analysis; CT, computed tomography; DXA, dual energy x-ray absorptiometry; FFM, fat-free mass; Gln, glutamine; HMB, β-hydroxy β-methylbutyrate; ONS, oral nutritional supplement; ↓RoB, low risk of bias; ↔ RoB, moderate risk of bias; ↑ RoB, high risk of bias; SM, skeletal muscle.

## Supplemental Figure 9. Changes in fat mass (and related measures) from baseline to follow-up within high-protein supplementation (green bar) and control (blue bar) groups.


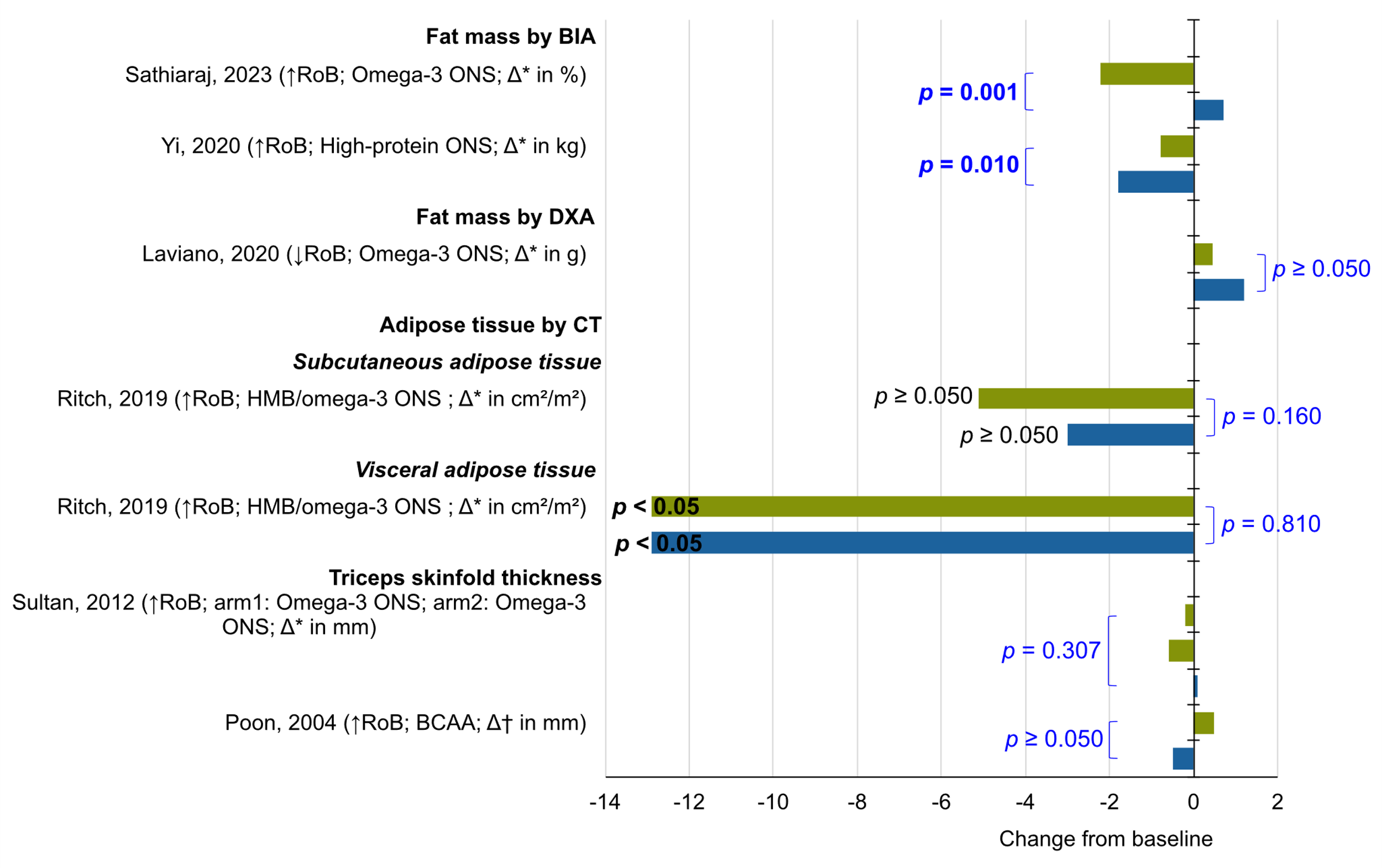


Δ* and Δ† represent absolute or percent mean and median change, respectively. *P*-value in black corresponds to testing of differences from baseline to follow-up within each group. *P*-value in blue represents testing of differences between experimental and control groups. Bolded p-values are statistically significant. Abbreviations: BCAA, branched-chain amino acids; BIA, bioelectrical impedance analysis; CT, computed tomography; DXA, dual energy x-ray absorptiometry; HMB, *β*-hydroxy *β*-methylbutyrate; ONS, oral nutritional supplement; ↓RoB, low risk of bias; ↔ RoB, moderate risk of bias; ↑ RoB, high risk of bias.

## Supplemental Figure 10. Changes in measures of muscle function and performance from baseline to follow-up within high-protein supplementation (green bar) and control (blue bar) groups. Gray bar represents differences between high-protein supplementation and control groups at follow up.


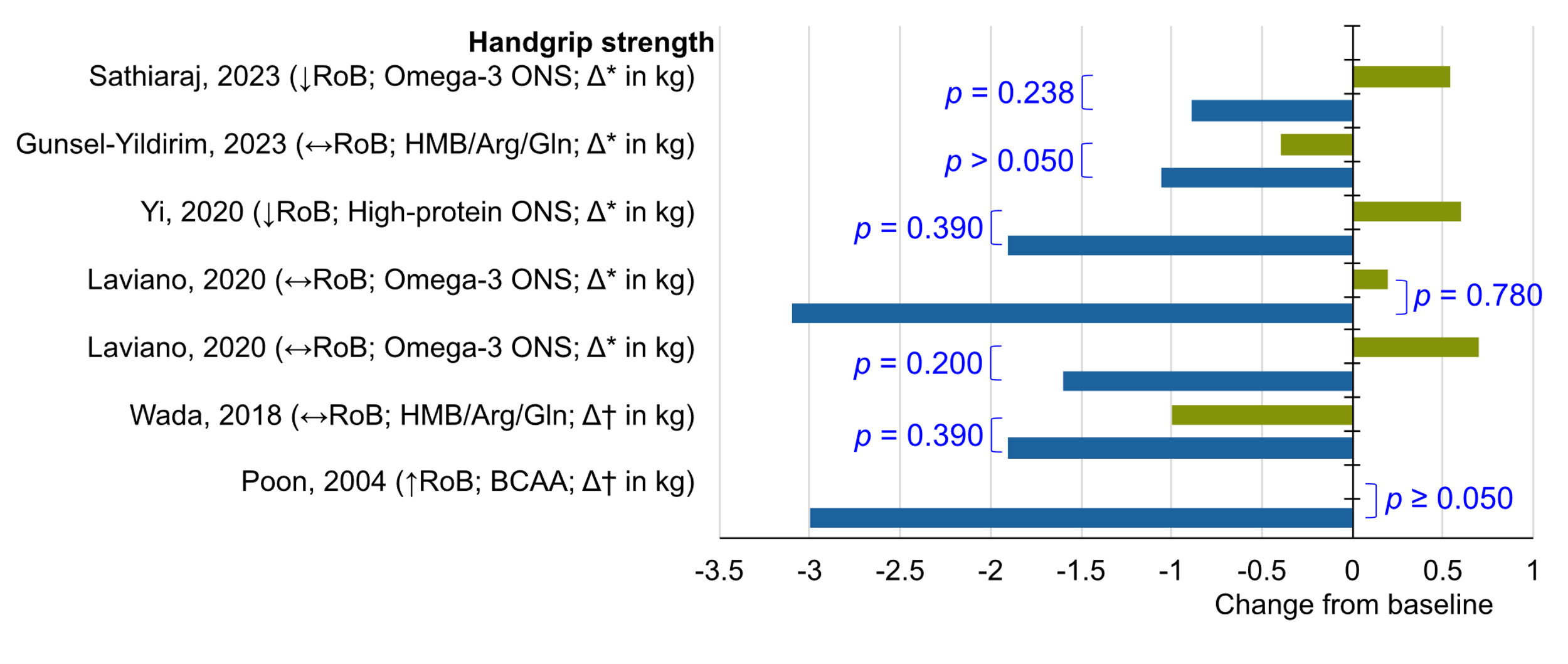


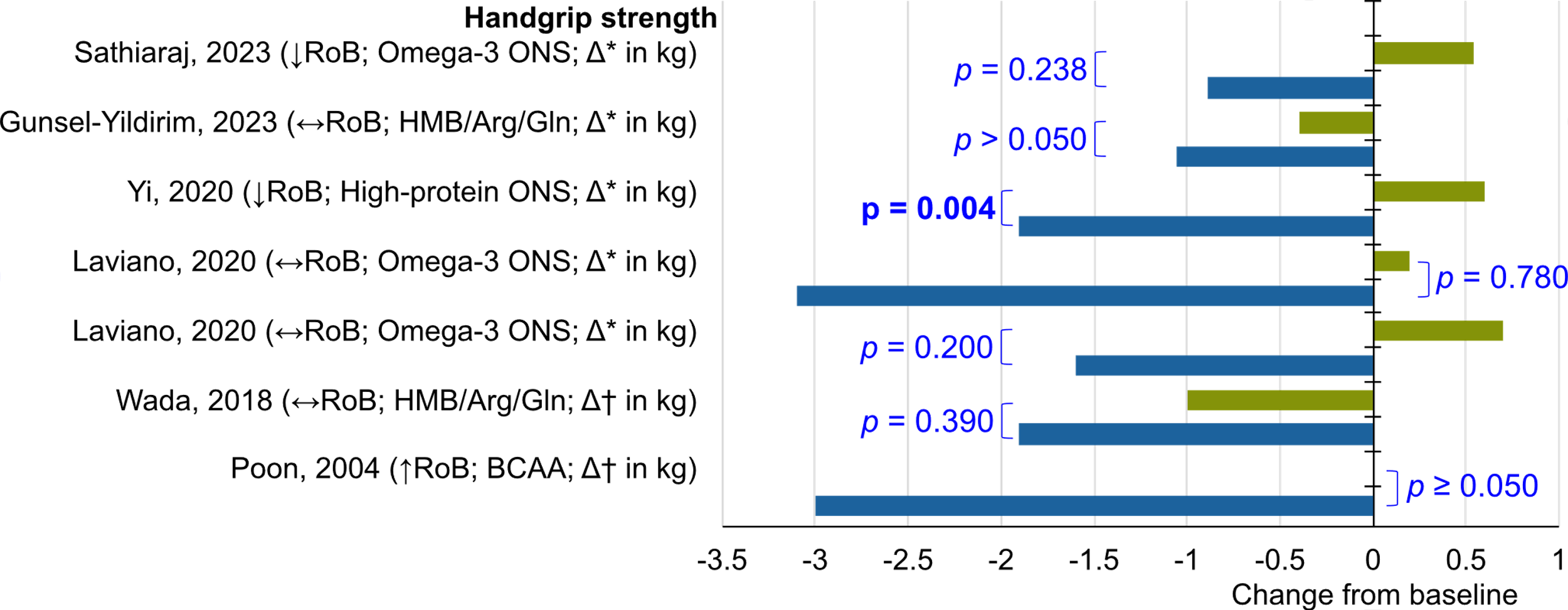

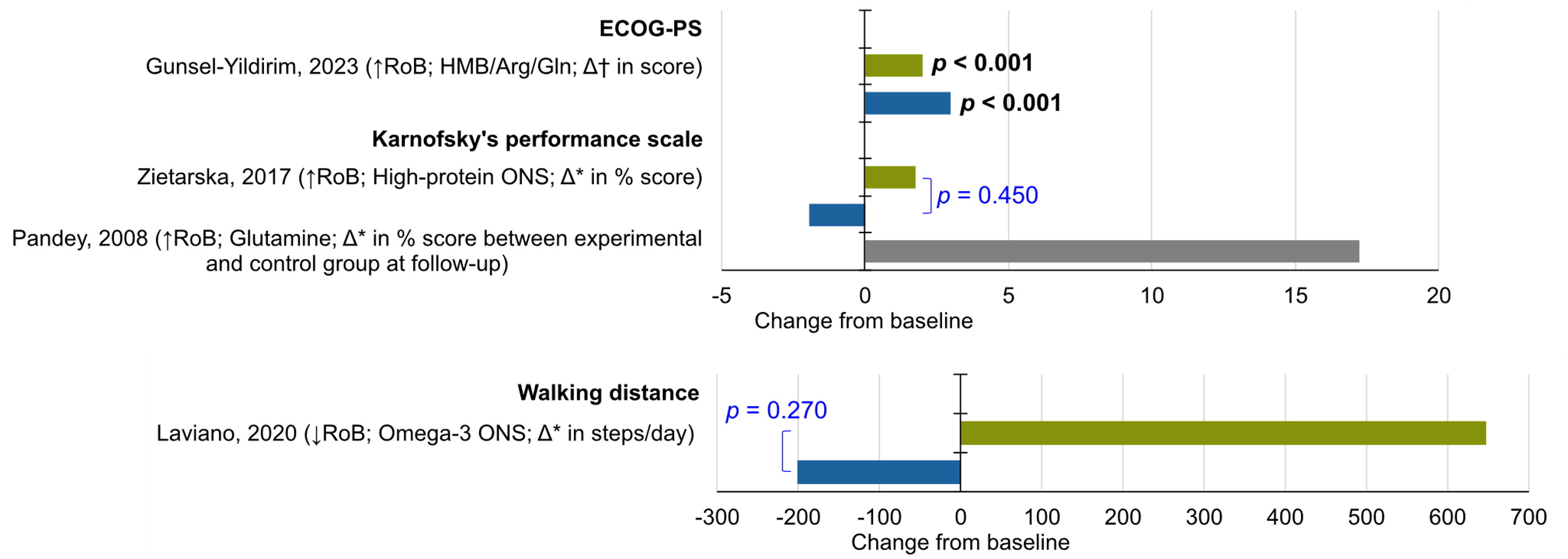


Δ* and Δ† represent absolute or percent mean and median change, respectively. *P*-value in black corresponds to testing of differences from baseline to follow-up within each group. *P*-value in blue represents testing of differences between experimental and control groups. Bolded p-values are statistically significant. Abbreviations: Arg, arginine; BCAA, branched-chain amino acids; ECOG-PS, Eastern Cooperative Oncology Group Performance Status; Gln, glutamine; HMB, *β*-hydroxy *β*-methylbutyrate; ONS, oral nutritional supplement; ↓RoB, low risk of bias; ↔ RoB, moderate risk of bias; ↑ RoB, high risk of bias.

## Supplemental Figure 11. Survival outcomes at follow-up within high-protein supplementation (green bar) and control (blue bar) groups**.**


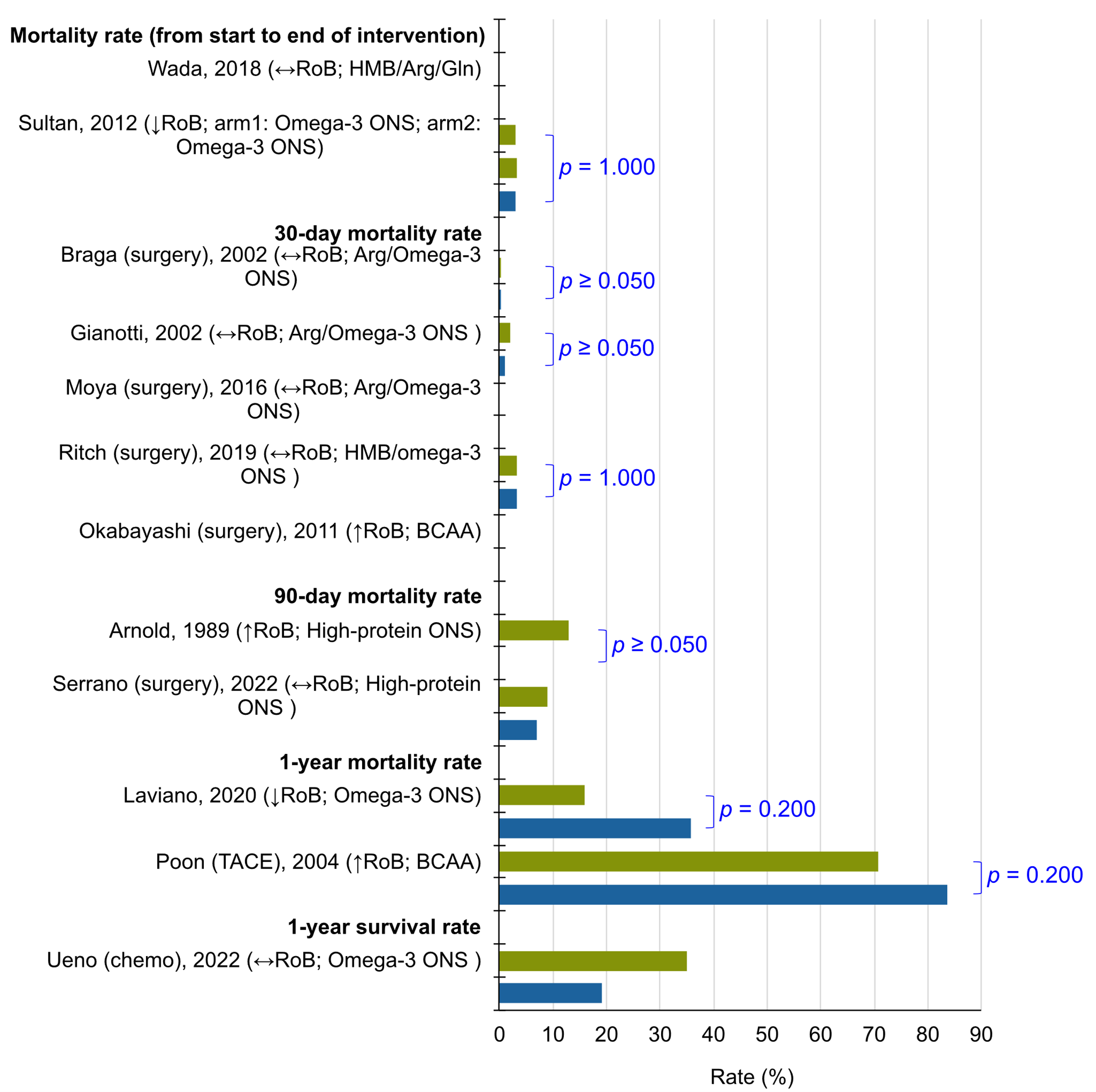

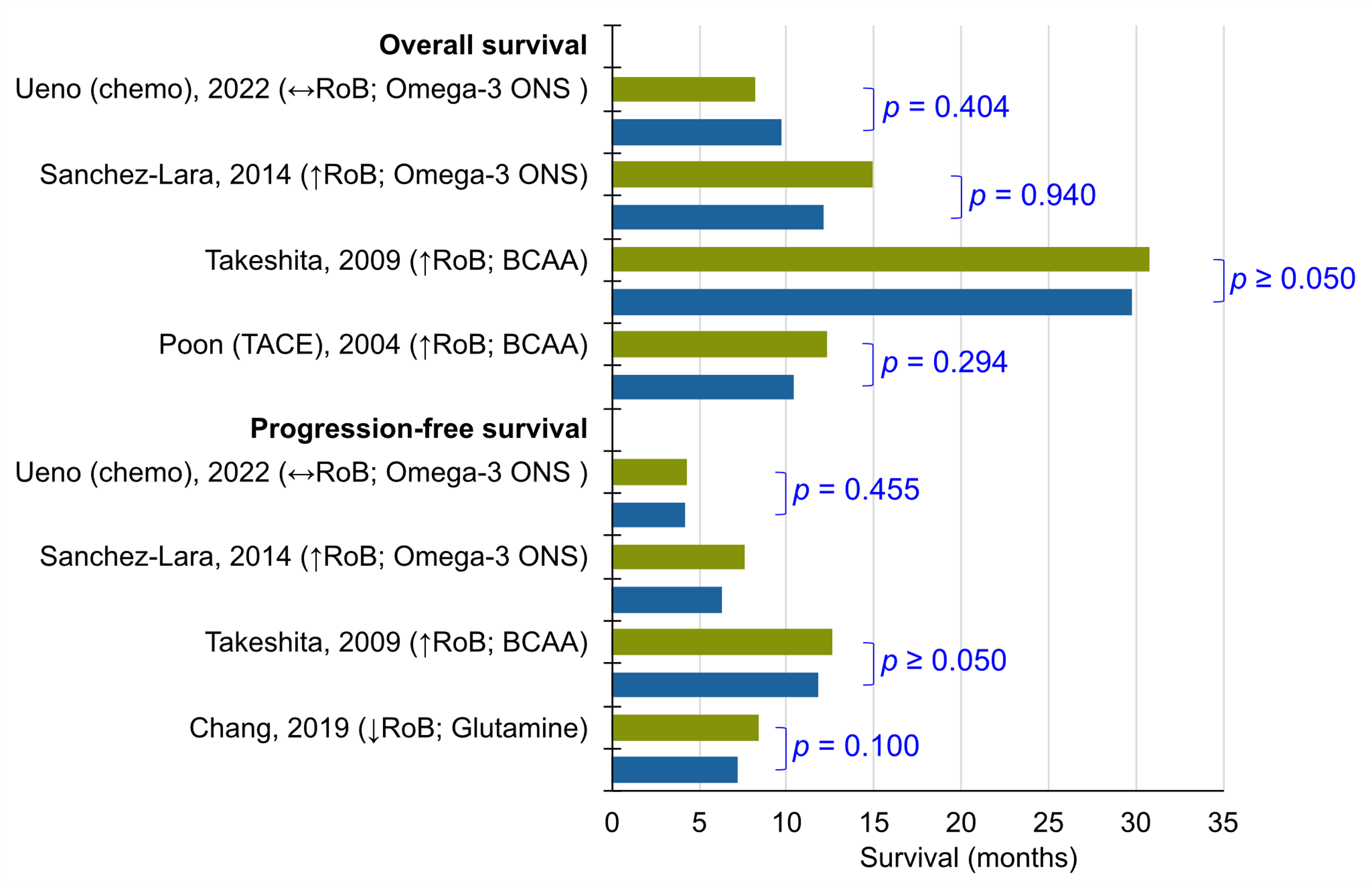


P-value in blue represents testing of differences between experimental and control groups. Bolded p-values are statistically significant. Studies with data not shown reported zero deaths during the period described in the graph. Abbreviations: Arg, arginine; BCAA, branched-chain amino acids; Gln, glutamine; HMB, β-hydroxy β-methylbutyrate; ONS, oral nutritional supplement; ↓RoB, low risk of bias; ↔ RoB, moderate risk of bias; ↑ RoB, high risk of bias.

## Supplemental Figure 12. Complications at follow-up within high-protein supplementation (green bar) and control (blue bar) groups.


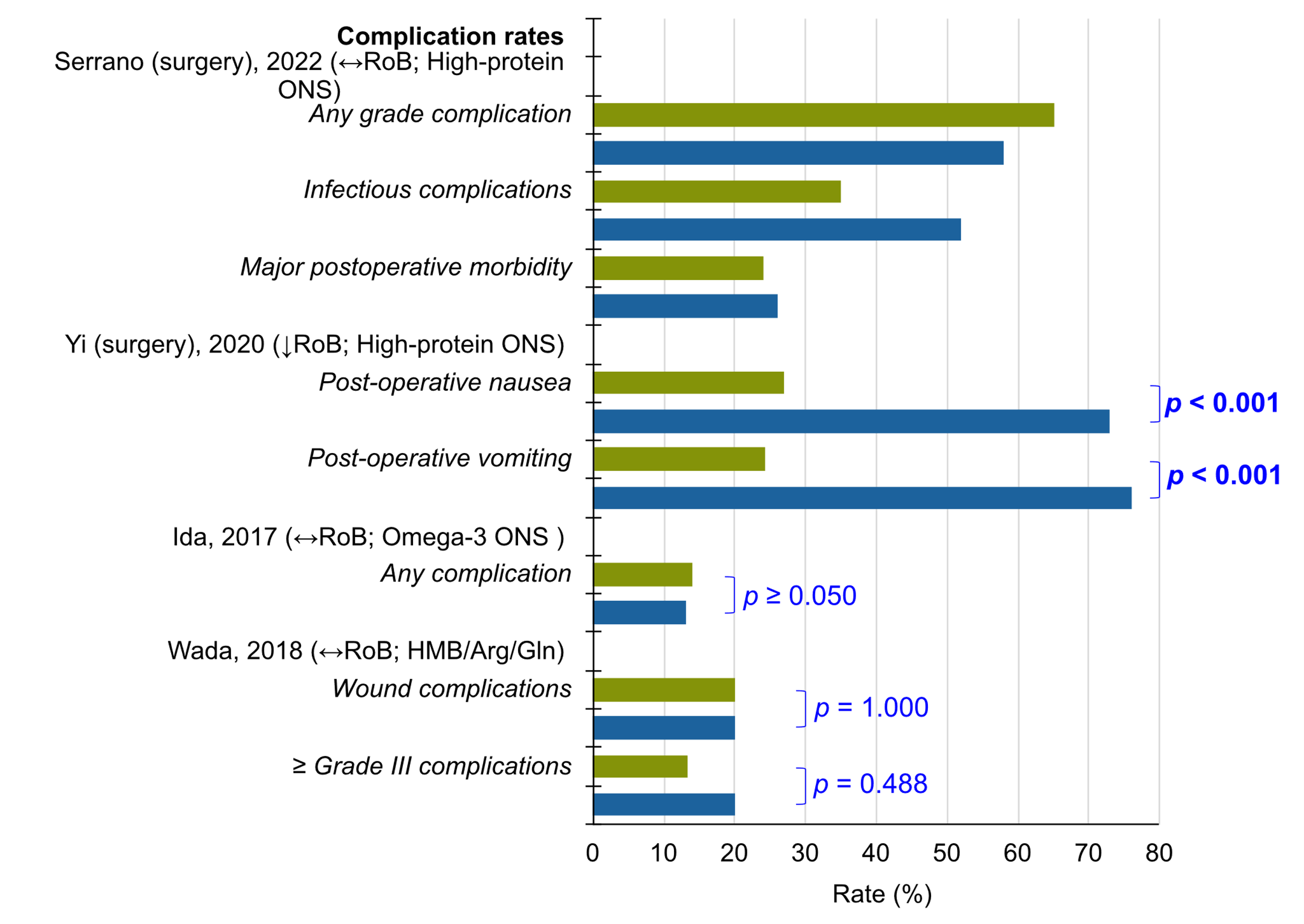

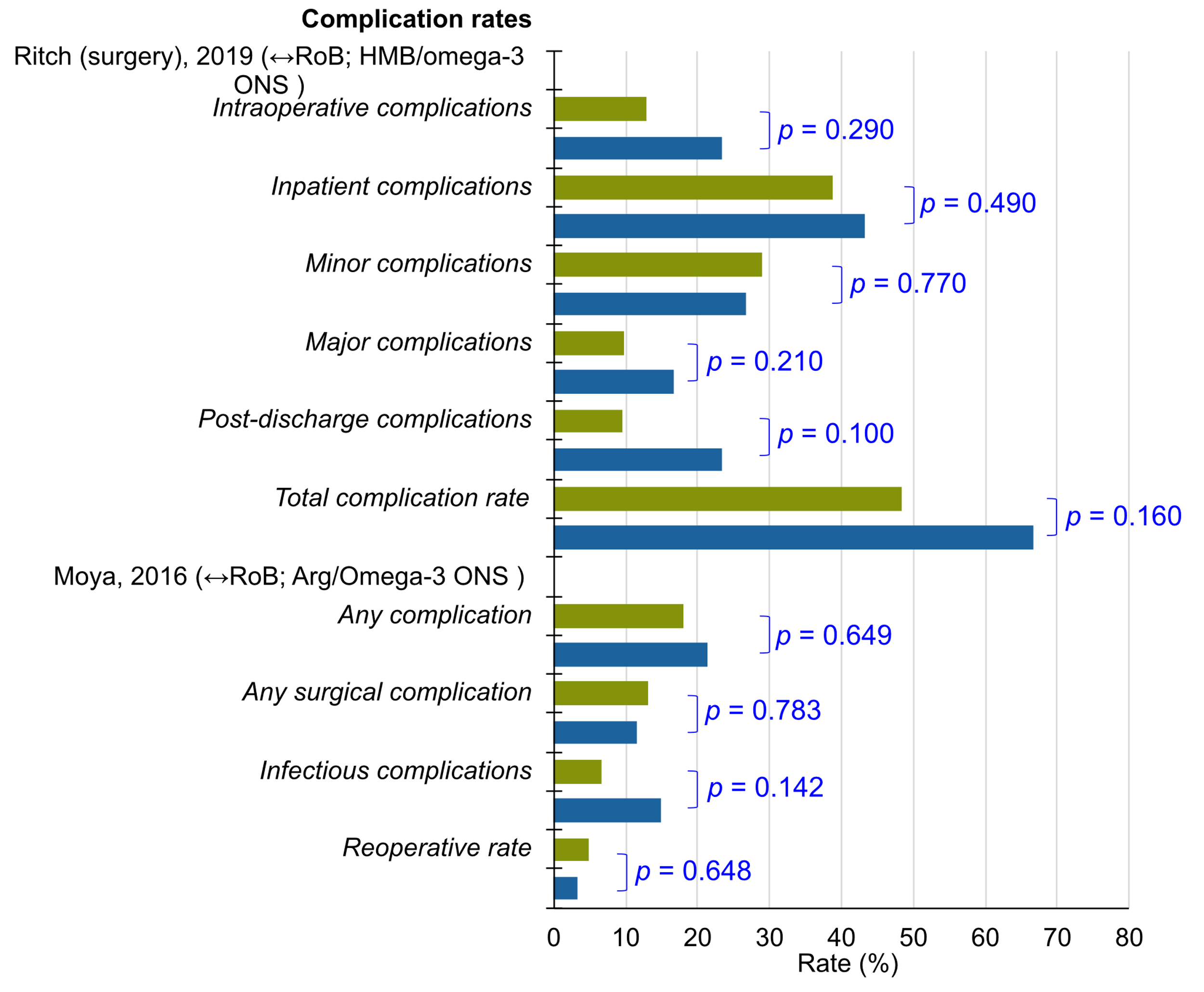

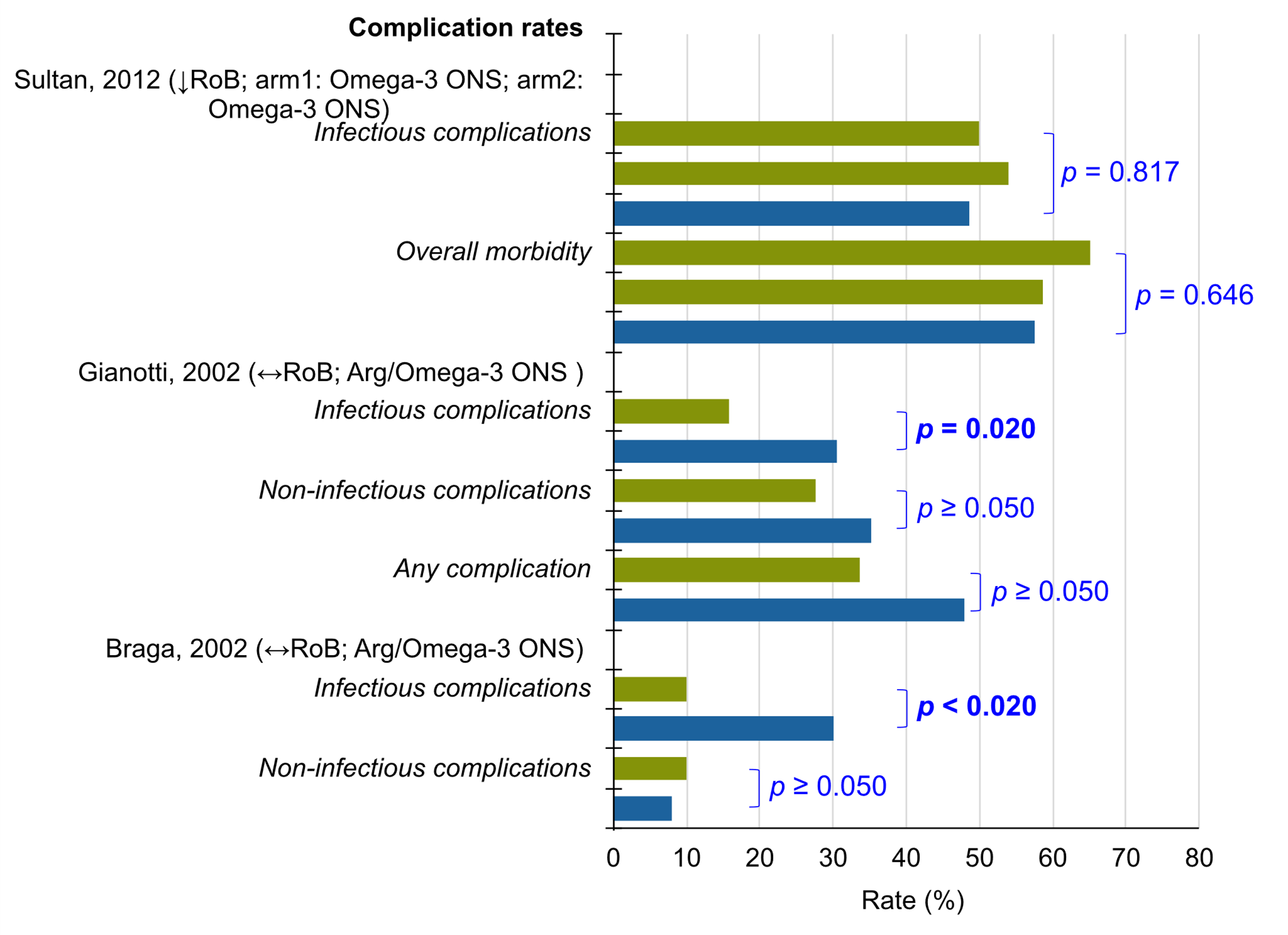

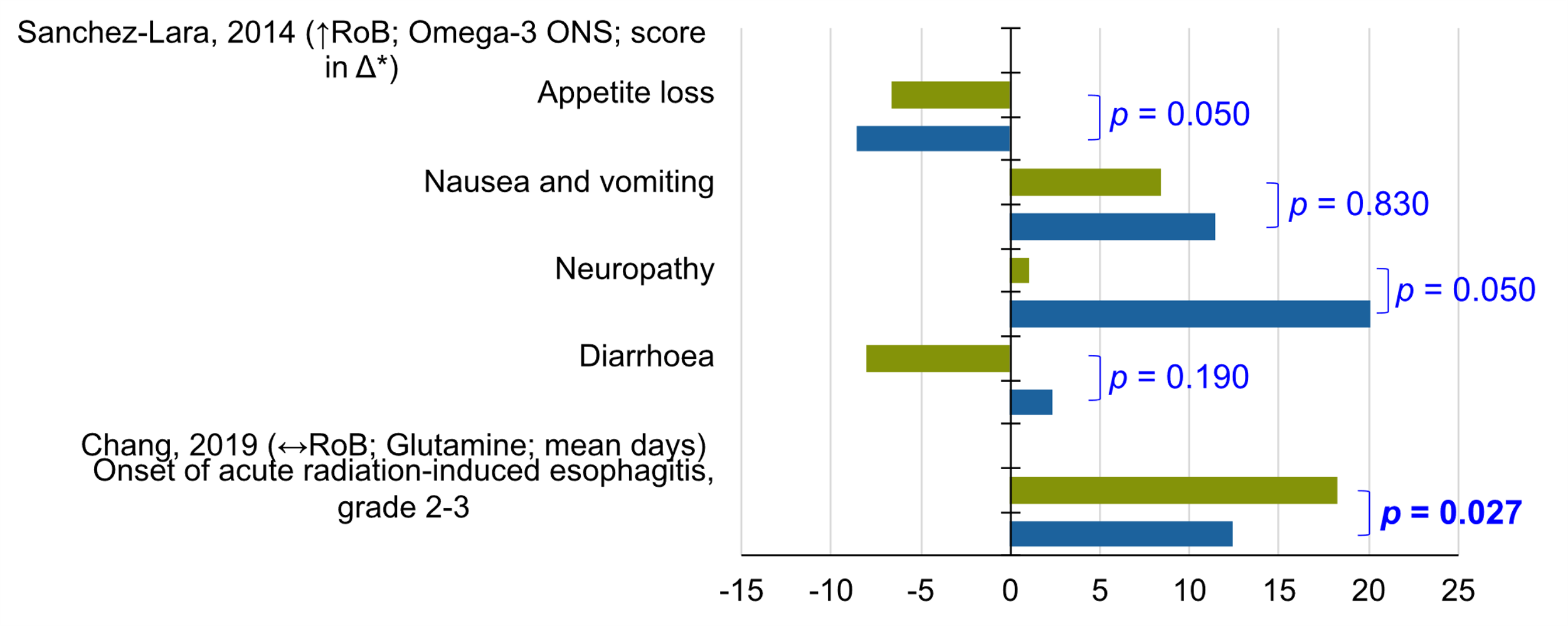


Δ* represents absolute mean change from baseline, respectively. *P*-value in blue represents testing of differences between experimental and control groups. Bolded p-values are statistically significant. Abbreviations: Arg, arginine; BCAA, branched-chain amino acids; Gln, glutamine; HMB, *β*-hydroxy *β*-methylbutyrate; ONS, oral nutritional supplement; ↓RoB, low risk of bias; ↔ RoB, moderate risk of bias; ↑ RoB, high risk of bias.

## Supplemental Figure 13. Length of stay at follow-up within high-protein supplementation (green bar) and control (blue bar) groups.


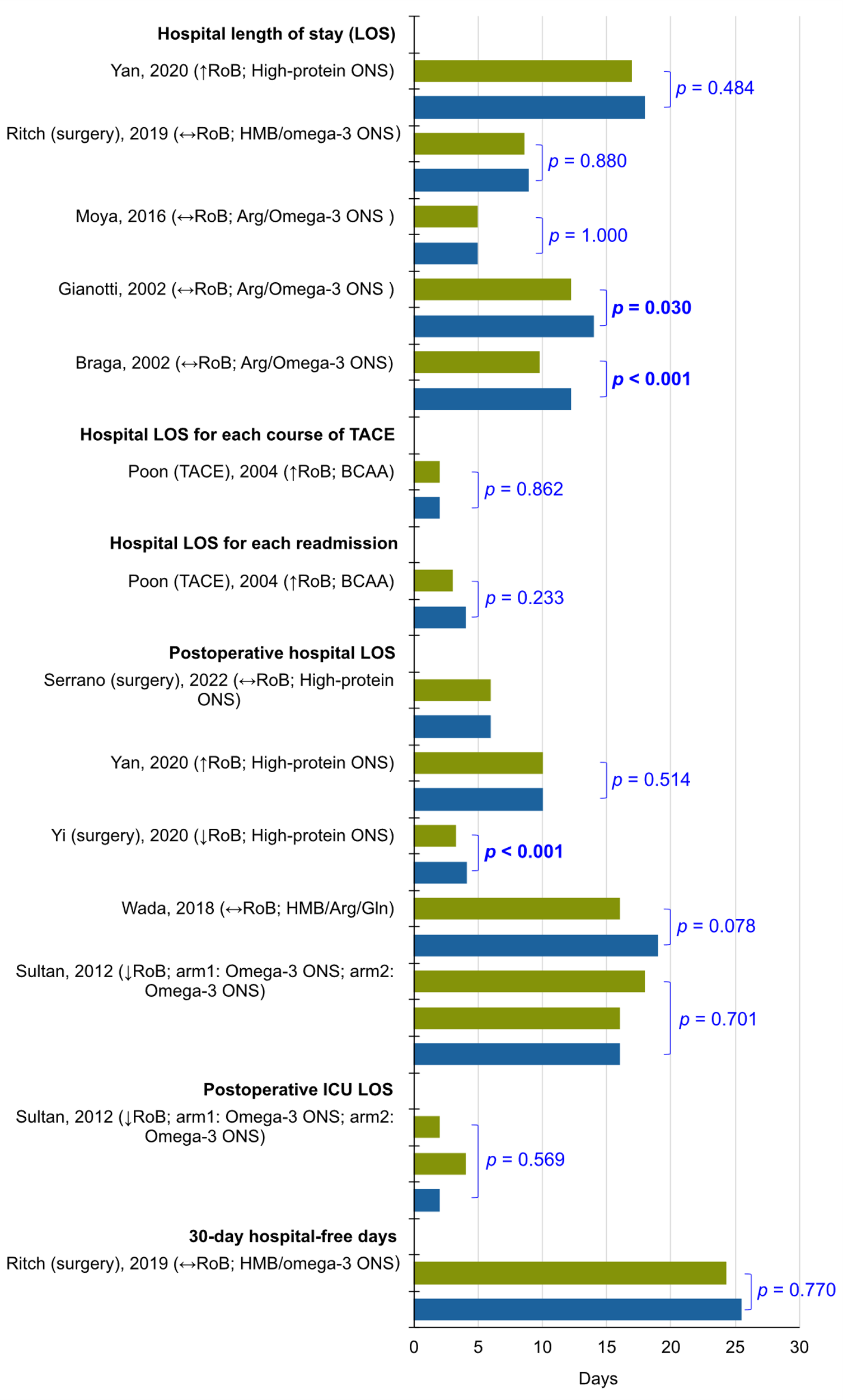


*P*-value in blue represents testing of differences between experimental and control groups. Bolded p-values are statistically significant. Abbreviations: Arg, arginine; BCAA, branched-chain amino acids; BCAA, branched-chain amino acids; ICU:  intensive care unit Gln, glutamine; HMB, *β*-hydroxy *β*-methylbutyrate; LOS: Length of stay;  ONS, oral nutritional supplement; ↓RoB, low risk of bias; ↔ RoB, moderate risk of bias; ↑ RoB, high risk of bias; TACE, transarterial chemoembolization.

## Supplemental Figure 14. Hospital admission or readmission rates and unplanned emergency room visits at follow-up within high-protein supplementation (green bar) and control (blue bar) groups.


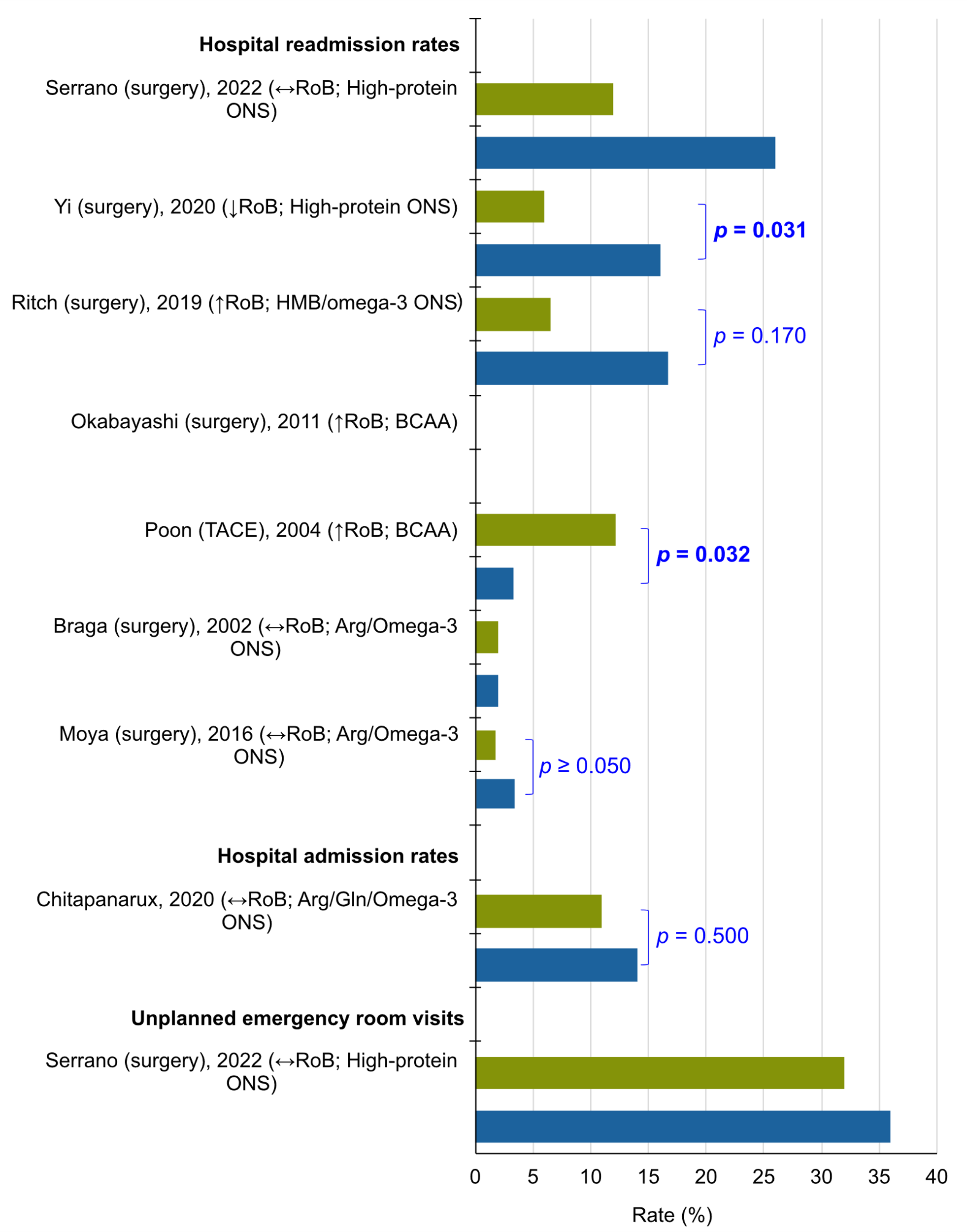


*P*-value in blue represents testing of differences between experimental and control groups. Bolded p-values are statistically significant. Abbreviations: Arg, arginine; BCAA, branched-chain amino acids; Gln, glutamine; HMB, *β*-hydroxy *β*-methylbutyrate; ONS, oral nutritional supplement; ↓RoB, low risk of bias; ↔ RoB, moderate risk of bias; ↑ RoB, high risk of bias; TACE, transarterial chemoembolization.

## Supplemental Figure 15. Incidence of cancer therapy-induced toxicities at follow-up within high-protein supplementation (green bar) and control (blue bar) groups.


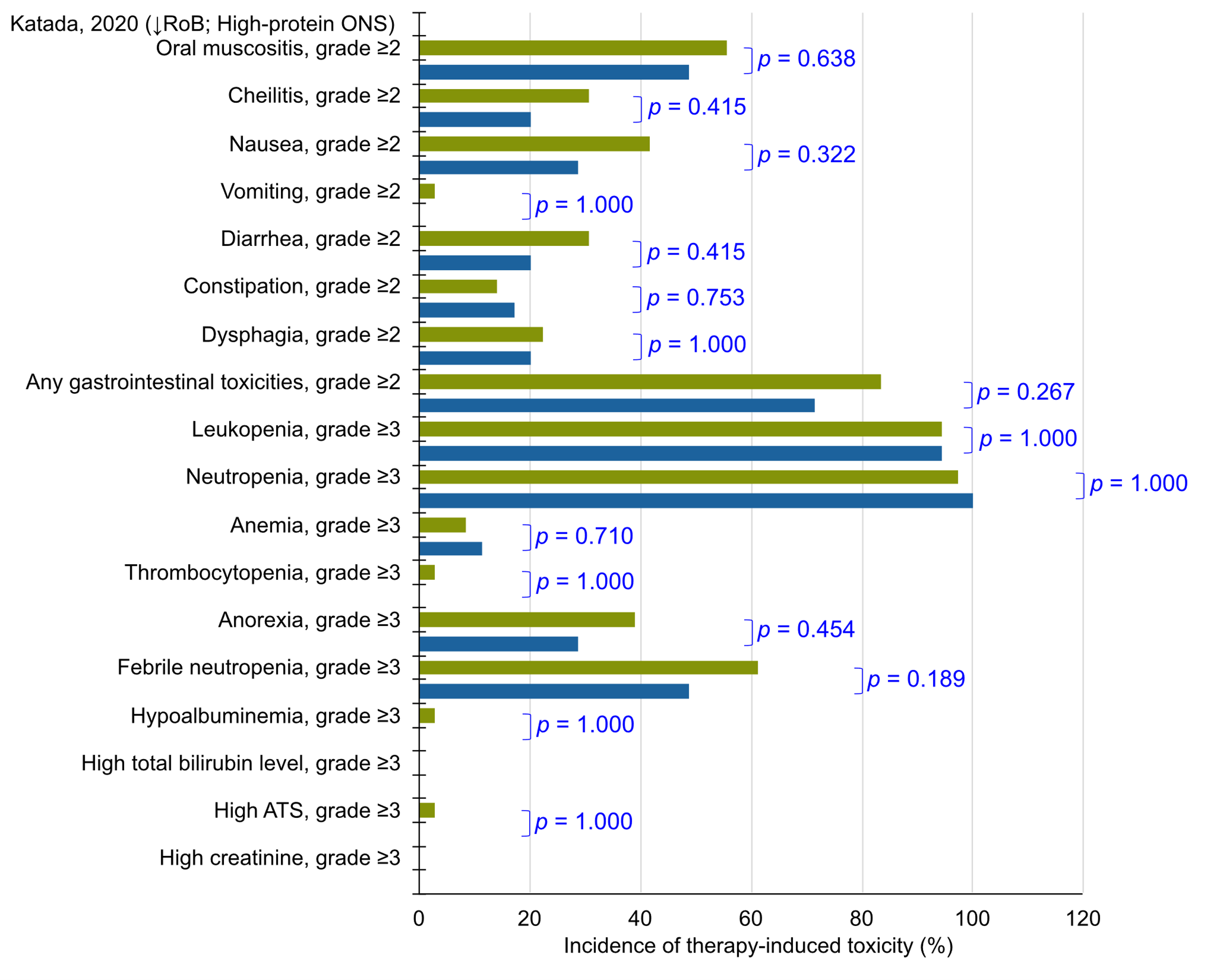

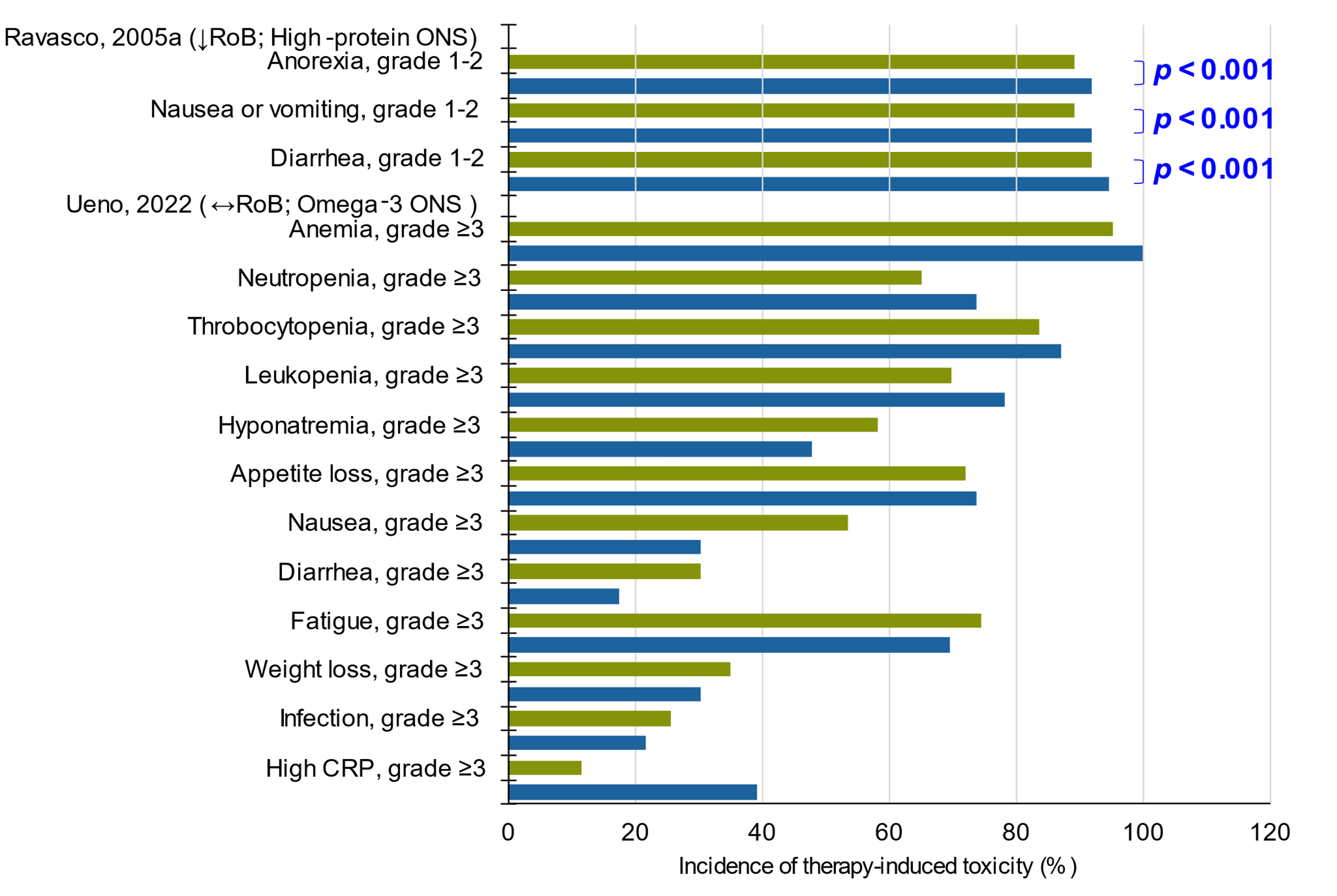

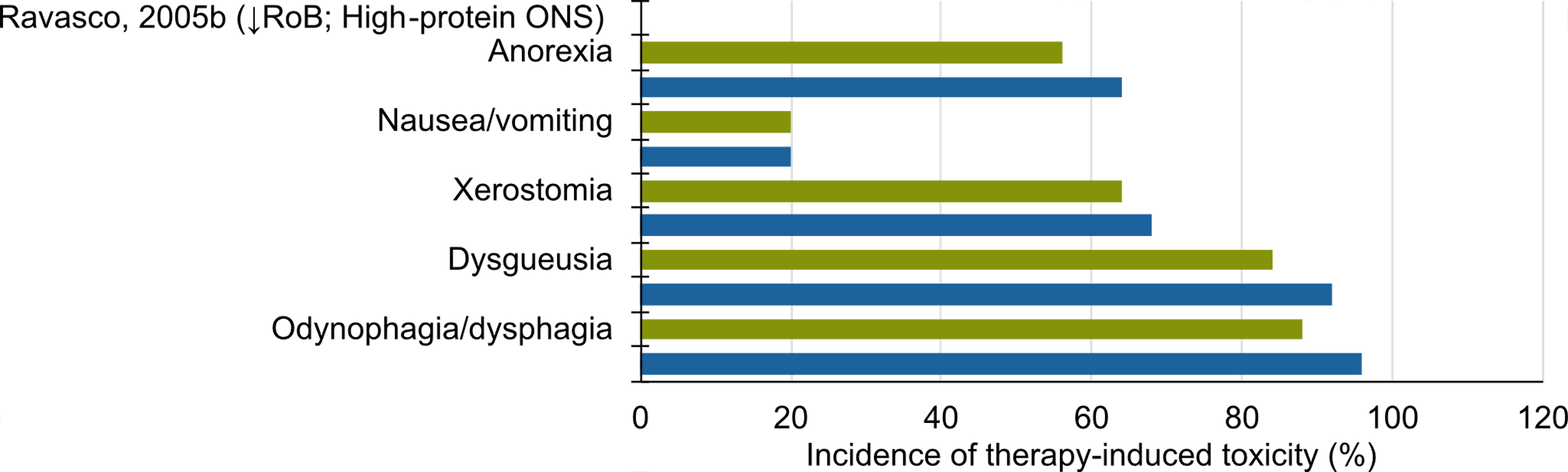

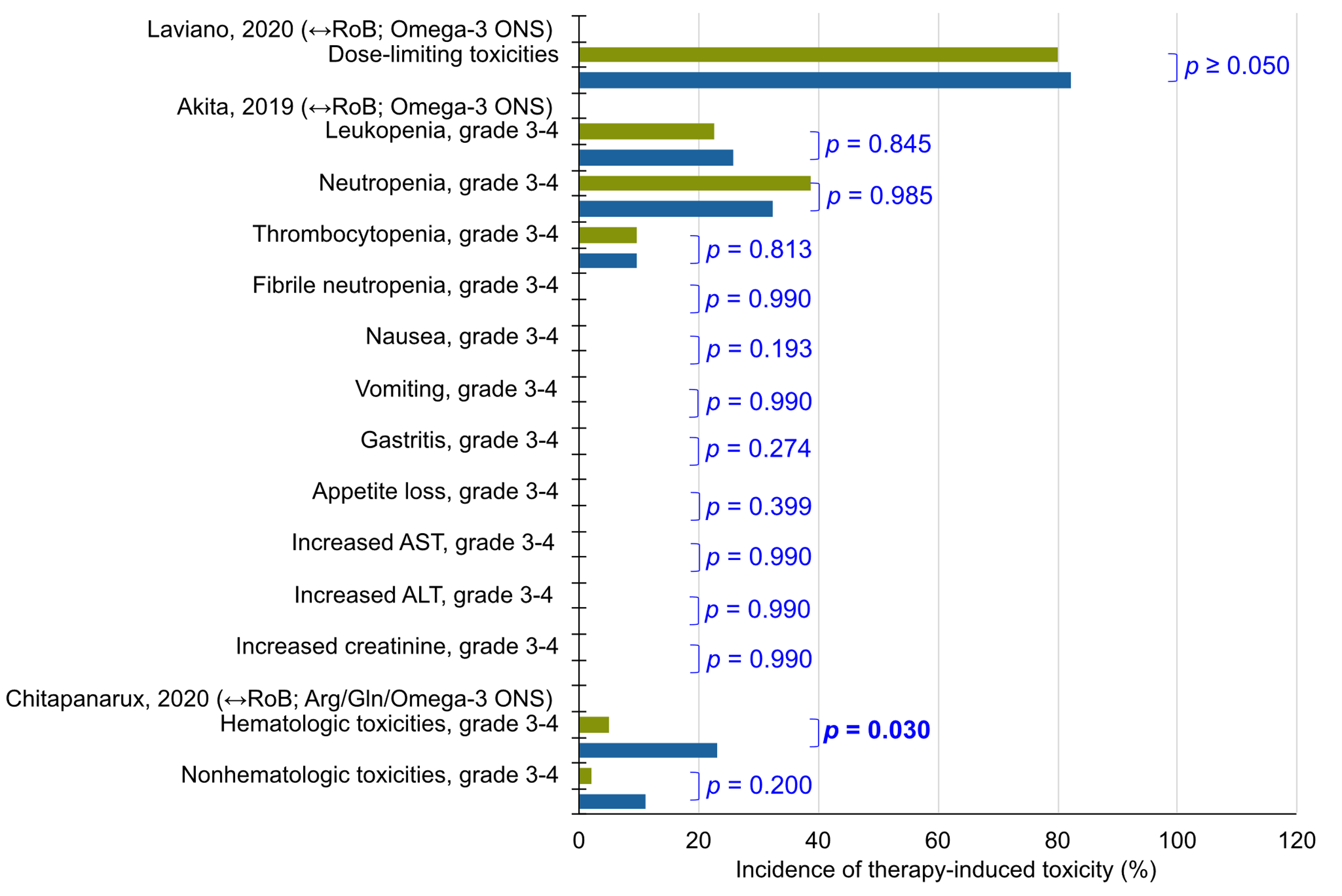

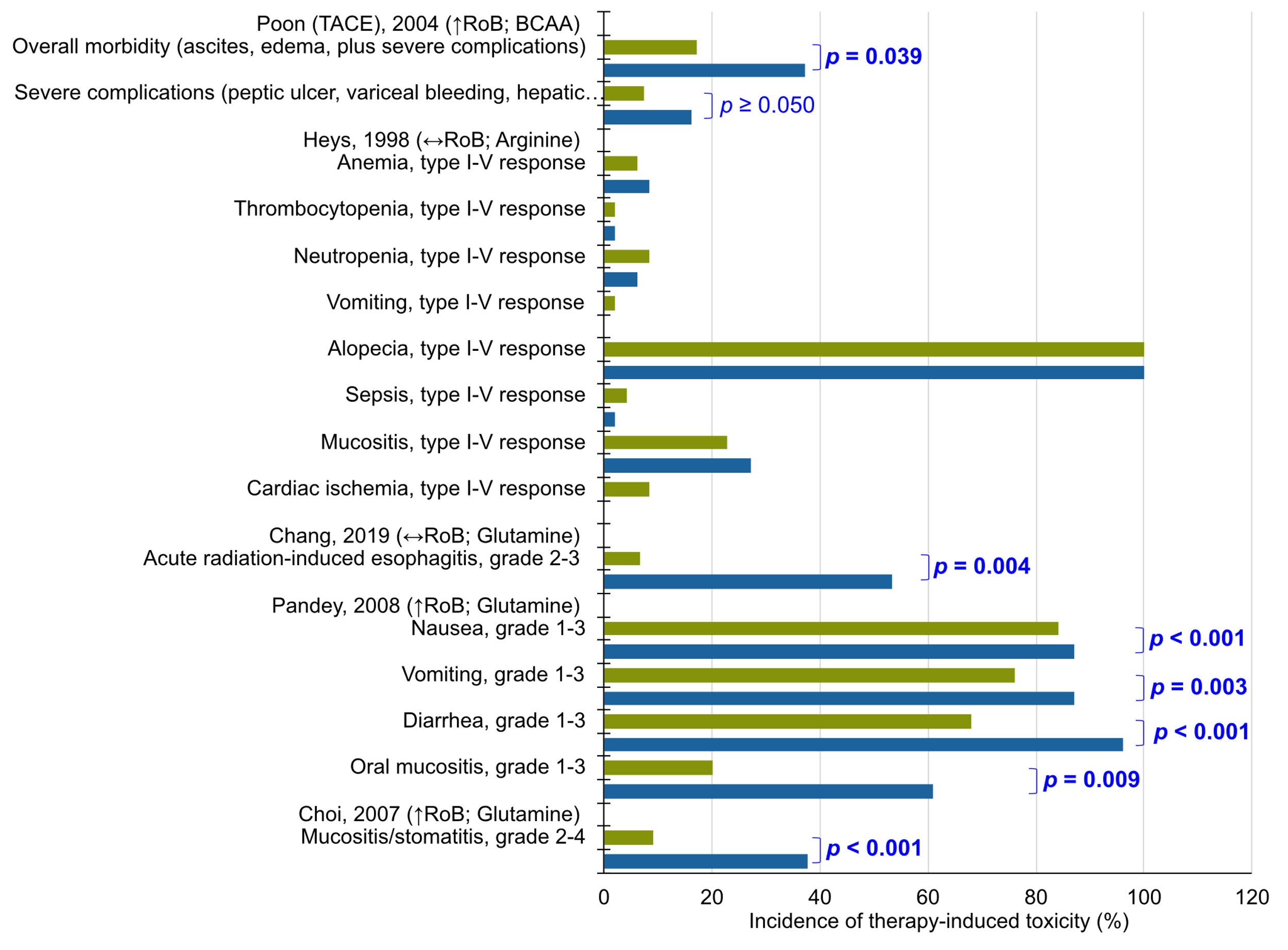


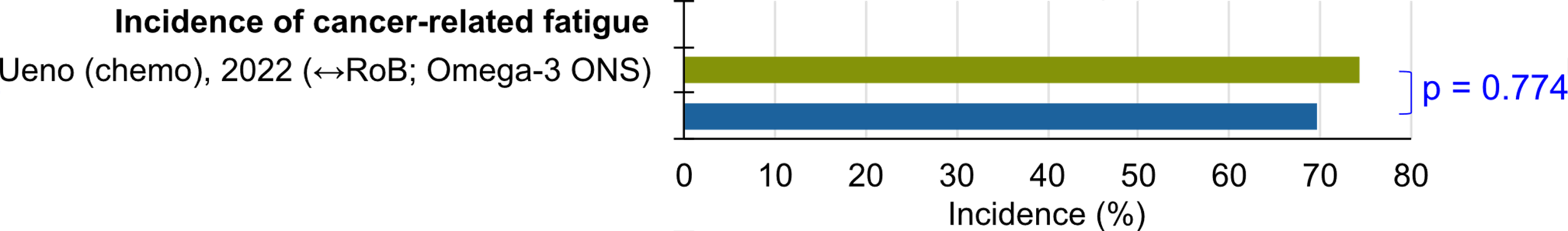

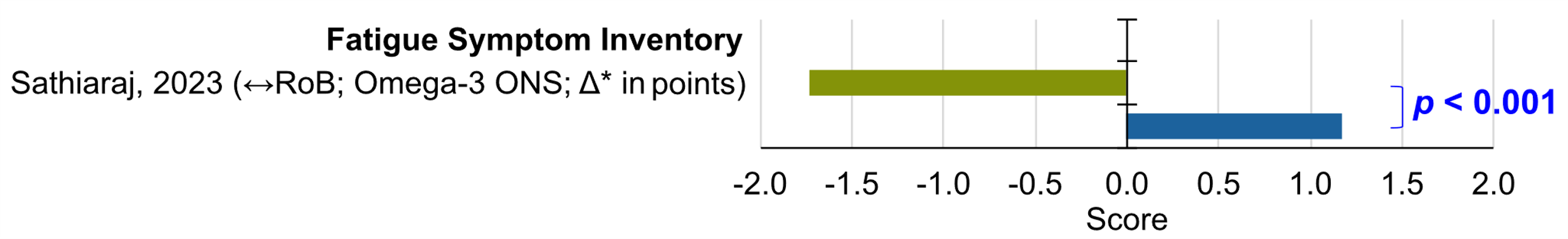


*P*-value in blue represents testing of differences between experimental and control groups. Bolded p-values are statistically significant. Δ* represents absolute mean change from baseline. Abbreviations: Arg, arginine; BCAA, branched-chain amino acids; Gln, glutamine; HMB, *β*-hydroxy *β*-methylbutyrate; ONS, oral nutritional supplement; ↓RoB, low risk of bias; ↔ RoB, moderate risk of bias; ↑ RoB, high risk of bias; TACE, transarterial chemoembolization.

## Supplemental Figure 16. Cancer therapy modifications within high-protein supplementation (green bar) and control (blue bar) groups.


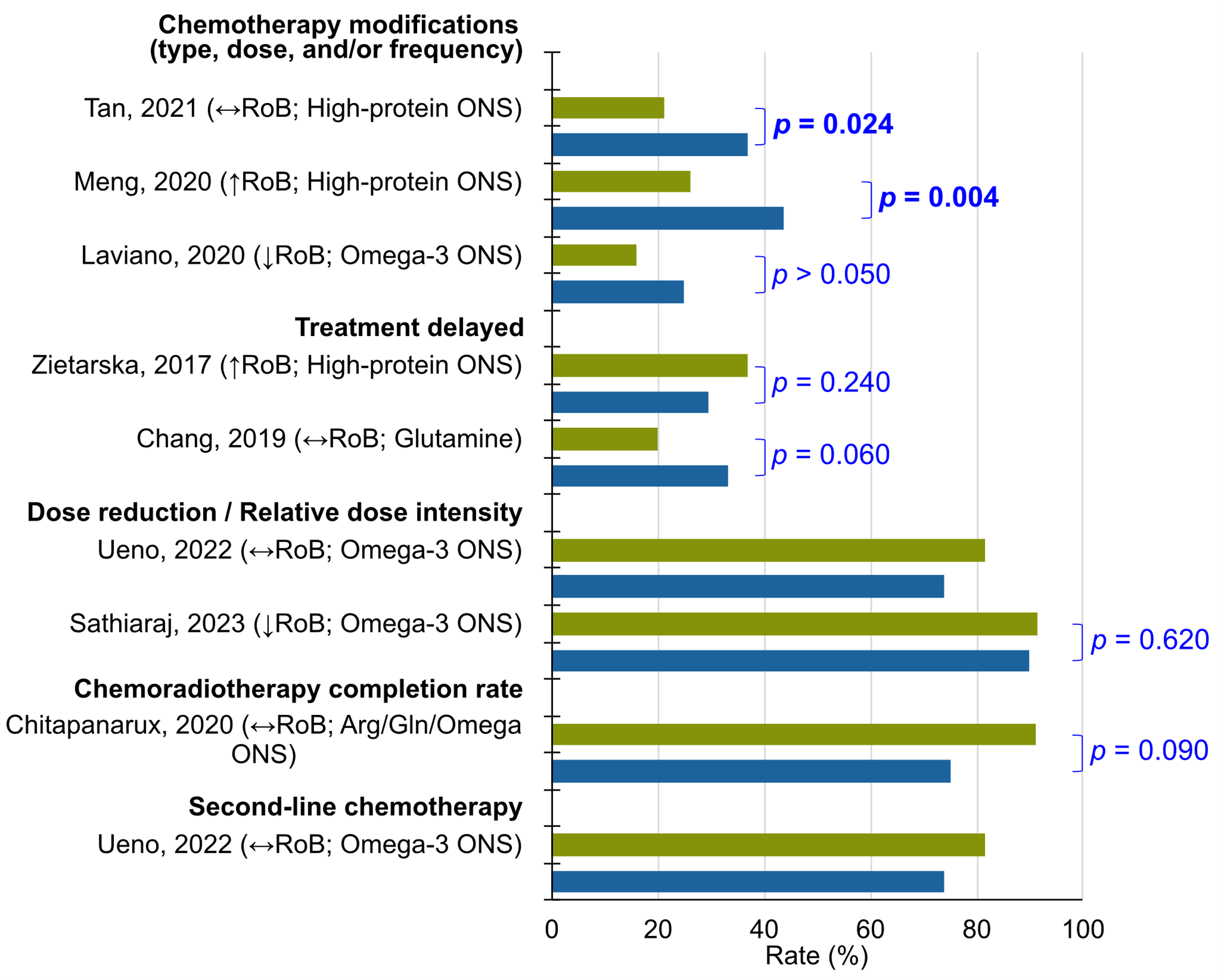

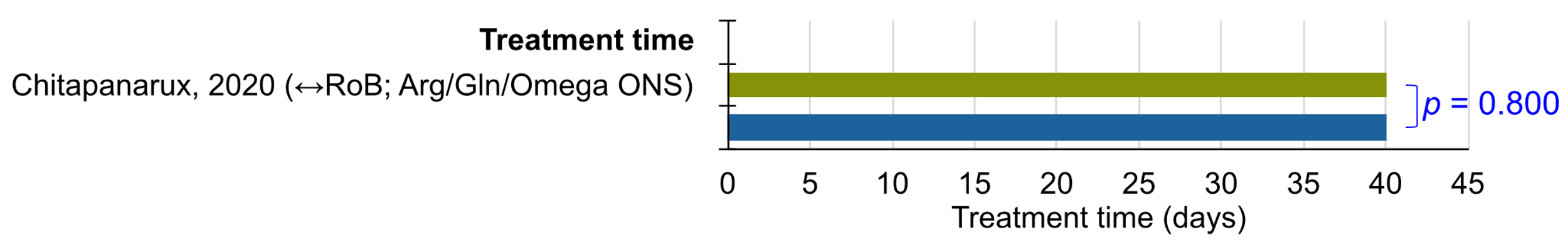


*P*-value in blue represents testing of differences between experimental and control groups. Bolded p-values are statistically significant. Abbreviations: Arg, arginine; BCAA, branched-chain amino acids; Gln, glutamine; ONS, oral nutritional supplement; ↓RoB, low risk of bias; ↔ RoB, moderate risk of bias; ↑ RoB, high risk of bias.

## Supplemental Figure 17. Tumor response at follow-up within high-protein supplementation (green bar) and control (blue bar) groups.


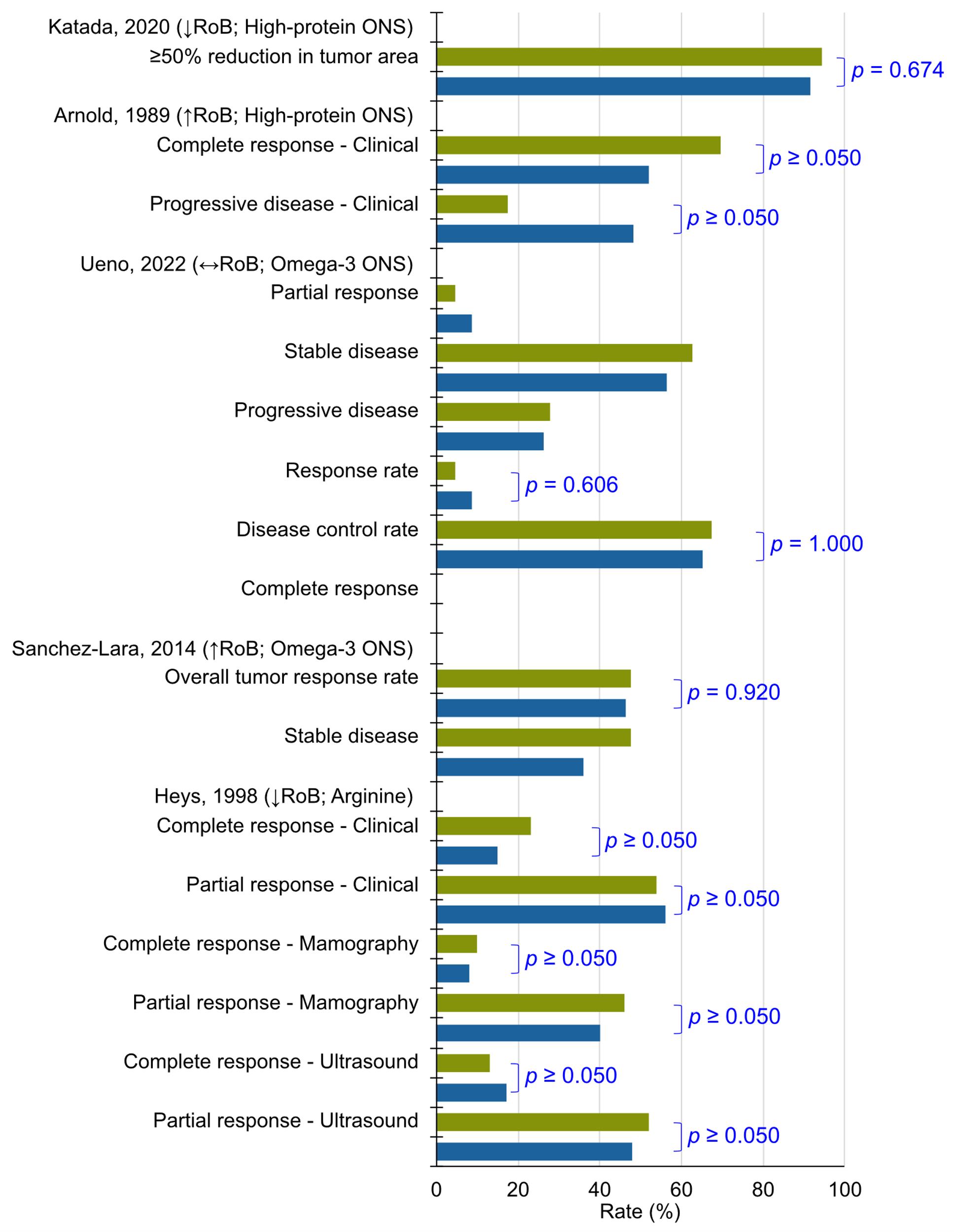

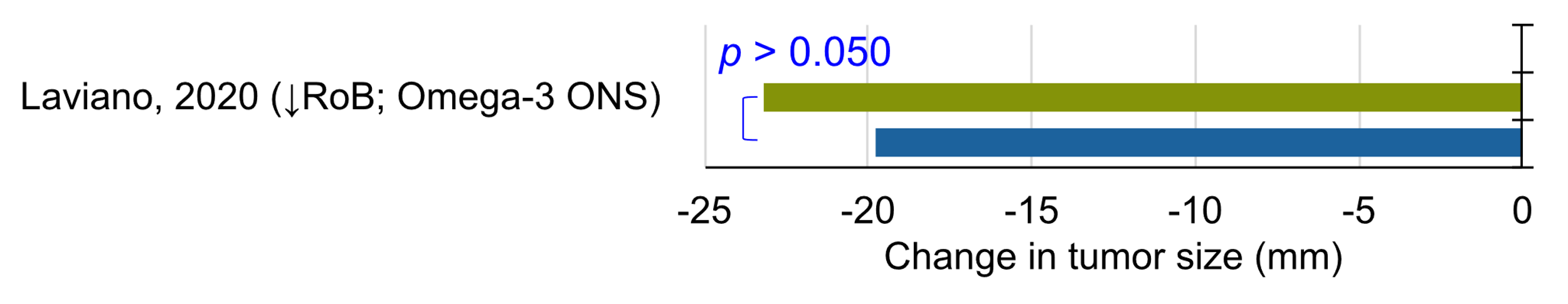


*P*-value in blue represents testing of differences between experimental and control groups. Abbreviations: ONS, oral nutritional supplement; ↓RoB, low risk of bias; ↔ RoB, moderate risk of bias; ↑ RoB, high risk of bias.

## Supplemental Figure 18. Changes in inflammation markers from baseline to follow-up within high-protein supplementation (green bar) and control (blue bar) groups.


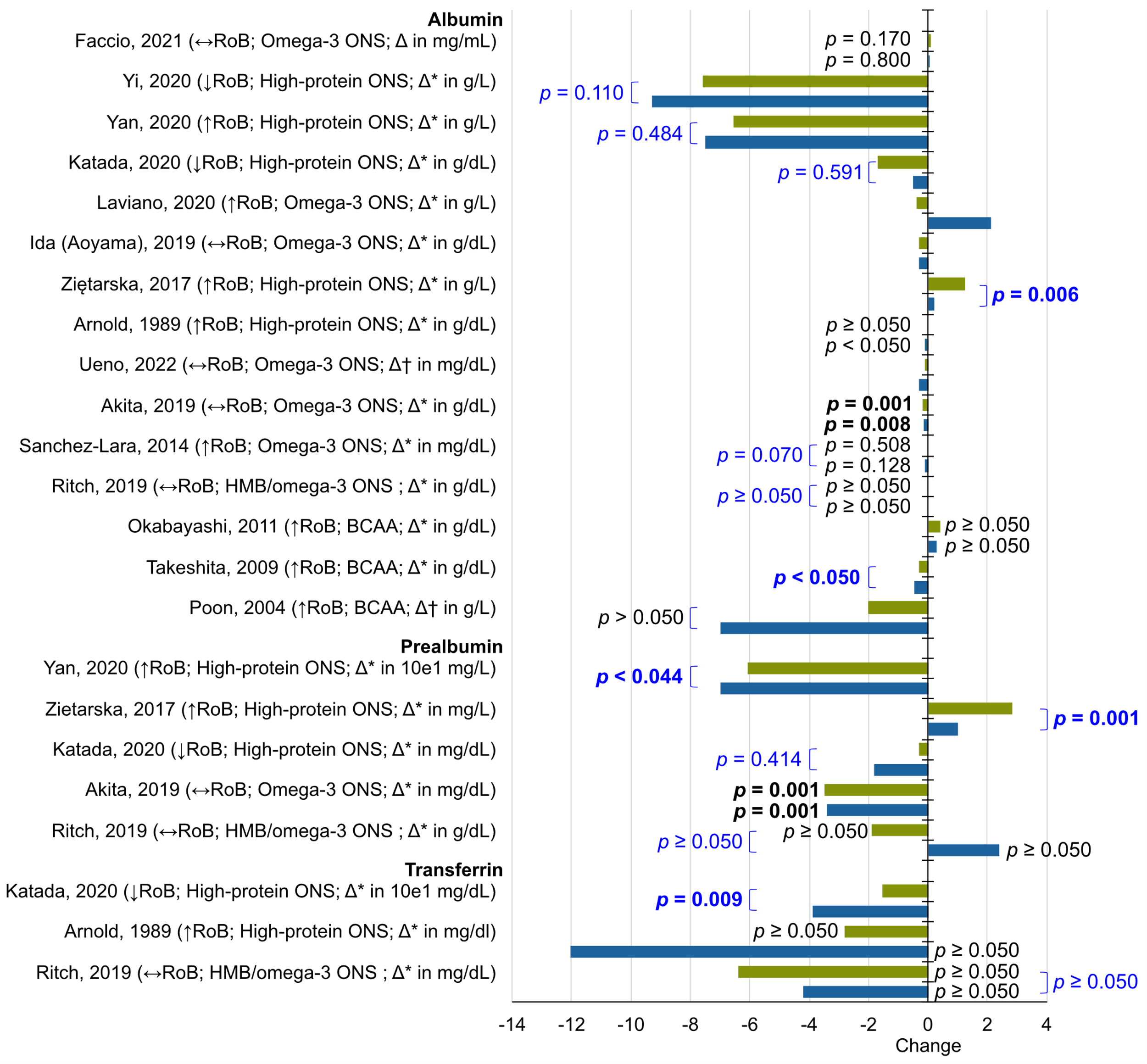

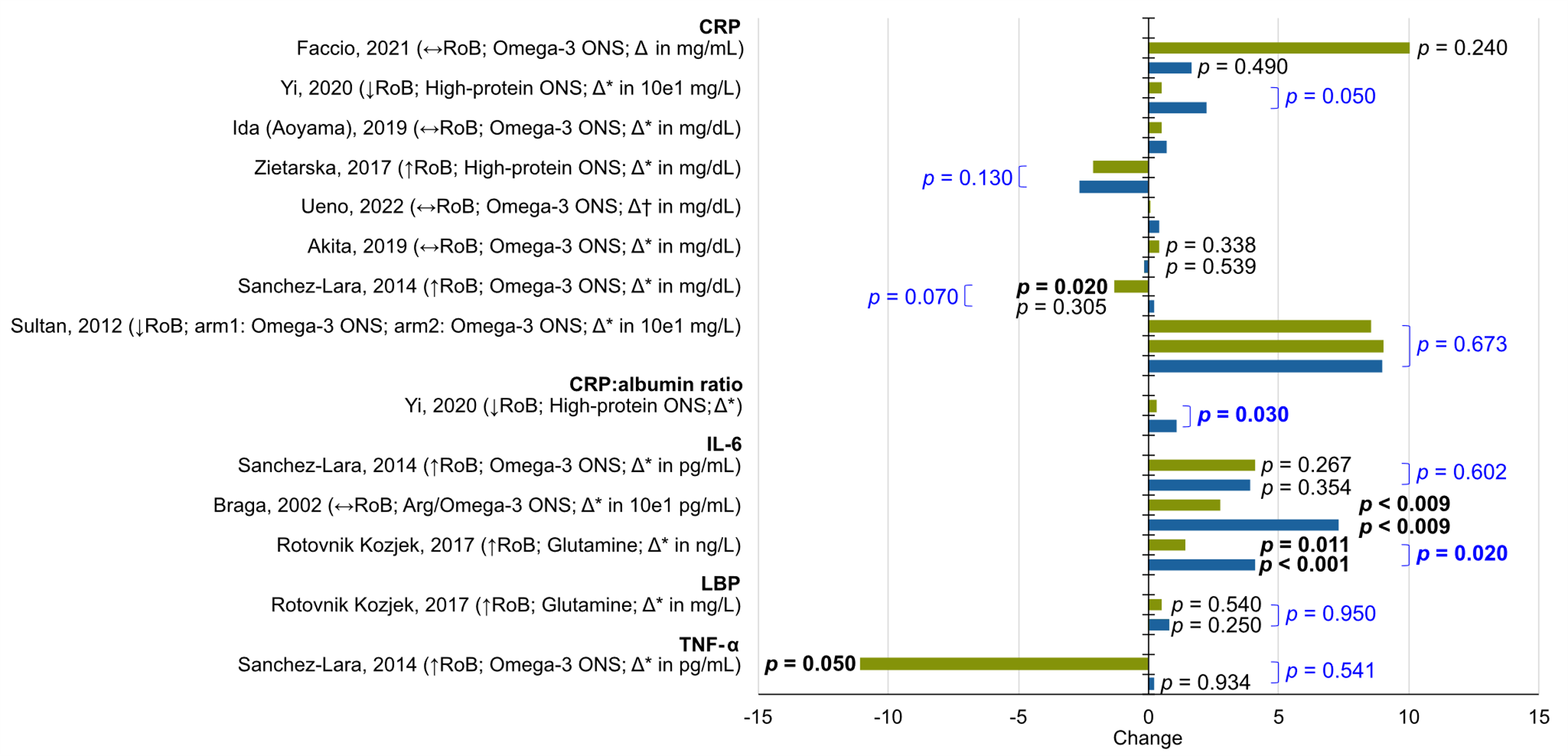


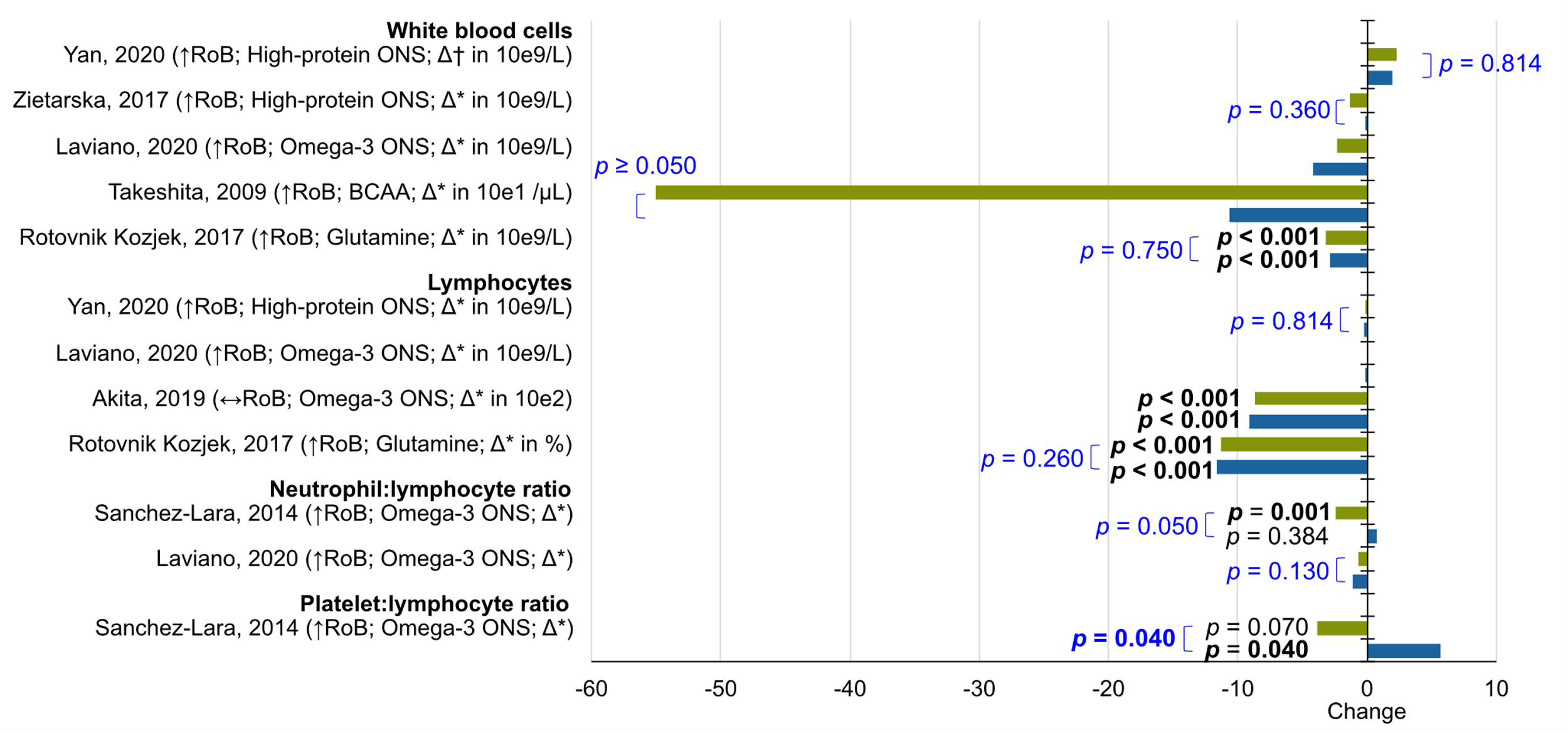

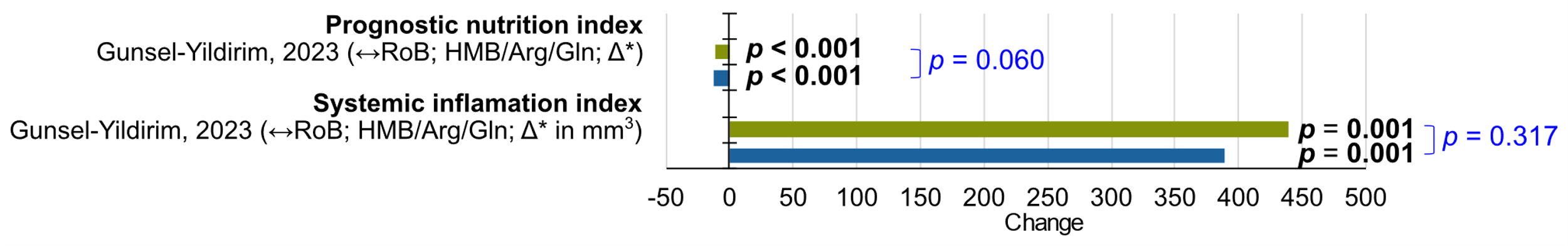


Δ* and Δ† represent absolute or percent mean and median change, respectively. *P*-value in black corresponds to testing of differences from baseline to follow-up within each group. *P*-value in blue represents testing of differences between experimental and control groups. Bolded p-values are statistically significant. Abbreviations: Arg, arginine; BCAA, branched-chain amino acids; CRP, c-reactive protein; Gln, glutamine; HMB, *β*-hydroxy *β*-methylbutyrate; IL-6, interleukin-6; LBP, lipopolysaccharide binding protein; ONS, oral nutritional supplement; ↓RoB, low risk of bias; ↔ RoB, moderate risk of bias; ↑ RoB, high risk of bias; TNF-α, tumor necrosis factor alpha.

## Supplemental Figure 19. Subgroup meta-analyses of the effects of high-protein supplementation on body weight (in kg) based on (A) adherence to prescribed high-protein supplementation regimen, and (B) risk-of-bias of included studies.


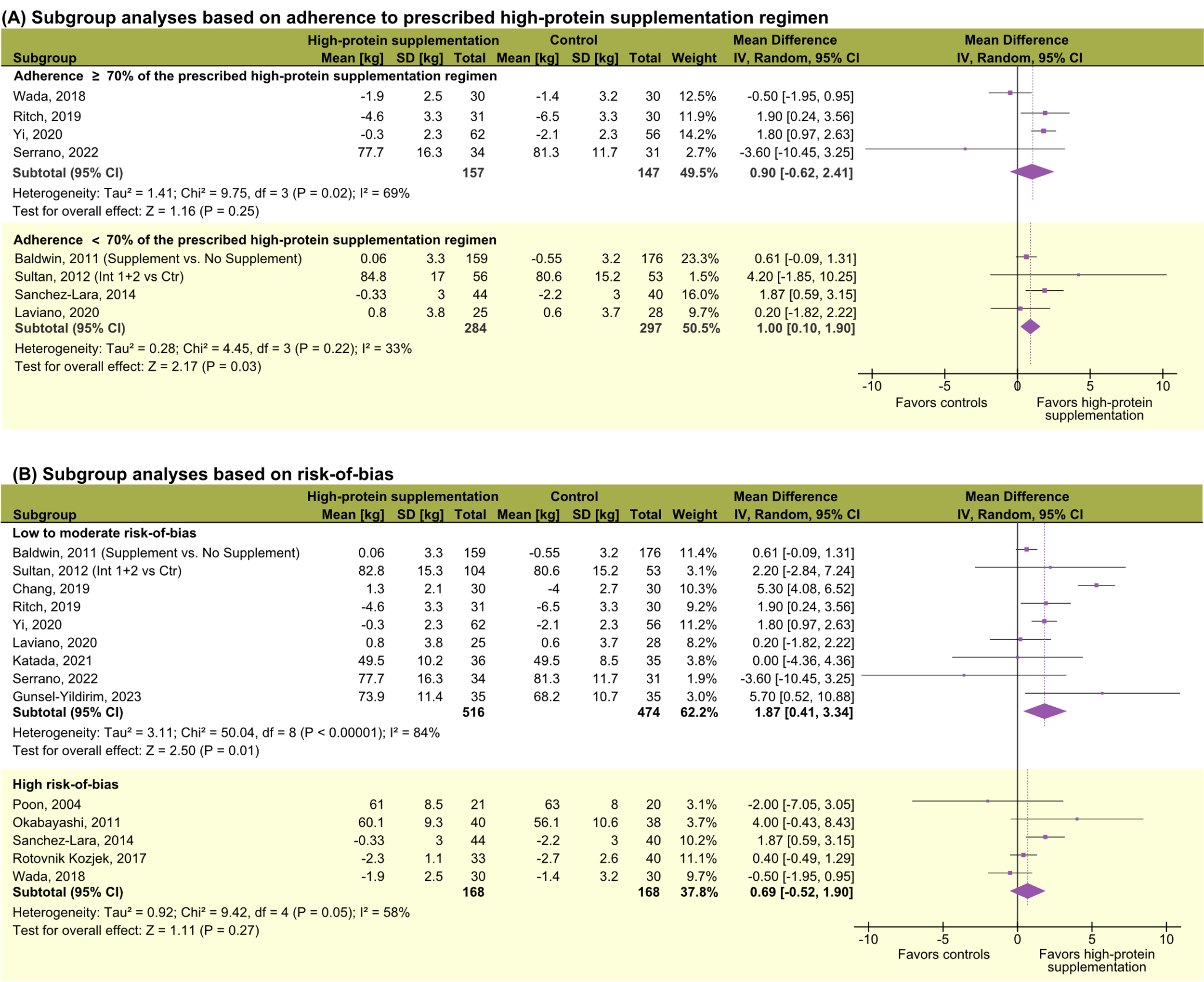


## Supplemental Figure 20. Graphical abstract illustrating the overall findings of this systematic review on the effects of high-protein supplementation during cancer therapy. The number of studies showing a beneficial effect on each outcome is depicted in colored boxes, stratified by different supplement types and relative to the total number of studies analyzed per supplement. The remaining percentages in each box represent studies with mixed findings or no beneficial effect. Dashed boxes represent all supplements combined. Panel A shows all studies analyzed regardless of study quality, while Panel B shows only higher-quality studies (i.e., those with low to moderate risk of bias).

**(A)**

**
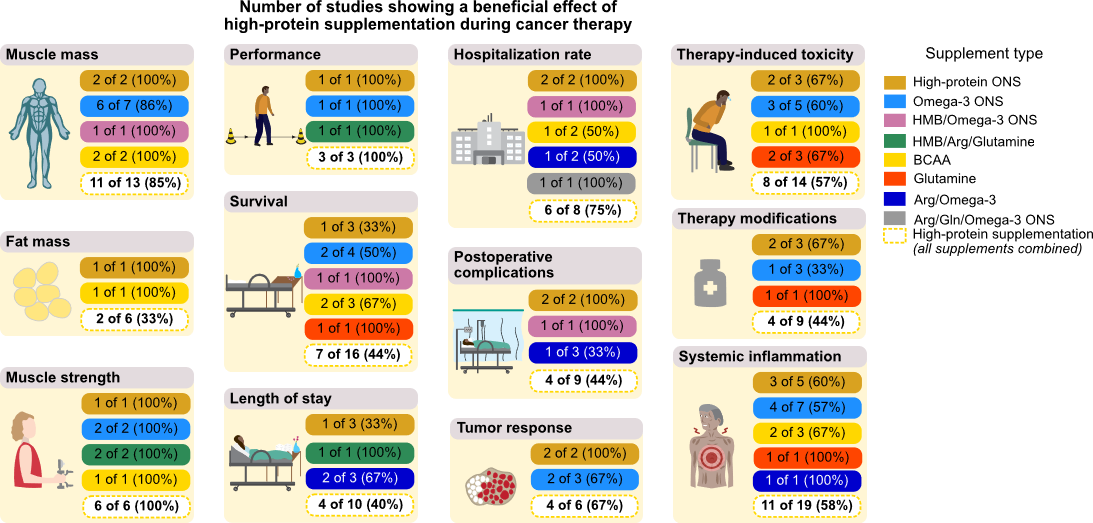
**

**(B)**

**
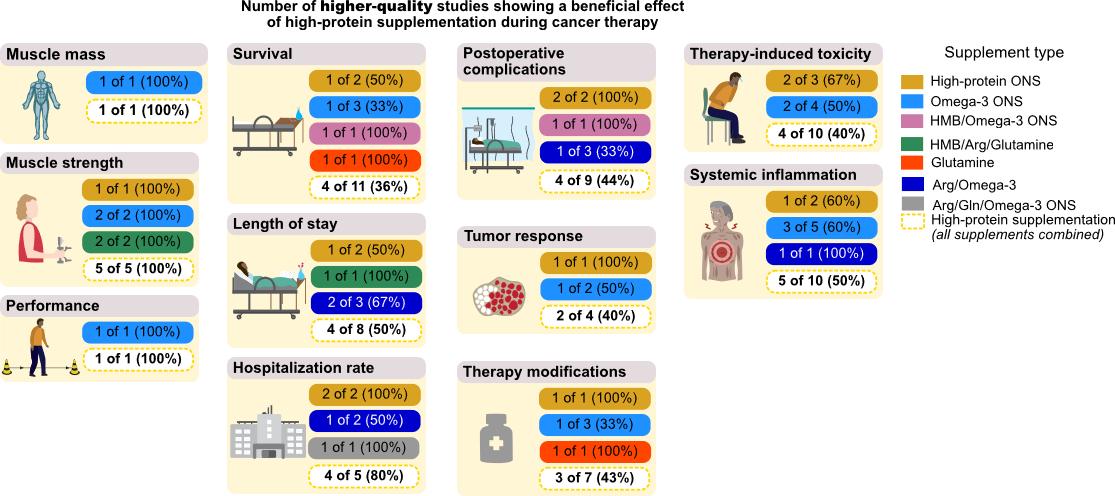
**

Abbreviation list: Arg, arginine; BCAA, branched chain amino acids; Gln, glutamine; HMB, β-hydroxy β-methylbutyrate; ONS, oral nutritional supplement.

## References

1. Sathiaraj E, Afshan K, Sruthi R, Jadoni A, Murugan K, Patil S, et al. Effects of a plant-based high-protein diet on fatigue in breast cancer patients undergoing adjuvant chemotherapy–a randomized controlled trial. Nutr Cancer. 2023;75(3):846–56.

2. Gunsel-Yildirim G, Ceylan KC, Dikmen D. The effect of perioperative immunonutritional support on nutritional and inflammatory status in patients undergoing lung cancer surgery: a prospective, randomized controlled study. Support Care Cancer. 2023;31(6):365.

3. Serrano PE, Parpia S, Simunovic M, Duceppe E, Pinto-Sanchez MI, Bhandari M, et al. Perioperative optimization with nutritional supplements in patients undergoing gastrointestinal surgery for cancer: A randomized, placebo-controlled feasibility clinical trial. Surgery (United States). 2022;172(2):670–6.

4. Ueno M, Sugimori K, Taguri M, Ohkawa S, Kobayashi S, Miwa H, et al. Randomized phase II study of gemcitabine monotherapy vs. gemcitabine with an EPA-enriched oral supplement in advanced pancreatic cancer. Nutr Cancer. 2022;74(1):122–30.

5. Tan S, Meng Q, Jiang Y, Zhuang Q, Xi Q, Xu J, et al. Impact of oral nutritional supplements in post-discharge patients at nutritional risk following colorectal cancer surgery: a randomised clinical trial. Clin Nutr. 2021;40(1):47–53.

6. Katada C, Fukazawa S, Sugawara M, Sakamoto Y, Takahashi K, Takahashi A, et al. Randomized study of prevention of gastrointestinal toxicities by nutritional support using an amino acid-rich elemental diet during chemotherapy in patients with esophageal cancer (KDOG 1101). Esophagus. 2021;18(2):296–305.

7. Meng Q, Tan S, Jiang Y, Han J, Xi Q, Zhuang Q, et al. Post-discharge oral nutritional supplements with dietary advice in patients at nutritional risk after surgery for gastric cancer: a randomized clinical trial. Clin Nutr. 2021;40(1):40–6.

8. Yan X, Liu L, Zhang Y, Song T, Liang Y, Liu Z, et al. Perioperative enteral nutrition improves postoperative recovery for patients with primary liver cancer: a randomized controlled clinical trial. Nutr Cancer. 2021;73(10):1924–32.

9. Baldwin C, Spiro A, McGough C, Norman AR, Gillbanks A, Thomas K, et al. Simple nutritional intervention in patients with advanced cancers of the gastrointestinal tract, non-small cell lung cancers or mesothelioma and weight loss receiving chemotherapy: a randomised controlled trial. J Hum Nutr Diet. 2011;24(5):431–40.

10. Ida S, Hiki N, Cho H, Sakamaki K, Ito S, Fujitani K, et al. Randomized clinical trial comparing standard diet with perioperative oral immunonutrition in total gastrectomy for gastric cancer. Br J Surg. 2017;104(4):377–83.

11. Aoyama T, Yoshikawa T, Ida S, Cho H, Sakamaki K, Ito Y, et al. Effects of perioperative eicosapentaenoic acid-enriched oral nutritional supplement on lean body mass after total gastrectomy for gastric cancer. J Cancer. 2019;10(5):1070–6.

12. Ziętarska M, Krawczyk-Lipiec J, Kraj L, Zaucha R, Małgorzewicz S. Chemotherapy-related toxicity, nutritional status and quality of life in precachectic oncologic patients with, or without, high protein nutritional support. A prospective, randomized study. Nutrients. 2017;9(10):1108.

13. Yi HC, Ibrahim Z, Zaid ZA, ‘Azuan Mat Daud Z, Nor NB, Omar J, et al. Impact of enhanced recovery after surgery with preoperative whey protein-infused carbohydrate loading and postoperative early oral feeding among surgical gynecologic cancer patients: An open-labelled randomized controlled trial. Nutrients. 2020;12(1):264.

14. Faccio AA, Mattos CHP de S, Santos EAS dos, Neto NRM, Moreira RP, Batella LT, et al. Oral nutritional supplementation in cancer patients who were receiving chemo/chemoradiation therapy: a multicenter, randomized phase II study. Nutr Cancer. 2021;73(3):442–9.

15. Arnold C, Richter MP. The effect of oral nutritional supplements on head and neck cancer. Int J Radiat Oncol Biol Phys. 1989;16(6):1595–9.

16. Poon RTP, Yu WC, Fan ST, Wong J. Long-term oral branched chain amino acids in patients undergoing chemoembolization for hepatocellular carcinoma: a randomized trial. Aliment Pharmacol Ther. 2004;19(7):779–88.

17. Takeshita S, Ichikawa T, Nakao K, Miyaaki H, Shibata H, Matsuzaki T, et al. A snack enriched with oral branched-chain amino acids prevents a fall in albumin in patients with liver cirrhosis undergoing chemoembolization for hepatocellular carcinoma. Nutr Res. 2009;29(2):89–93.

18. Okabayashi T, Iyoki M, Sugimoto T, Kobayashi M, Hanazaki K. Oral supplementation with carbohydrate- and branched-chain amino acid-enriched nutrients improves postoperative quality of life in patients undergoing hepatic resection. Amino Acids. 2011;40(4):1213–20.

19. Chitapanarux I, Traisathit P, Chitapanarux T, Jiratrachu R, Chottaweesak P, Chakrabandhu S, et al. Arginine, glutamine, and fish oil supplementation in cancer patients treated with concurrent chemoradiotherapy: a randomized control study. Curr Probl Cancer. 2020;44(1):100482.

20. Poulsen GM, Pedersen LL, Østerlind K, Bæksgaard L, Andersen JR. Randomized trial of the effects of individual nutritional counseling incancer patients. Clinical Nutrition [Internet]. 2014;33(5):749–53. Available from: http://dx.doi.org/10.1016/j.clnu.2013.10.019

21. Sánchez-Lara K, Turcott JG, Juárez-Hernández E, Nuñez-Valencia C, Villanueva G, Guevara P, et al. Effects of an oral nutritional supplement containing eicosapentaenoic acid on nutritional and clinical outcomes in patients with advanced non-small cell lung cancer: randomised trial. Clin Nutr. 2014;33(6):1017–23.

22. Moya P, Miranda E, Soriano-Irigaray L, Arroyo A, Aguilar M del M, Bellón M, et al. Perioperative immunonutrition in normo-nourished patients undergoing laparoscopic colorectal resection. Surg Endosc. 2016;30(11):4946–53.

23. Sultan J, Griffin SM, Di Franco F, Kirby JA, Shenton BK, Seal CJ, et al. Randomized clinical trial of omega-3 fatty acid-supplemented enteral nutrition versus standard enteral nutrition in patients undergoing oesophagogastric cancer surgery. Br J Surg. 2012;99(3):346–55.

24. Akita H, Takahashi H, Asukai K, Tomokuni A, Wada H, Marukawa S, et al. The utility of nutritional supportive care with an eicosapentaenoic acid (EPA)-enriched nutrition agent during pre-operative chemoradiotherapy for pancreatic cancer: prospective randomized control study. Clin Nutr ESPEN. 2019;33:148–53.

25. Laviano A, Calder PC, Schols AMWJ, Lonnqvist F, Bech M, Muscaritoli M. Safety and tolerability of targeted medical nutrition for cachexia in non-small-cell lung cancer: a randomized, double-blind, controlled pilot trial. Nutr Cancer. 2020;72(3):439–50.

26. Braga M, Gianotti L, Vignali A, Di Carlo V. Preoperative oral arginine and n-3 fatty acid supplementation improves the immunometabolic host response and outcome after colorectal resection for cancer. Surgery. 2002;132(5):805–14.

27. Gianotti L, Braga M, Nespoli L, Radaelli G, Beneduce A, Di Carlo V. A randomized controlled trial of preoperative oral supplementation with a specialized diet in patients with gastrointestinal cancer. Gastroenterology. 2002;122(7):1763–70.

28. Rotovnik Kozjek N, Kompan L, Žagar T, Mrevlje. Influence of enteral glutamine on inflammatory and hormonal response in patients with rectal cancer during preoperative radiochemotherapy. Eur J Clin Nutr. 2017;71(5):671–3.

29. Wada N, Kurokawa Y, Tanaka K, Miyazaki Y, Makino T, Takahashi T, et al. Perioperative nutritional support with beta-hydroxy-beta-methylbutyrate, arginine, and glutamine in surgery for abdominal malignancies. Wounds. 2018;30(9):251–6.

30. Chang SC, Lai YC, Hung JC, Chang CY. Oral glutamine supplements reduce concurrent chemoradiotherapy-induced esophagitis in patients with advanced non-small cell lung cancer. Medicine (Baltimore). 2019;98(8):e14463.

31. Pandey M, Gaurav K, Singh S, Goel RK. Safety and efficacy of oral and parenteral glutamine supplementation in reducing chemotherapy induced toxicities in patients with breast cancer receiving chemotherapy: Results of interim analysis of a prospective, randomized, three arm, placebo-controlled. World J Surg. 2012;1(4):17–27.

32. Ritch CR, Cookson MS, Clark PE, Chang SS, Fakhoury K, Ralls V, et al. Perioperative oral nutrition supplementation reduces prevalence of sarcopenia following radical cystectomy: results of a prospective randomized controlled trial. J Urol. 2019;201:470–7.

33. Choi K, Lee SS, Oh SJ, Lim SY, Lim SY, Jeon WK, et al. The effect of oral glutamine on 5-fluorouracil/leucovorin-induced mucositis/stomatitis assessed by intestinal permeability test. Clin Nutr. 2007;26(1):57–62.

34. Heys SD, Ogston K, Miller I, Hutcheon AW, Walker LG, Sarker TK, et al. Potentiation of the response to chemotherapy in patients with breast cancer by dietary supplementation with L-arginine: results of a randomised controlled trial. Int J Oncol. 1998;12(1):221–5.

35. (a) Ravasco P, Monteiro-Grillo I, Vidal PM, Camilo ME. Dietary counseling improves patient outcomes: A prospective, randomized, controlled trial in colorectal cancer patients undergoing radiotherapy. J Clin Oncol. 2005;23(7):1431–8.

36. Ravasco P, Monteiro-Grillo I, Camilo M. Individualized nutrition intervention is of major benefit to colorectal cancer patients: Long-term follow-up of a randomized controlled trial of nutritional therapy. Am J Clin Nutr. 2012;96(6):1346–53.

37. (b) Ravasco P, Monteiro-Grillo I, Vidal PM, Camilo ME. Impact of nutrition on outcome: A prospective randomized controlled trial in patients with head and neck cancer undergoing radiotherapy. Head Neck. 2005;27(8):659–68.
