# Supplementary material for: Effects of high-protein supplementation during cancer therapy: a systematic review and meta-analysis
Source: Am J Clin Nutr. 2024 Dec 2;120(6):1311–24. doi: 10.1016/j.ajcnut.2024.08.016 (PMC11619795; doi:10.1016/j.ajcnut.2024.08.016)
Supplement: multimedia component 2 [file mmc2.pptx]

## Slide 1
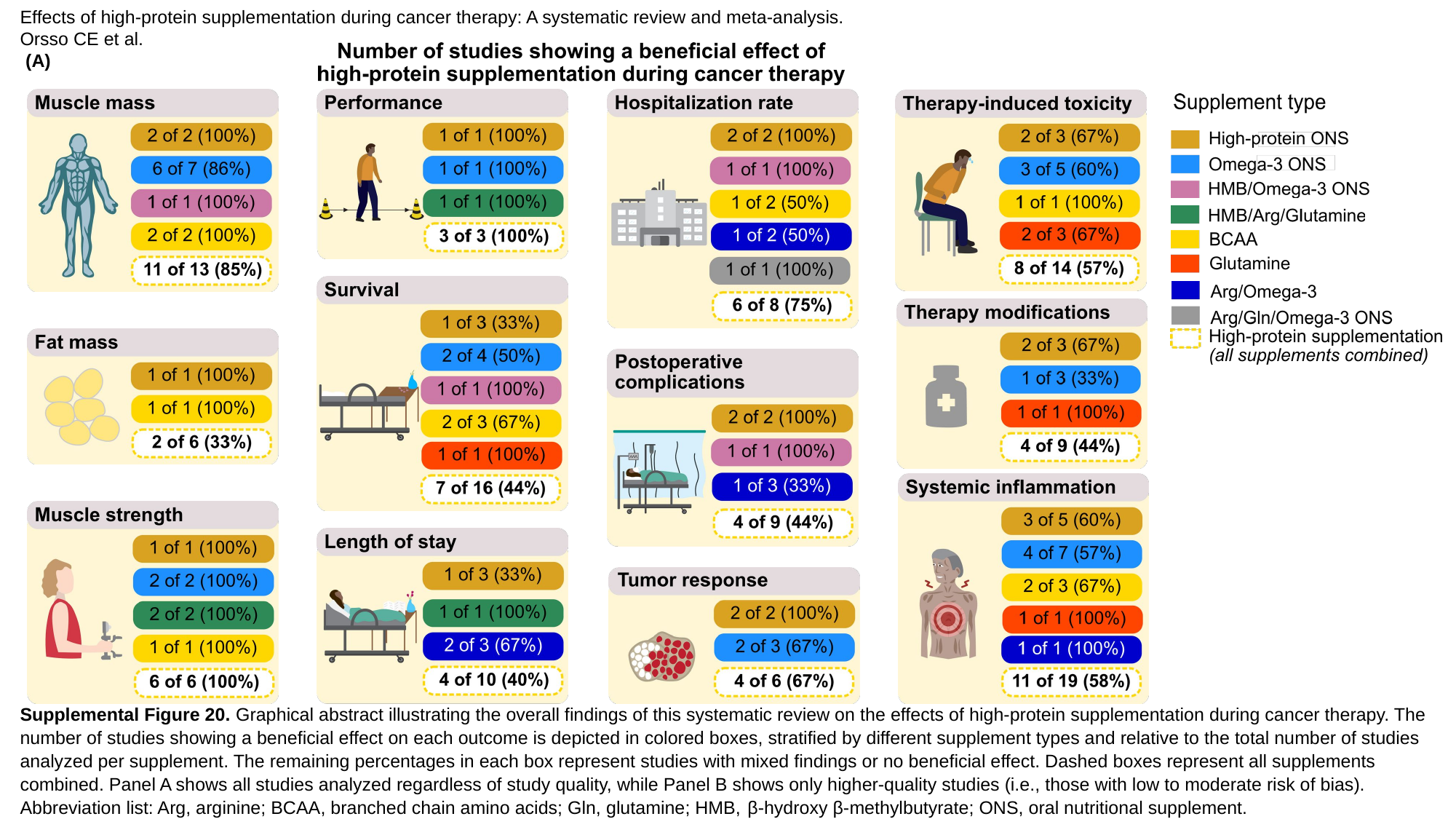

Effects of high-protein supplementation during cancer therapy: A systematic review and meta-analysis.
Orsso CE et al.
(A)
Supplemental Figure 20. Graphical abstract illustrating the overall findings of this systematic review on the effects of high-protein supplementation during cancer therapy. The number of studies showing a beneficial effect on each outcome is depicted in colored boxes, stratified by different supplement types and relative to the total number of studies analyzed per supplement. The remaining percentages in each box represent studies with mixed findings or no beneficial effect. Dashed boxes represent all supplements combined. Panel A shows all studies analyzed regardless of study quality, while Panel B shows only higher-quality studies (i.e., those with low to moderate risk of bias). Abbreviation list: Arg, arginine; BCAA, branched chain amino acids; Gln, glutamine; HMB, β-hydroxy β-methylbutyrate; ONS, oral nutritional supplement.

## Slide 2
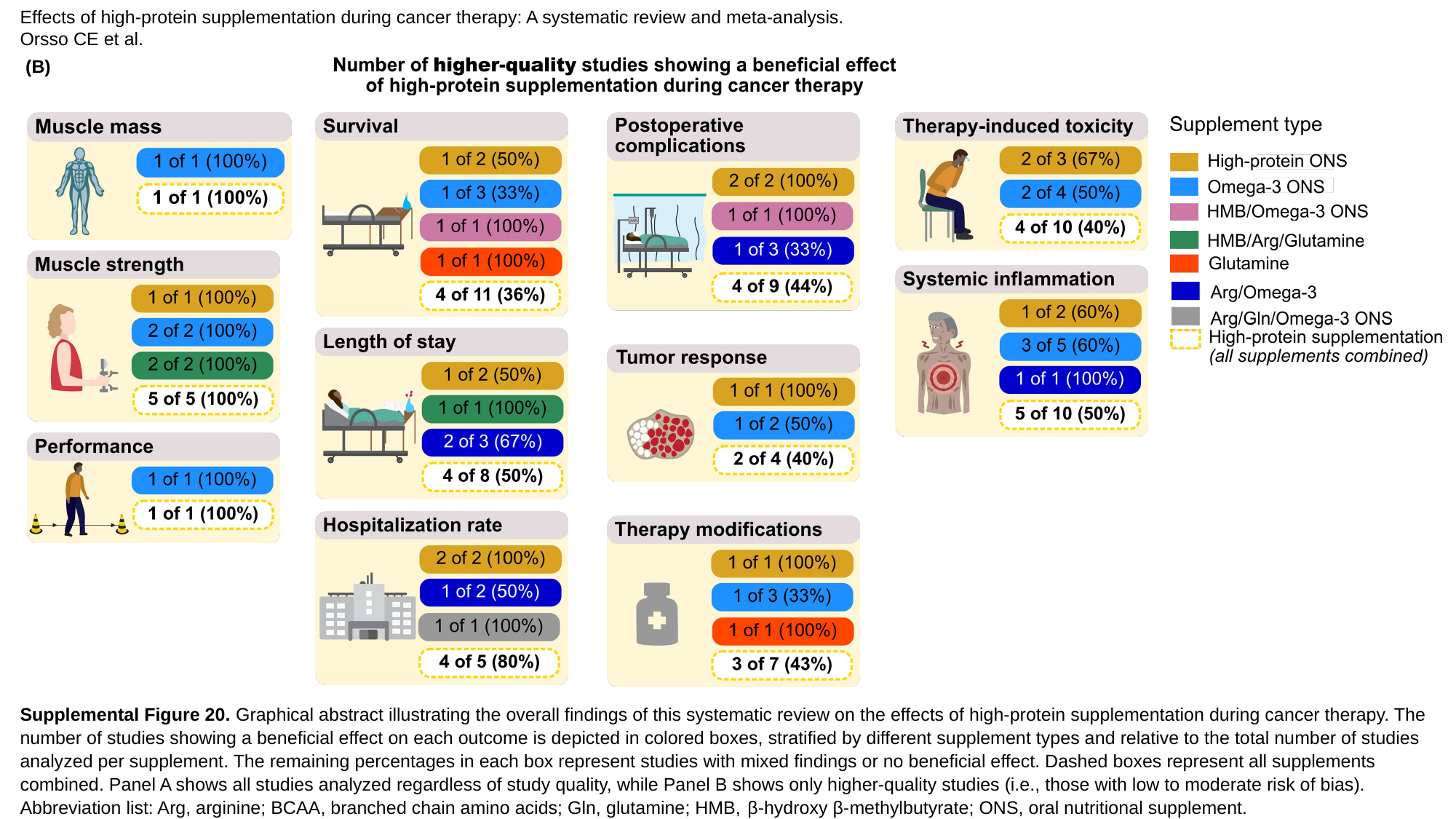

Effects of high-protein supplementation during cancer therapy: A systematic review and meta-analysis.
Orsso CE et al.
(B)
Supplemental Figure 20. Graphical abstract illustrating the overall findings of this systematic review on the effects of high-protein supplementation during cancer therapy. The number of studies showing a beneficial effect on each outcome is depicted in colored boxes, stratified by different supplement types and relative to the total number of studies analyzed per supplement. The remaining percentages in each box represent studies with mixed findings or no beneficial effect. Dashed boxes represent all supplements combined. Panel A shows all studies analyzed regardless of study quality, while Panel B shows only higher-quality studies (i.e., those with low to moderate risk of bias). Abbreviation list: Arg, arginine; BCAA, branched chain amino acids; Gln, glutamine; HMB, β-hydroxy β-methylbutyrate; ONS, oral nutritional supplement.
